# DepoCatalog: mapping diversity of 129 recombinantly produced *Klebsiella* phage depolymerases

**DOI:** 10.1038/s41467-026-73570-7

**Published:** 2026-05-22

**Authors:** Aleksandra Otwinowska, Sebastian Olejniczak, Agnieszka Latka, Maria Pozniak, Grazyna Majkowska-Skrobek, Barbara Maciejewska, Janusz Koszucki, Vyshakh R. Panicker, Sara Jablonska, Mathilde Hulsens, Jana Stender, Maha Niazi, Sabrina Green, Joachim J. Bugert, Régis Tournebize, Stan J. J. Brouns, Flavia Squeglia, Rita Berisio, Jens A. Hammerl, Rob Lavigne, Yves Briers, Rafal J. Mostowy, Zuzanna Drulis-Kawa

**Affiliations:** 1 Department of Pathogen Biology and Immunology, University of Wroclaw, Wroclaw, Poland; 2 Department of Biotechnology, Ghent University, Ghent, Belgium; 3 Malopolska Centre of Biotechnology, Jagiellonian University, Kraków, Poland; 4 Doctoral School of Exact and Natural Sciences, Jagiellonian University, Kraków, Poland; 5 Bundeswehr Institute of Microbiology, Munich, Germany; 6 Department of Biosystems, KU Leuven, Leuven, Belgium; 7 Sorbonne Université, INSERM, Centre d’Immunologie et des Maladies Infectieuses, CIMI, Paris, France; 8 UTechS Photonic BioImaging Direction de la Technologie, Centre de Recherche et de Ressources Technologiques, Institut Pasteur, Paris, France; 9 Department of Bionanoscience, Delft University of Technology, Delft, The Netherlands; 10 Institute of Biostructures and Bioimaging, CNR, Naples, Italy; 11 Department Biological Safety, German Federal Institute for Risk Assessment, Berlin, Germany

**Keywords:** Phage biology, Bacteriophages, Enzymes

## Abstract

Our understanding of how depolymerase sequence and structure determine substrate specificity is fragmentary due to the limited number of experimentally characterized enzymes. Here we show DepoCatalog - an experimentally validated collection of 129 recombinantly prepared *Klebsiella* phage depolymerases (90 enzymes produced in this study and 39 homologs from the literature), with specificity spanning 75 KL-types. Enzymes originated from podo-, sipho-, myo-, jumbo phages, and prophages. Using activity profiling, structural modeling, and domain dissection, we propose a five‑class framework that captures the architectural and functional diversity of these enzymes. DepoCatalog uncovers cross-reactivity and taxa‑specific enzymes. Structural comparisons indicate that specificity switching or extension is associated with modifications to the C‑terminal domain. We further hypothesize that podoviruses encoding up to two RBPs show greater receptor adaptability than jumbo phages with multiple specialized RBPs. Finally, we develop a publicly accessible, DepoCat dataset (https://depocat.uwr.edu.pl) for specificity, structural classification and comparison of newly identified depolymerases.

## Abstract (web summary)

Phages can use enzymes (depolymerases) that degrade capsular polysaccharides to initiate infection of their bacterial hosts. Here, the authors characterize a diverse set of 129 *Klebsiella* phage depolymerases, defining five enzyme classes, revealing cross-reactivity, and linking enzyme’s specificity to structure.

## Introduction

Capsular polysaccharides (CPS) are complex carbohydrate structures that form a dense, protective layer surrounding encapsulated bacteria, contributing to multiple aspects of physiology and pathogenicity, mimicking host cell molecules, and shielding pathogens from host immune recognition and defense mechanisms^1–5^. It also protects bacteria from recognition and infection by bacterial viruses^6,7^. The most well-known encapsulated Gram-negative pathogen is *Klebsiella pneumoniae*^1,8,9^. To date, at least 163 distinct *cps* loci encoding *Klebsiella* CPS biosynthetic proteins have been identified, including the classical K1-K82 serotypes (KL1-KL82 loci), as well as at least 86 additional, genetically divergent KL-loci (KL101–KL186)^10–12^. Despite the resolution of at least 76 serologically defined CPS structures, the monosaccharide composition and higher-order architecture of many capsular polysaccharides remain experimentally unverified^13^ with some exceptions (KL102, KL108, and KL112)^14,15^. This gap in structural knowledge significantly limits our understanding of their structural relationships and potential antigenic, virulence, or immunogenic properties.

Many *Klebsiella* phages developed virion-associated enzymes (depolymerases) that degrade CPS layers to initiate infection^2,3,16–19^. These enzymes, which are typically receptor-binding proteins (RBPs), differ in their organization, ranging from single-type RBPs to complex branching systems (2–14 RBPs)^16,20,21^. Phage-encoded depolymerases described so far exhibit a conserved modular architecture and typically assemble into elongated homotrimers centered around a parallel β-helix core^22–25^. Their structure comprises three principal domains: (i) an N-terminal structural region, (ii) a central enzymatic domain, and (iii) a C-terminal trimerization module, also contributing to substrate recognition^26–29^. Certain RBPs (e.g., KP32gp37) are equipped with an intramolecular chaperon at the C-terminus, guiding proper folding and possibly undergoing autoproteolytic cleavage upon trimer assembly^3,30^ or can possess additional insertion domains involved in active site architecture^31–34^.

Our understanding of the relationship between sequence, structure, and substrate specificity remains fragmentary since the number of experimentally characterized depolymerases is limited^35,36^. Although some enzymes with high sequence similarity display particular specificity, others with no detectable similarity can target the same capsular polysaccharide^37,38^. Additionally, prophage-encoded depolymerases are largely unexplored, since most available data focus on enzymes from virulent phages^39^.

In this study, we present a catalog of 129 phage-borne depolymerases prepared as recombinant proteins and experimentally verified for their specificity towards *Klebsiella* capsule degradation. Secondly, integrating enzyme specificity, structure prediction with domain dissection analysis, we proposed a five-class enzyme catalog as a conceptual framework for understanding the diversity and evolution of depolymerases. We aimed to answer several research questions about structural and specificity variations of depolymerases, how to predict the capsular tropism, whether diversity of depolymerases reflects the genetic variability of *Klebsiella* CPS loci, how the specificity varies across different replication cycles and morphotypes, and which feature underlies the ability of certain depolymerases to degrade multiple capsular types?

## Results

### Depolymerase identification, testing, and classification strategy

**Fig. 1:**
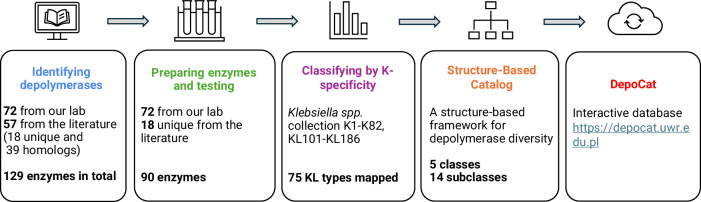
The pipeline of the DepoCatalog study. Identifying depolymerases (first step) - depolymerases were collected from two sources, including 72 enzymes from our laboratory and 57 recombinant depolymerases from the literature with experimentally assigned specificity (comprising 18 unique proteins and 39 homologs to our collection), yielding a total of 129 candidates; Preparing enzymes and testing (second step) - 90 enzymes, including 72 from our laboratory and 18 unique literature-derived proteins were prepared in this study as recombinant proteins and tested for their activity; Classifying by K-specificity (third step) – 90 recombinantly prepared enzymes were experimentally validated and classified according to capsular (K) specificity using a Klebsiella spp. strain collection representing K1–K82 and KL101–KL186 types, resulting in mapping across 75 KL types;  Structure-Based Catalog (fourth step) –  enzymes were organized into a structure-based catalog comprising 5 major classes and 14 subclasses; Depocat (fifth step) - all data were integrated into an interactive database for specificity and structural classification and comparison of newly identified depolymerases (https://depocat.uwr.edu.pl/).

The DepoCatalog study was designed according to a presented pipeline (Fig. 1). In the first step, we identified and characterized 129 phage-borne depolymerases, comprising 72 enzymes derived from virulent phages and prophages in our collection and 57 recombinantly prepared enzymes from the literature. The collected 129 enzymes originate from phages with distinct virion morphologies: podoviruses (46 proteins), siphoviruses (10 proteins), myoviruses (13 proteins), and jumbo myovirus phages (40 proteins). Moreover, 20 proteins were expressed based on Klebsiella prophage genes. It is important to note that this collection was not designed as an exhaustive survey of all publicly available *Klebsiella* phage genomes, therefore, represents a curated rather than complete sampling of depolymerase diversity. The classification framework proposed here should be regarded as provisional and subject to revision as additional sequences are characterized.

In step 2, we produced and tested 72 enzymes from our collection and 18 selected literature-reported depolymerases for which no structural or enzymatic homologs matched our proteins (unique), because we were aware that relying on literature-derived data for a subset of enzymes may underestimate their true host range. The remaining 39 depolymerases reported in previous studies, homologs in sequence/structure with our proteins, were assessed based on available published data. Their enzymatic specificity turned out to be consistent with the results obtained for enzymes from our collection.

All 90 depolymerases prepared here were screened by a spot assay for the ability to degrade the particular capsule of *Klebsiella spp*. strains belonging to K1-K82 defined serotypes or genetically classified as KL101-KL186 with some exceptions (KL106, KL129, KL147, KL150, KL152, KL154, KL156, KL157, KL159, KL160, KL162, KL171, KL172, KL175, KL176, KL179, KL180, KL182, KL185) (Supplementary Data 1). For some KL-types, we include several strains to evaluate the range of protein activity. The panel was designed to include the broadest and most diverse collection of Klebsiella capsule locus (KL) type isolates available to us. Based on this approach, we were able to map enzyme specificity across 75 KL-types (step 3). Among those, 48 additional capsule types were found to be targeted by depolymerases described here, and ~50 enzymes had not previously been linked to any known enzymatic activity. Moreover, based on depolymerase activity testing, further capsule types were assigned as KN3 = KL183, KN4 = KL124, and KN5 = KL121.

The depolymerase activity range was compared with each corresponding phage lytic activity, revealing mostly correlated patterns with some exceptions where halos were formed for enzymes and no detectable phage lysis or halo zone (Supplementary Data 2). This suggests no propagation of phage on a particular host (no plaque ± halo), but the degrading activity of recombinant depolymerase on the CPS of this host (halo formation).

To characterize a curated enzyme catalog, the key characteristics (targeted KL-type; depolymerase name and ID; annotation; nucleotide sequence; amino acid sequence; length in aa; domain features; catalytic site; cleaved bond; tested KL-types; phage name and replication cycle; phage morphotype; phage genome accession number; reference) for all collected depolymerases have been compiled in Supplementary Data 3.

Each protein (129) was then analyzed by structure prediction (AlphaFold3.0) and domain dissection (PyMOL) to investigate the modularity of capsule-degrading enzymes, their specificity, and versatility, to develop a structure-based catalog with 5 main classes and 14 subclasses (step 4). Structural confidence scores were calculated for all enzymes based on a homotrimer structure modelled. The global model quality measures (pTM, ipTM), predicted distance error (PAE) statistics, and average pLDDT values were calculated for entire structures, and for main domains (N-terminal, Central, and C-terminal) (Supplementary Data 4). We considered only the modelled central and C-terminal domains with pLDDT > 70, eliminating the N-terminus (virion-anchoring part of the RBP) from the full homotrimer in PyMOL.

In the final step, the open-access server DepoCat has been created, with an interactive database of depolymerases derived from Klebsiella phages, developed to facilitate both the exploration and structural classification of these enzymes. (https://depocat.uwr.edu.pl).

### Depolymerase specificity versus enzyme diversity

**Fig. 2:**
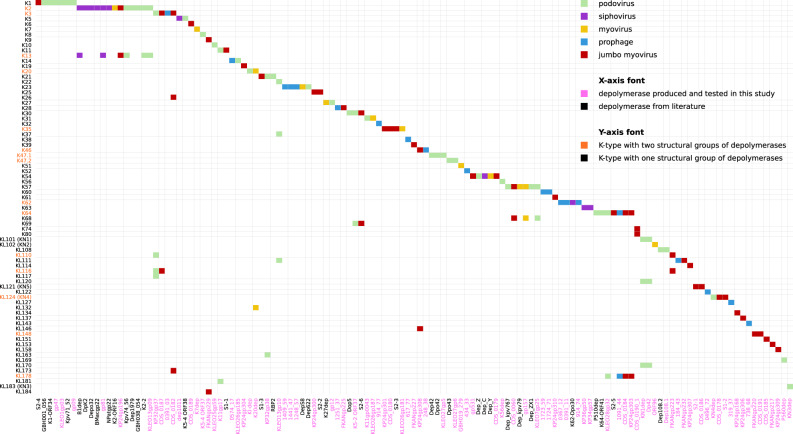
Comparative matrix of 129 phage depolymerase specificity across Klebsiella capsule types tested in a spot assay. Each row represents a distinct K-type, while each column corresponds to an individual phage depolymerase. Color coding indicates phage morphological groups (podovirus in green, siphovirus in purple, myovirus in yellow, jumbo myovirus in red, and prophages in blue). Experimentally validated depolymerases from this study are indicated in pink font alongside previously described enzymes from the literature with assigned specificity (black font). K-type, recognized by two structurally distinct groups of depolymerases, is labelled in orange font.

The CPS-degrading enzymes were first grouped by substrate specificity to assess structural and sequence variability within a given specificity profile and phage origin (Fig. 2; Supplementary Figs. 1−4 and Supplementary Data 3). All defined protein groups were also subjected to sequence (aa) and structural (TM score) alignments carried out on the N-terminal domain-deficient proteins to avoid interpretation bias caused by structure characteristics related to phage genera. In general, an expanded dataset of Klebsiella phage depolymerases tested on the broad KL-type collection displayed diverse K-type coverage and origins (podo-, sipho-, myo-, and jumbo myo- phages). Notably, K-types were targeted by two structurally diverse depolymerase groups (K2/K13, K3, K20, K35, K46, K47, K62, K64/KL178, KL110/KL116, KL124, KL148, KL178).

Considering the substrate specificity range of the individual enzyme, it turned out that the vast majority of proteins showed narrow activity only to a single capsule type. Some enzymes were able to degrade the capsule of strains belonging to more than one KL-type (K2/K13, K3/KL116, K9/KL184, K11/KL181, K20/KL132, K21/KL163, K26/KL173, K30/K69, K46/KL146, K57/K68, K64/KL178, K74/K80, and KL110/KL116) likely by targeting the same glycosidic linkage in two structurally distinct polysaccharides (probably valid also for not yet characterized CPS compositions of KL > 100). There were four exceptional podovirus proteins: KP32gp37, KLEO13gp10, and KN1dep/Dp42 recognizing and cleaving panel of different CPS types: K3/KL110/KL116/KL117, K22/K37/KL111and KL101/KL120/KL170, respectively. Since the capsule tropism of KP32gp37and KLEO13gp10 partially overlapped with other enzymes, we described this phenomenon in a separate paragraph as case studies.

The structural and sequence variability within particular capsule tropisms versus enzyme origin was studied in groups encompassing several representatives. K1-specific depolymerases derived from six podoviruses (58-98 aa %identity) and one jumbo phage (32-35 aa %identity) formed a uniform K1 group 1 (TM score 0.9) with the same organization of domains, of which five podoviruses from the *Drulisvirus* genus shared >95 aa %identity in depolymerase sequence, although they originated from distinct geographic regions and hosts.

The most representative group was K2 depolymerases (13 enzymes), six of which were tested for the degradation of K13 CPS with the same core polysaccharide unit. Depolymerases from group 1 cleave α-D-Glc-(1 → 3)-β-D-Glc in K2/K13 CPS, whereas those from group 2 degrade β-D-Glc-(1 → 4)-β-D-Man in K2/K13 CPS^18,38,40^. Each group was structurally homogeneous with a high TM score (0.9–1.0), except for DpK2 enzyme (TM score > 0.85), although the aa %identity was > 97. The differences in TM score were verified in PyMOL, showing that subtle changes in amino acid content resulted in C-terminus modified spatial orientation. Overall, we might assume that all K2 group 1 enzymes can digest K13 CPS, even if not verified experimentally. K2 group 1 were almost identical in terms of amino acid sequence ( > 97 %identity), and all five were *Webervirus* (siphovirus) proteins originating from geographically distinct regions, which might suggest this group is exclusive to the siphovirus.

The K2 depolymerase group 2 was more diverse, encompassing five podovirus proteins, along with one protein derived from sipho-, myo-, and jumbo myovirus, showing the aa % identity at a low level (28%-55%). Similarly to K1 targeting depolymerases, the K2-specific enzymes from four *Drulisvirus*es were almost identical in sequence (96-98 aa %identity), despite the divergent origin of isolation and hosts. All analyzed depolymerases targeting K47 originated from podoviruses and formed two structural groups, exhibiting a high TM score and sequence similarity within the group (TM score >0.9). In turn, the P560dep with a different domain organization, previously assigned in the literature as K47-specific, targeted the KL169 strain. The K62 active enzymes formed two structure- and sequence-wise coherent groups (1 and 2), originating either from lytic *Webervirus* (siphovirus) or from prophages belonging to siphoviruses. These might represent another example of sipho-morphotype exclusive depolymerases. Seven enzymes specific to K64/KL178 capsules, originating from prophage, podo-, and jumbo phages, formed two groups (1 and 2) with different domain organizations, with a TM-score of 0.9 within each group, and high amino acid similarity (98%), but only within proteins derived from podoviruses (group 1). In some cases (K3, K14, K23, K28, K46, K62, K64/KL178, and KL111), both lytic and prophage-associated enzymes were found to target the same CPS, having (Tm score > 0.9; 29-99 aa% identity).

In summary, we found that phage-derived depolymerases display substantial sequence, structural, and functional diversity, with most well‑defined groups exhibiting distinct Klebsiella capsule tropism. Although most enzymes have narrow, single‑serotype specificity, several cross‑reactive depolymerases were found, as well as two structurally defined groups within the same K‑type. It highlights both conserved evolutionary patterns (*Webervirus, Drulisvirus*) and sequence versatility across depolymerases derived from phage lineages (podo-, myo-, sipho- and jumbo morphotypes).

### Structure prediction for depolymerase classification–DepoCatalog

To investigate whether there are any purpose-built motifs or domain architectures that correlate with the substrate specificity, we attempted to categorize depolymerases based on the overall protein structure model and domain dissection, omitting the N-terminus due to high conservation amongst the closely related phages and morphotypes, and lack of influence on enzymatic activity (Latka et al.^17^).

**Fig. 3:**
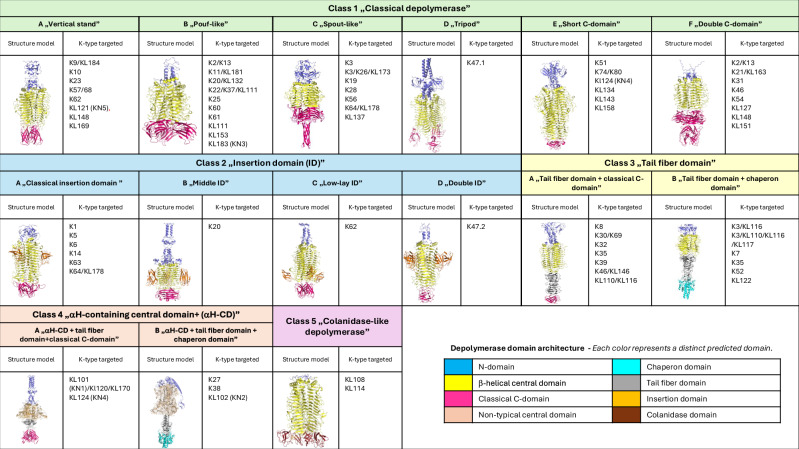
Structural classification of Klebsiella phage depolymerases based on overall architecture model (AlphaFold3.0), predicted domain dissection (PyMOL), and specificity toward K-type (spot test). Depolymerase representative model structures are grouped into five major classes and 14 subclasses. Protein color coding highlights specific domain types, enabling direct comparison of structural diversity across classes: N-domain (blue); β-helical central domain (yellow); classical C-domain (pink); non-typical central domain (salmon); chaperon domain (cyan); tail fiber domain (gray); insertion domain (orange); colanidase domain (brown).

Depolymerases were classified into five principal structural classes and fourteen subclasses based on their overall architectural features (Fig. 3). In Fig. 3, each subclass includes only one protein representative and a list of capsules targeted by proteins belonging to this subclass. A detailed graphical visualization of DepoCatalog with all classes, subclasses, protein names, targeted K-type, polysaccharide subunit composition (if available), and distinct structure models (except overlapping homologs) is presented in Supplementary Fig. 5.

**Class 1,** **‘classical depolymerase’**, is the most abundant, with 42 members. In this class, the β-helical central domain, located between cap 1 (the last α-helix preceding β-helix) and cap 2 (first α-helix or loop after β-helix), is composed of 10Latka20 rungs, which are followed by visually separated C-terminal(s). This class is subdivided into six subgroups (A-F) that can be distinguished depending on the C-terminal domain structure model. Class 1 A (19 members) is characterized by β-sheets in the C-terminal domain arranged perpendicularly to the β-helix, resembling a **‘vertical stand’**. Class 1B proteins have the β-sheets of the C-terminus parallel to those in the β-helix, resembling a sort of **‘pouf-like’** structure with 21 members. Among 10 depolymerases, a **‘spout-like’** shape could be noticed as an addition to parallel β-sheets of the C-terminal domain, forming Class 1 C. In one case of Class 1D (K47_Dep42 and K47_KLEO27gp6), the monomers of the C-terminal domain did not trimerize as a common structure model but as three separate units forming a kind of **‘tripod’** (pLDDT >90). Six depolymerases were grouped in Class 1E, having exceptionally long central domain (~20 rungs), followed by only a couple of β-strands-long, tapering **‘short C-domain’**. Finally, Class 1 F with **‘double C-domain’** was distinguished based on 19 representatives, also being the most diverse subgroup of classical depolymerases. K2/K13_B1dep has a C-domain composed of β-sheets parallel to the β-helix, followed by a needle, which is formed of α-helices. KP32gp38 (K21/KL163) and KLEO26gp187 (K31) possess two domains, the first with β-sheets parallel, the second perpendicular to β-helix, named in the published report as CBM-LEC duet^29,41^. The remaining representatives of Class 1 F possess two visually separate modules at the C-terminus with diverse shapes and folds. Some of the C-terminal domains of Class 1 A and 1 F were found to be homologous to carbohydrate-binding module (CBM) and/or lectin, however, with a low score in DALI.

**Class 2** was distinguished based on the presence of an additional predicted structure element, formed by antiparallel β-sheets reaching out of the central β-helix, called **‘insertion domain’**. Insertion domain varies in terms of location: Class 2 A - close to cap 1, **‘classical insertion domain’**; Class 2B - in the middle of the enzymatic domain, **‘middle insertion domain’** with one representative Kl-dep against K20; and Class 2 C - close to cap 2, **‘low-lay insertion domain’**. Proteins targeting K47 (Dpo43 and KLEO27gp6) possess two insertion domains present close to the cap 1 and ending with a short C-terminus, forming a Class 2D - **‘double insertion domain’**.

**Class 3,** **‘tail fiber domain’** has a short or middle-length central β-helix core and possesses a long β-strand-rich region, visually resembling a tail fiber (as also indicated by DALI search). These enzymes are divided into two subgroups. Class 3 A **‘tail fiber domain + classical C-domain’** and Class 3B **‘tail fiber domain + chaperon’** contained enzymes with an α-helix-rich domain with a predicted peptidase domain (pfam13884**)**.

**Class 4, ‘αH-containing central domain’** depolymerases feature classical β-helix supplemented with α-helices and loops inserted between the β-sheets of the different rungs, a morphology observed for five depolymerases. This class was further divided into two subgroups. In Class 4 A, the central domain is short (9-10 rungs), followed by a double C-domain with the first resembling a short fragment of tail fiber, and the second ‘vertical stand-like’. This kind of organization was presented by KN1 = KL101/KL120/KL170 and KN4 = KL124 (S1-2) specific enzymes, suggesting a unique polysaccharide subunit composition in CPS. In Class 4B, the central domain reassembles a duplication of the central domain from Class 4 A proteins in the form of a mirror image, which results in a very irregular shape. The C-terminus is also composed of two parts: a first longer tail fiber-like fragment, followed by an α-helix-rich chaperon with a predicted peptidase domain (pfam13884).

**Class 5** was formed for two representatives (Dep108.1 and KP24gp307) having **‘colanidase-like depolymerase’** domain with a long central domain and C-terminus, which is spread out.

### Capsular specificity shifts-extension via C-domain modification

**Fig. 4:**
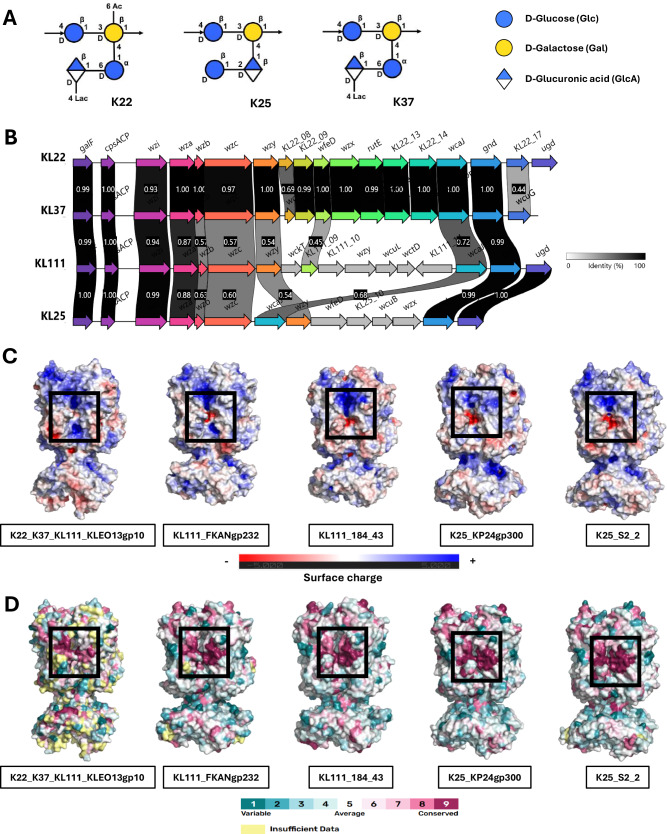
Comparative analysis of five selected phage depolymerases targeting K22/K37/KL111 and K25 capsule types. **A** Structural model representation of three *K. pneumoniae* capsule polysaccharide (CPS) subunits of K22, K25, and K37 serotypes. Each subunit is shown in a cartoon-style model. Images were sourced from the K-PAM database (https://iith.ac.in/K-PAM/home.html), a *Klebsiella* serotype predictor and surface antigen modeler; **B** Comparative genomic organization of KL22, KL25, KL37, and KL111 CPS loci. Gene clusters are displayed with annotated similarities, enabling visualization of conserved and variable regions across the loci (Clinker); **C** Surface electrostatic potential maps of analyzed depolymerases generated in PyMOL with the APBS plugin. Regions highlighted with black squares are putative locations of the active site, based on the most negative and positive charge accumulation and their location in the groove between monomers (details in MM section); **D** Amino acid conservation of the analysed depolymerases was made in ConSurf. Amino acid residues are colored according to conservation scores. Regions highlighted with black squares are possible locations of the active site, based on the grouping of highly conserved amino acid residues.

**Fig. 5:**
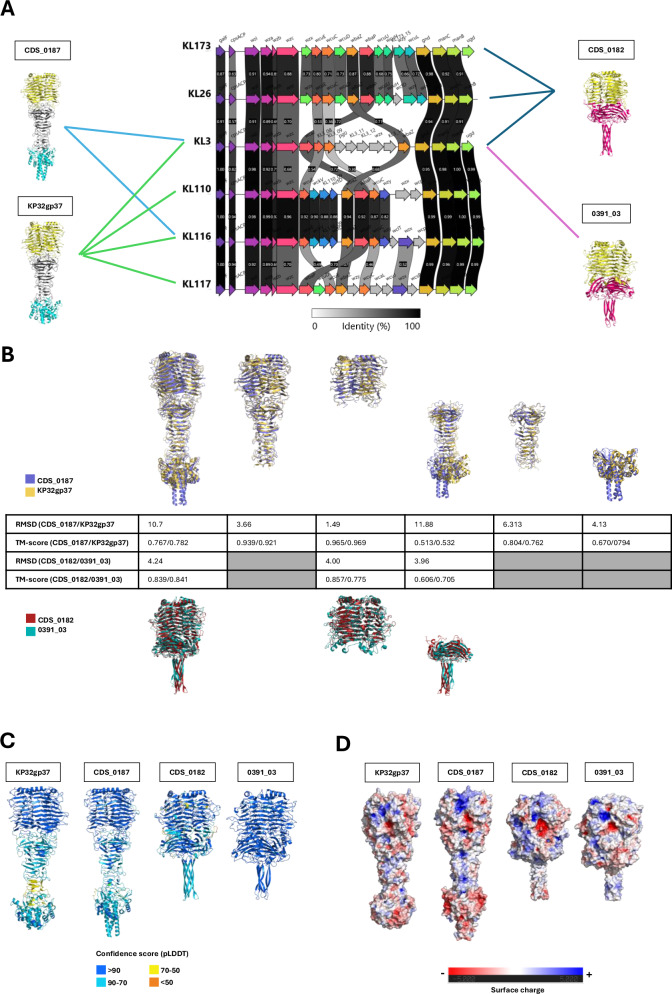
Comparative analysis of selected phage depolymerases targeting the K3 capsule type. **A** Structural models of CDS_0187 and KP32gp37, CDS_0182 and 0391_03 with corresponding comparative genomic organization of K3, K26, KL116, KL117, and KL173 CPS loci targeted by these enzymes. Gene clusters are displayed with annotated sequence and cluster organization similarities, enabling visualization of conserved and variable regions across the loci (Clinker); **B** Structural alignments of CDS_0187 versus KP32gp37 and CDS_0182 versus 0391_03 dissected in predicted domains (PyMOL), with associated root-mean-square deviation (RMSD) and Template Modeling (TM)-score values provided for each pairwise alignment; **C** Structural models predicted by AlphaFold3.0 with confidence of predictions indicated using pLDDT colour coding (>90, 90–70, 70–50, <50); **D** Surface electrostatic potential maps of analyzed depolymerases generated in PyMOL with the APBS plugin. Regions highlighted with accumulation of negative or positive charge indicate catalytic center location and sugar binding sites.

In this section, we aimed to explore the relationship between architectural similarities and the different substrate specificity of depolymerases, based on two cases: (a) proteins specific to KL111 and K25 (Fig. 4); and (b) proteins specific to K3 (Fig. 5) (Supplementary Fig. 2–3, 6–10, and Supplementary Data 3).

Despite being highly similar in structure prediction (TM score >0.9), five enzymes from Class 1B ‘pouf-like’ (KLEO13g10, 184_43, FKANgp232, KP24gp300, and S2-2) exhibited markedly different specificity profiles in experimental assays. While 184_43 (prophage) and FKANgp232 (jumbo virus) depolymerases were active against only a single KL111 capsule type, the KLEO13gp10 from podovirus could degrade up to three distinct polysaccharides (K22, K37, and KL111). The sugar subunit similarity between K22 and K37 types (differing only in galactose acetylation residue) might suggest the same degradation site; therefore, we hypothesize that the unknown KL111 CPS should be composed accordingly. We have also included two other proteins in the detailed analysis (KP24gp300 and S2-2), which are specific to the K25 serotype, differing from K22/K37 by the inverted position of two substituting sugars within the CPS repeating unit (Fig. 4A). We investigated whether amino acid composition or subtle structural differences, particularly within substrate-binding regions/catalytic pockets or domain interfaces, could clarify substrate versatility and potential capsule-specificity switch (K25 ↔ K111). Comparisons of the KL22, KL25, KL37, and KL111 CPS synthesis loci show differences in gene sequence and organization of the *cps* clusters (except for the KL22/KL37 pair) (Fig. 4B). Based on the 5 KL-loci comparison, it would not have been possible to predict the similarity in CPS composition among 22/37, 25, and 111 K types. Meaning, there is no obvious relation between enzyme specificity and the KL-locus genomic layout. The same pattern, the lack of similarities in Clinkier analysis, was also observed for the most pairs of compared CPS clusters for which the activity of a single enzyme was detected (K2/K13, K3/KL116, K9/KL184, K11/KL181, K20/KL132, K21/KL163, K26/KL173, K30/K69, K46/KL146, K57/K68, K64/KL178, K74/K80, and KL110/KL116) (Supplementary Fig. 11). These examples show that depolymerases may act on different CPS types that share key structural features, but *cps*‑locus similarity does not necessarily provide CPS structural similarity. Depolymerases recognize specific glycosidic linkages, and the entire CPS molecule should be accommodated in the sugar-binding protein groove, thus, only related CPS derivatives can serve as substrates for broad‑acting enzymes.

Sequence identity between KLEO13g10, 184_43, FKANgp232, KP24gp300, and S2-2 showed that the highest level of identity (55% and 42%) occurred between depolymerases degrading the same serotype (KL111 and K25). The most dissimilar was depolymerase KLEO13gp10 active against three serotypes (K22, K37, KL111), whose aa %identity to the other enzymes was only 28-32%. Detailed data are summarized in Supplementary Fig. 6. The three-dimensional structure models of all five depolymerases (excluding the N-terminal domain) were compared, indicating high structural similarity (TM-score 0.89−0.98) (Supplementary Fig. 7). Separate comparison for central domains displayed TM-score between 0.93 and 0.98, while for the C-terminal domains, the values were lower (0.74) for KLEOgp10 against others, 0.83-0.86 between K22 and K25 specific proteins, and up to 0.93-0.95 within the same specificity, indicating greater structural similarity in the central domain and more variability in the C-terminus (Supplementary Fig. 8). Analysis of surface electrostatic potentials (Fig. 4C) indicated a significant accumulation of negative and positive charge occurring in space between monomers in all analyzed depolymerases, suggesting that this region may harbor the active site (intersubunit position). However, in KLEO13gp10, there were fewer negatively charged amino acids in this region, but they were more concentrated on the solvent-exposed surface of a single monomer - also present in S2-2 depolymerase. Additionally, a distinct cluster of negatively charged residues was identified on the underside of the KLEO13gp10 C-terminal domain, which appeared weaker in other analyzed enzymes.

Analysis of amino acid residue conservation revealed the presence of highly conserved amino acid residues within the intermonomer region corresponding to the above-described putative active site location. Several conserved motifs were also identified in the intermonomer region, indicating a possible universal catalytic mechanism for those enzymes. In the C-terminal domain, conserved motifs were mainly observed in the depolymerases FKANgp232, 184_43, KP24gp300, and S2-2, whereas KLEO13gp10 displayed a different conservation pattern and didn’t share these motifs. Because the conservation profile of KLEO13gp10 was derived from a smaller number of homologous sequences (*n* = 9), the resolution of residue-specific conservation can be limited (Fig. 4D and Supplementary Fig. 9-10). In summary, we found that closely related enzymes are structurally identical in central domains but vary in the C-terminal shape and surface charge distribution. If the TM score for the C-terminus was > 0.9, the enzymes exhibited the same specificity. We propose that the C-terminal region influences the range of CPS structures that can be accommodated and represents the most rapidly diversifying domain, potentially enabling capsular specificity switching or expansion of substrate specificity.

The second case concerned K3-specific depolymerases (CDS_0187 and KP32gp37), which differed in their tropism toward further K-type capsules (KL110, KL116, and KL117) and protein pair (CDS_0182 and 0391_03), degrading the capsule of K3/K26/KL173 or only K3 assigned strains, respectively (Fig. 5, Supplementary Figs. 2–3 and Supplementary Data 3). The schematic alignment of CPS biosynthesis gene clusters of six capsule types (KL173, KL26, KL3, KL110, KL116, KL117) highlights high organization and sequence similarity between KL173/KL26 and KL110/KL116, suggesting a similar sugar bond in CPS recognized by each depolymerase. During the experiments, we found that the K3 serotype probably exists in two CPS sub-versions (named here as K3.1 and K3.2). The CDS_0182 and 0391_03 recognized and cleaved the capsule of K3.1 *K. pneumoniae* strains (CIP 52.146 and INF117.2), whereas CDS_0187 and KP32gp37 targeted K3.2 *K. pneumoniae* strain KP271 (Supplementary Data 2). The KP32 phage was able to propagate only on the K3.2 strain, but not on the K3.1 strains. In contrast, the jumbo phage KluzKp13 encoding CDS_0182 and CDS_0187 RBPs infected all three K3 strains (Supplementary Data 2). Moreover, the spot test on our full K-type collection revealed that the activity of prophage enzyme 0391_03 was narrowed only to K3.1 strains, in contrast to its homolog CDS_0182, which targeted K3.1/K26/KL173. The structural alignment of these ‘spout-like’ enzymes revealed slight differences for the full proteins (TM score 0.8), central domains (TM score 0.7–0.8), and C-terminus (TM score 0.6-0.7). Accordingly, the surface electrostatic potential maps also showed differences in the distribution of charged residues.

Similar analyses were done for the second pair of enzymes: CDS_0187 specific to K3.2/KL116 and KP32gp37 targeting K3.2/KL110/KL116/KL117. The structural similarities by superposition across their full-length proteins and their individual domains showed that both enzymes overlap closely, indicating a conserved three‑dimensional architecture of Class 3B ‘tail fiber domain + chaperon’, but the TM score for the full protein was relatively low (0.76-0.78). Similarly, the tail domain alone, tail domain+chaperon, and chaperon alone exhibit substantial divergence (TM score 0.5-0.8) with most regions showing high confidence ( > 70). The comparison of electrostatic surface potential demonstrated that, despite overall structural similarity, differences in surface charge distribution are evident. It might therefore explain differences in binding specificity toward distinct Klebsiella capsule types.

To summarize, a pair of enzymes with distant origins, CDS_0187 from jumbo phage versus KP32gp37 from podovirus, with low sequence similarity (45%, Supplementary Figs. 2–3), share a highly conserved central domain (TM score > 0.96), and subtly different C-terminal domain (tail fiber, TM score > 0.76), resulting in partially overlapping capsule type specificity. The second pair: CDS_0182 from jumbo phage and prophage 0391_03 displayed slightly dissimilar structure models, but also partially overlapping capsule specificity. Based on these two cases, we again suggest that the C-terminus may affect the range of accommodated CPS; therefore, it is the most rapidly diversifying domain. The structural comparison of two enzymes at the level of TM score 0.8-0.9 cannot exclude partially overlapping capsule type specificity.

### DepoCat: a web-based database

Based on the comprehensive dataset of experimentally characterized depolymerases and the findings described in this study, we designed the DepoCat database (https://depocat.uwr.edu.pl). DepoCat is an interactive database of depolymerases derived from Klebsiella bacteriophages. Currently, it includes 129 depolymerases. Each protein is assigned to one of five structural model classes and further categorized into specific structural subclasses (according to Fig. 3). For every protein, a detailed entry provides metadata, including basic characteristics, the source bacteriophage, and relevant references. Interactive 3D models are available for all proteins implemented by the model-viewer web component developed by Google^42^, allowing visualization of the structure model (AlphaFold3.0) for individual domains and providing access to confidence scores (pLDDT). The database enables users to examine proteins organized by structural classification or capsular KL-type specificity.

## Discussion

Phage depolymerases are typically highly specific, recognizing and degrading the capsular polysaccharides of a single serotype^20^. Exceptions are rare and usually limited to serotypes with closely related polysaccharide composition, such as K2/K13 and K30/K69, which share an identical sugar backbone, often resulting in cross-reactive enzymatic activity^43,44^. To date, 59 recombinant *Klebsiella* phage depolymerases have been reported before, targeting 32 distinct capsular types, with the most significant number of depolymerases reported for serotypes K1 and K2^17,45,46^.

In our study, 129 recombinant Klebsiella phage depolymerases were analyzed, of which 90 were prepared by our team and experimentally confirmed for their specificity in degrading *Klebsiella* CPS using a K1-KL186 *Klebsiella spp*. panel (with some exceptions). These enzymes originated from three distinct phage sources: prophages, jumbo phages, and common lytic phages. By analyzing the unique structural, functional, and phylogenetic diversity of depolymerases, we established a comprehensive catalog currently encompassing enzymes targeting 75 distinct capsule types. Based on the overall protein structure model and domain architecture, we proposed a systematic classification scheme comprising five principal classes and fourteen sub-classes. This framework provides clear insights into the remarkable evolutionary diversification of *Klebsiella* phage depolymerases, which appears to mirror the extensive heterogeneity of capsule types within the host species.

Most identified depolymerases are encoded by podoviruses with a simple RBP system, including *Przondovirus* with a dual-RBP system enabling, in principle, the recognition of two capsular types. Examples include Klebsiella phages K11 (recognizing K11 and K31), K5-4 (K5 and K8), KN3-1 (K56 and KN3), and KP32 (K3 and K21/KL163)^3,17,33,44,47^. Klebsiella podovirus vB_KpnP_IME205 encodes two depolymerases (Dpo42 and Dpo43) that both target the K47 capsular serotype but act on a different subset of strains, indicating subtle intra-serotype variations in this capsule composition^33^. Siphoviruses infecting *Klebsiella* display a simple RBP architecture, usually including a single RBP, whereas Klebsiella jumbo phages exhibit the most complex branching RBP architecture, often comprising multiple depolymerases and therefore have accordingly the broadest host ranges among all known phages targeting this species^20^. The most elaborate RBP system was described in the jumbo phage vB_KpM_FBKp24, with 14 distinct RBPs, 11 of which exhibit confirmed depolymerase activity (this study). Together, these enzymes enable recognition of 14 different capsular types — including three with dual activity (K2/13, K19, K25, K35, K46/KL146, K61, K64/KL178, KL114, KL134, KL137, KL158) highlighting the exceptional host range of this phage^21^ (this study).

Evolutionary processes vary widely depending on phage type, genome size, and ecological context^48^. It was also proposed that recombination events are common in phage evolution and contribute to the mosaic nature of RBP genes^49,50^. Indeed, within several capsule-specific groups (including K1, K2/K13 group2, K3, K5, K11, K14, K21, K23, K28, K30/K69, K35, K46, K54, K57/K68, K62, K64/KL178, and KL111), we identified depolymerases encoded by both lytic and prophage, or with a podo-, myo-/jumbo viruses, that share a conserved domain organization and almost identical structure prediction (TM score 0.9) despite notable differences in amino acid sequence identity (30-40 aa%). This pattern suggests that these enzymes first spread by horizontal transfer as paired-domains (central + C-terminus), followed by independent evolution across distinct taxonomic lineages, retaining a conserved spatial configuration and substrate specificity. Such structural and functional conservation across diverse taxa, coupled with sequence divergence, is consistent with a xenologous origin and points to strong purifying selection that preserves enzymatic architecture critical for capsule degradation, though the underlying evolutionary mechanisms remain to be formally investigated.

Significant differences for a given specificity were seen in the depolymerase sequence, but only across phage genera. In contrast, *Drulisvirus* depolymerases from K1 group 1 and K2 group 2 were conserved, even those derived from geographically distant sources and propagated in different bacterial hosts.

We also found an interesting case of taxa-specific enzymes in lytic phages and prophages. It was the siphovirus-exclusive depolymerases, five *Webervirus* proteins in K2 group 1 and four in K62 group 1 (at least one confirmed as a *Webervirus* protein). Of course, we cannot exclude the possibility that this conclusion is based solely on a few representative proteins described so far. As phages are systematically discovered, it may turn out that this group expands to include representatives from other taxa as well.

Moreover, based on the specificity profiles of enzymes originating from different phage morphotypes, we observed an interesting trend: depolymerases derived from podoviruses (KP32gp37, KLEO13gp10, and KN1dep/Dp42) exhibited a noticeably broader specificity spectrum compared with their counterparts encoded by jumbo phages. Therefore, we can propose a hypothesis that small phages, which typically encode no more than two RBPs, exhibit greater variability and flexibility in adapting to a wider range of capsular receptors by expanding the activity range of individual depolymerases. In contrast, jumbo phages appear to adopt the opposite strategy: they can accommodate multiple distinct RBPs (in some cases up to 15) in their larger genomes, each characterized by a narrower specificity spectrum. This hypothesis is based on a limited number of examples and would require systematic testing across a larger and unbiased collection of depolymerases to be validated. Dedicated comparative analyses across phage morphotypes are needed to assess whether this represents a general evolutionary strategy.

The ability to degrade multiple capsular types could be largely attributed to flexible or double substrate-binding regions (carbohydrate-binding motifs), which enable versatile interactions with structurally distinct bacterial polysaccharides. Broad active enzymes often contain adaptable catalytic domains that allow them to interact with diverse polysaccharides^51^. Our catalog shows double K-type tropism for CPS sharing the same sugar unit (K2/K13; K22/K37; K30/K69, K57/K68, and K74/K80), proving that enzymatic RBPs are substrate-specific. Nevertheless, we found four proteins, derived from podoviruses, that enabled targeting more than two KL types: KP32gp37 (K3/KL110/KL116/KL117), KLEO13gp10 (K22/K37/KL111), and KN1dep/Dp42 degrading KL101/KL120/KL170. Based on the *cps*-cluster sequence comparison, we might assume that K21/KL163, K26/KL173, K64/KL178, KL110/KL116, and KL101/KL120/KL170 could be similar in polysaccharide composition within pairing. Although we observed the extended specificity range phenomenon (K3, K25, and KL111 cases), no information on the corresponding CPS composition is currently available.

The structure prediction analyses detected subtle spatial variations, primarily localized within the C-terminus and conformational differences, that appear to facilitate recognition of distinct glycosidic linkages and sugar residues. These features contribute to the broad specificity and functional versatility observed among depolymerases encountered among members of the same structural class. Therefore, as long as the database of proteins with experimentally confirmed specificity remains limited, the prediction of substrate specificity is uncertain unless sequence identity to a characterized enzyme is exceptionally high. Machine learning tools and bioinformatics pipelines could improve predictive accuracy^35,36,52^, but these models require larger datasets of characterized enzymes to be truly effective. Hybrid approaches combining sequence, structure modelling, and biochemical data offer the most promise.

In this study, we explored structural evidence bearing on the relative contributions of domain shuffling and point mutations to depolymerase diversity^51^, although there is a hypothesis that domain shuffling plays a more prominent role in generating functional variants^53^. Domain shuffling between the N-terminus and the Central+C-terminal parts can result in the acquisition of external enzymes with specific properties, potentially affecting the host range^17,41,54,55^. But major uncertainty is whether evolution enables rapid adaptation to other capsule types by separate enzymatic or C-terminus domain exchange. In contrast to recombination events, point mutations serve to fine-tune enzymatic properties within existing domain frameworks. Mutations in key residues can modulate activity, stability, and specificity, thereby affecting phage infectivity. This is what we found in K22/K37/KL111 and K25 targeting depolymerases, as well as in K3-specific enzymes. Almost identical structural model of the central enzymatic domain and conserved catalytic pocket, but a different C-terminus and distribution of negatively charged residues resulted in a sugar recognition difference. We showed that the C-terminus may affect the range of accommodated CPS, providing either the capsular specificity switch or partially overlapping capsule-type activity. Collectively, these observations suggest that the C-terminal domain is a primary locus of structural variation associated with differences in capsular specificity, and may represent a key interface through which depolymerase host range diversifies over evolutionary time.

Due to the high specificity, depolymerases also hold potential for diagnostic use, enabling the rapid serotyping of bacterial isolates^16,47,56,57^. Their serotype‑level specificity functions as a biochemical barcode in diagnostics, enabling rapid strain identification and improving antigen exposure for downstream assays. Also, they have proved successful in therapeutic contexts by degrading biofilms, sensitizing bacteria to capsule-independent phages, and increasing bacteria sensitization to capsule-independent phages, and to increase bacterial susceptibility to immune recognition and clearance upon capsule degradation^2,3,6,58–60^. Depolymerases generate defined capsular fragments during cleavage that can serve as immunogenic substrates for vaccine development or serological assays^61^. The ability of depolymerases to enhance immune recognition and antibiotic access makes them effective adjuncts to existing therapies rather than stand‑alone antimicrobials.

To date, in vivo evaluation of depolymerase efficacy has been limited to animal studies^58,62–65^. These investigations consistently show that phage‑derived depolymerases can reduce bacterial virulence, enhance innate immune clearance, reduce infection severity, and improve survival across multiple infection models. When administered at appropriate doses, depolymerases were both safe and effective as in vivo antimicrobials. Notably, mice treated with depolymerase exhibited higher survival rates than those receiving whole phage, even when the target strain did not support phage propagation^63^. However, the narrow serotype‑specific recognition typical of natural depolymerases constrains their broader clinical applicability, underscoring the need for engineered variants with expanded substrate scope. Emerging protein‑engineering approaches offer a promising route to expand the substrate range of depolymerases, enabling the development of more versatile enzymes for therapeutic use and providing a potential strategy for combating multidrug‑resistant bacterial infections.

The design of synthetic enzymes is possible through modular protein engineering, but the engineering of depolymerases requires structural insights and modeling^17,41,66,67^. Genetic miniaturization has also proved feasible for a truncated KP34gp57 variant active as a monomeric mini‑enzyme^31,41^. Nevertheless, the rational design for translational applications requires broad experimental datasets and AI‑supported analyses to uncover generalizable design rules.

Despite its scale, the current catalog has inherent limitations that are important to acknowledge. With respect to capsular coverage, the dataset spans 75 KL-types out of the ~163 currently recognized loci, because of the limited number of experimentally confirmed specificities of recombinantly prepared enzymes to date. This depolymerase collection was assembled from a curated set of sources and was not designed as an exhaustive survey of all publicly available Klebsiella phage genomic data. Finally, the observation that multiple structurally distinct depolymerases can target the same KL-type suggests that the enzymatic repertoire against any given polysaccharide is unlikely to be fully captured by the current dataset, and that many depolymerases remain to be discovered. Collectively, these considerations indicate that DepoCatalog, while by far the most comprehensive collection of *Klebsiella* phage depolymerases assembled to date, represents a foundation rather than a definitive inventory, and that continued systematic exploration of phage-derived depolymerases across the full KL-type spectrum will substantially expand this resource.

## Methods

### In silico identification of depolymerase

Our search was based on the previous assumption and discoveries of RBPs with depolymerase activity showing specific features (size, annotation, domain homology, predicted β-helical structure, lyase topology)^20^. Enzymes lacking these annotations or possessing atypical domain architectures not recognized by the homology-based tools employed may not be captured by this approach.

The following criteria were used for putative depolymerase identification: (1) the protein must be longer than 500 amino acid residues; (2) the protein must be annotated as tail fiber/tail spike protein in the searched database; (3) the protein must show homology to domains annotated as lyases (e.g. hyaluronate lyases (hyaluronidases), pectin/pectate lyases, alginate lyases, K5 lyases) or hydrolases (e.g. sialidases, rhamnosialidases, levanases, dextranases or xylanases), recognized by BlastP (v2.16.1+)^68^, HMMER v3.4^69^ or HHpred Version: 57c8707149031cc9f8edceba362c71a3762bdbf8^70^; (4) a typical β-helical structure^20^ should be predicted by AlphaFold3.0^71^. BlastP was performed against the non-redundant protein sequences (nr) database using standard parameters (expect threshold: 0.05; word size: 5; matrix: BLOSUM62; gap costs: existence 11, extension 1; conditional composition score matrix adjustment). HMMER was used in quick search mode against Reference Proteomes with significance e-values: sequence 0.01 and hit 0.03. HHpred homology detection prediction was run using the PDB_mmCIF70 database and the following parameters (MSA generation method: HHblits ≥ UniRef30; maximum number of MSA generation iterations: 3; *E*-value cutoff for MSA generation: 1e-3; minimal sequence identity of MSA hits with query (%): 0; minimal coverage of MSA hits (%): 20; secondary structure scoring: during alignment; Alignment Mode: Realign with MAC: local:norealign; MAC realignment threshold: 0.3; number of target sequences: 250; minimal probability in hitlist (%): 20). Protein clustering was performed by BlastP with 80% sequence coverage and 50% sequence identity^68^. Structural predictions were obtained using AlphaFold3.0^71^, followed by visualization in PyMOL (Schrödinger, LLC, v3.0). The depolymerases derived from the prophages were identified in the KASPAH and KLEBPAVIA *Klebsiella spp*. genome collections in our previous study^39^. Prophage genome detection was performed using VirSorter2 v.1.0.1^72^ and PhySpy v5.0.10^73^. BlastN (v2.16.1+)^68^ was used for genome clustering with 90% sequence coverage and 50% sequence identity. Gene functional annotation was executed (30% sequence coverage and 95% sequence identity) with COG_KOG (2025)^74^, PDB (2025)^75^, PFAM v37^76^, PHROGs v4^77^, and UNICLUST (2025)^78^ databases. The morphotypes of prophages were assigned using the VIRFAM (2025) tool^79^. The comprehensive characteristics of Klebsiella phage depolymerases analyzed in this study are listed in Supplementary Data 3.

### Bacterial strains

*Klebsiella spp*. K-type panel used in this study originate from the Collection de l’Institut Pasteur (CIP), Paris, France; The National Collection of Type Cultures (NCTC), the UK Health Security Agency (UKHSA); collection of the Department of Pathogen Biology and Immunology, Wroclaw, Poland (GenBank, BioProject PRJNA1424276); the *Klebsiella* Acquisition Surveillance Project at Alfred Health (KASPAH) (Melbourne, Australia)^80–82^ provided by Kath Holt the London School of Hygiene and Tropical Medicine (LSHTM) Department of Infection Biology, London, UK; and the collection of the Department Biological Safety, German Federal Institute for Risk Assessment, Berlin, Germany (GenBank, BioProject PRJNA1424276). *Klebsiella spp*. KL-type panel used in this study encompasses 171 strains representing 146 distinct KL-types (Supplementary Data 1). All bacteria were cultured in Tryptone Soya Broth or Agar (TSB or TSA, Oxoid, Thermo Fisher Scientific, Waltham, MA, USA) at 37 °C and stored at −70 °C in a 20% glycerol-supplemented Trypticase Soy Broth (TSB, Becton Dickinson, and Company, Cockeysville, MD, USA).

The *Escherichia coli* strains (Thermo Fisher Scientific, Waltham, MA, USA) used for recombinant protein production: One Shot^TM^ TOP10 for plasmid propagation and One Shot^TM^ BL21 Star^TM^ (DE3) for recombinant protein expression, were cultured in LB broth, Miller (Luria-Bertani) (Difco, Becton, Dickinson and Company (BD), Franklin Lakes, NJ, USA) at 37 °C supplemented with 100 µg/mL ampicillin (IBI Scientific, Dubuque, IA, USA).

### Cloning, expression, and purification

The recombinant depolymerases were obtained through two different cloning approaches. A subset of genes was amplified in a PCR reaction using specific primers (Genomed, Warsaw, Poland) and high-fidelity polymerase, and molecularly cloned in-house using the Champion pET101 or pEXP5-NT/TOPO or pEXP5-CT/TOPO expression vectors (Thermo Fisher Scientific, Waltham, MA, USA) with a C- or N-terminal His tag (6x) and propagated in *E. coli* One Shot™ TOP10 strain (Supplementary Data 5). The accuracy of all clones was verified by sequencing (Genomed, Warsaw, Poland) using the specific primers (Supplementary Data 5). The remaining constructs were synthesized and prepared in expression vectors by commercial service GeneUniversal (Newark, DE, USA) or GenScript Biotech (Rijswijk, Netherlands) (Supplementary Data 5). Following the transformation into *E. coli* One Shot™ BL21 Star™ (DE3) or E. coli BL21 (DE3), vector-bearing cells were cultured in 500 mL of LB broth supplemented with 100 µg/mL ampicillin or 50 µg/mL kanamycin at 37 °C with agitation until the optical density reached 0.5–1.0 (OD_600nm_). Recombinant protein expression was induced at 16–20 °C for 16 h by adding isopropyl-β-D-thiogalactopyranoside (IPTG) to a final concentration of 0.2–1 mM. The cultures were then harvested by centrifugation (8000 × *g*, 15 min, 4 °C), and the resulting pellets were resuspended in lysis buffer (500 mM NaCl, 20 mM NaH₂PO₄, pH 7.4). The exact expression conditions for each protein are provided in Supplementary Data 5. After three freeze/thaw cycles and sonication, the lysates were centrifuged (16,000 × *g*, 30 min, 4 °C), and the supernatants were collected and filtered through a 0.22 µm filter (Sarstedt AG & Co., Numbrecht, Germany). His-tagged proteins were purified using Bio-Scale Mini Profinity IMAC cartridges (Bio-Rad, Hercules, CA, USA) or HisTrap HP 1 mL columns (Cytiva, Marlborough, MA, USA) in combination with an FPLC system (NGC Bio-Rad, Hercules, CA, USA or ÄKTA Pure Cytiva, Marlborough, MA, USA). The column was equilibrated with lysis buffer (500 mM NaCl, 20 mM NaH₂PO₄, pH 7.4), followed by sample loading and washing with 32 column volumes of the same buffer. The proteins were subsequently eluted using an elution buffer (500 mM NaCl, 20 mM NaH₂PO₄, 500 mM imidazole, pH 7.4). Alternatively, depolymerases were purified using cobalt‑NTA magnetic beads (Dynabeads His‑Tag Isolation and Pulldown, Thermo Fisher Scientific) according to the manufacturer’s instructions. Some recombinant proteins derived from Klebsiella prophages were prepared by a commercial company (GenScript Biotech, Rijswijk, Netherlands) with the protocol details provided in Supplementary Data 5. Finally, protein concentrations were determined spectrophotometrically (NanoPhotometer NP80, Implen GmbH, Schatzbogen, München, Germany) using a molar extinction coefficient calculated for each protein with the ProtParam tool^83^. All proteins were produced and tested as full‑length versions.

### SDS-PAGE protein analysis

Sodium dodecyl sulfate-polyacrylamide gel electrophoresis (SDS-PAGE) was conducted following the method of Laemmli^84^ using a 12% polyacrylamide gel. Next, the protein samples were mixed with Laemmli buffer (Bio-Rad, Hercules, CA, USA) in a 3:1 ratio, respectively, and analyzed in SDS-PAGE. The protein samples were heated at 99 °C for 7 min. Molecular weight standard Precision Plus Protein All Blue (10–250 kDa) (Bio-Rad, Hercules, CA, USA) was used. The protein bands were visualized by GelCode Blue staining (Thermo Fisher Scientific, Waltham, MA, USA). Representative SDS–PAGE images and bacterial lawn halo formation assays for each produced protein are available in the Zenodo repository (10.5281/zenodo.19927711). Notably, clear halo formation was consistently observed even when purified proteins were present at very low levels and not prominently visible on SDS–PAGE, reflecting the high catalytic efficiency of these enzymes.

### Depolymerase activity assay

Detection of enzyme activity was performed by spot assay on the *K. pneumoniae* KL-type panel listed in Supplementary Data 1. Bacteria were grown to an optical density (OD_600nm_) of 1.0, and then,1 ml of bacterial culture was directly transferred onto TSA plates. After drying, 10 µL of a serial two-fold dilution of recombinant enzyme in phosphate-buffered saline (PBS; 137 mM NaCl, 2.7 mM KCl, 10 mM Na_2_HPO_4_, 1.8 mM KH_2_PO_4_, pH 7.4). 10 µL of PBS buffer (negative control) was applied to the bacterial lawn in spots. The corresponding phage filtrate (10^7^–10^8^ PFU/mL) was spotted accordingly as the control of protein activity. Following an overnight incubation at 37 °C and an additional 24 h incubation at room temperature, plates were examined for the presence of halo zones (enzymes) or, in the case of phage activity, clear plaques surrounded by haloes, areas in which phage depolymerase degraded the CPS from the host strain (Supplementary Data 2). Representative images illustrating phage activity with halo formation, as well as recombinant enzyme halo visualization, are presented in Supplementary Fig. 12. All tests for depolymerases and phages from our collection were conducted with at least a biological triplicate. 18 unique literature-reported depolymerases were screened at least twice on the whole KL-type collection, and the positive results were repeated in triplicate. Note: our assays qualitatively assessed depolymerase activity (presence vs. absence), rather than enabling strict quantitative comparisons.

### The structural classification of depolymerases

The structural classification of full-length depolymerases was established by in silico delineating three distinct domains: the N-terminal domain, the central β-helical enzymatic domain, and the C-terminal domain^20^. Structural models were generated as homotrimers using AlphaFold3.0^71^ and subsequently visualized with PyMOL (The PyMOL Molecular Graphics System, Version 3.0, Schrödinger, LLC).

Domain boundaries were assigned based on the analysis of secondary structural elements within the predicted structures, following the classification criteria established by Huang et al.^85^ (Supplementary Fig. 1). The N-terminal domain was defined as the region preceding the terminal α-helix (cap 1), which serves as the structural demarcation between this domain and the central enzymatic domain. The central β-helical enzymatic domain was identified as the region initiating from cap 1 and adopting a right-handed parallel β-helix fold, a characteristic structural motif of depolymerases. In cases where an insertion domain was present within the β-helical core, it was distinguished by the occurrence of antiparallel β-sheets and α-helices forming an extended protruding loop. The termination of the central domain was defined by the presence of cap 2, the final α-helix of the β-helical fold. In the absence of cap 2, the domain boundary was delineated at the loop structure preceding the C-terminal β-sandwich domain. The C-terminal domain was assigned to the region downstream of cap 2 (or the corresponding loop structure in its absence) and was characterized by the presence of a β-sandwich fold. In some cases, more than one C-terminal domain could be distinguished per protein: (a) both adapting β-sandwich folds separated by loops or linkers; (b) the ultimate C-terminal β-sandwich was preceded by an elongated β-strand-rich motif, not forming a right-handed parallel β-helix fold, which was treated as a separate domain. In other cases, ultimate C-terminal domain was rich in alpha helices, forming a visually separated domain.

As an attempt to assign additional functions, C-terminal domain(s) or atypical parts of β-helix were selected from the 3D structure model and analyzed with the DALI v5 (2025) server^86^ using PDB search for domains previously detected as tailspikes. In particular, ‘CBM’ (carbohydrate-binding module), ‘LEC’ (lectin-like domain), ‘neuraminidase’, and ‘colanidase’ were of interest. Additionally, some regions between the β-helix and the C-terminus were found to be similar to the ‘tail fiber protein’. If at least the Z-score was > 2, the DALI hit was reported (however, since RMSD was not always below 3.0 Å, the results are not reported in detail). Proteins whose C-terminus was rich in alpha-helices were analyzed with PHYRE2.2^87^ in search of a C-terminal ‘chaperon’. The delineation was made based on BlastP (v2.16.1 + ) analysis run with standard parameters (as listed above), where the ‘peptidase’ was detected and treated as a ‘chaperon’ start.

Structural confidence scores were calculated for all enzymes based on homotrimer structure predictions obtained in AlphaFold3.0^71^. Global model quality measures (pTM, ipTM) and predicted distance error (PAE) statistics were extracted from the JSON files, while pLDDT values stored in the B-factor field for Cα atoms were read from the PDB files in order to calculate average pLDDT values for the entire structure model as well as averages for individual domains (N-terminal, Central, and C-terminal) based on previously defined ranges of amino acid residues. The analyses were performed using Python v3.14 custom scripts, and the results were presented in tabular form (Supplementary Data 4 and 6).

### Sequence and structural model alignment of proteins

The N-terminal domains were removed from full-length protein amino acid sequences, and BLASTP (v2.16.1+)^68^ with default settings was used to compare the resulting truncated sequences. For comparative analysis of CPS loci across different genomes, we used Clinker (v0.0.31)^88^, which enabled rapid visualization of gene clusters and their synteny. Protein structure models were compared using US-align (v2024.07.30)^89^, and all structural alignments and visualizations were carried out in PyMOL (v3.0; Schrödinger, LLC, 2010). We considered only the modelled central and C-term domain structures with pLDDT > 70 obtained with the truncation of the N-terminus from the full homotrimer in PyMOL. We also used PyMOL, along with the APBS plugin (v3.4.1), to calculate and display electrostatic surface potentials. Default PDB2PQR preparation was used to add hydrogens and assign partial charges and atomic radii. Electrostatic potential maps were calculated using APBS with a grid spacing of 0,50 Å. Molecular surfaces were then visualized in PyMOL using a color range of ±5.00 kT/e. To examine evolutionary conservation of amino acid residues, we used the ConSurf webserver (v2025^90–93^. Three-dimensional depolymerase structure predictions in PDB format were submitted, and conservation scores were calculated using default parameters. The server automatically retrieved homologous sequences, generated multiple sequence alignment, and computed Bayesian residue-specific conservation scores. These scores were then mapped onto the three-dimensional structure model in PyMOL to highlight regions likely to be functionally important. AlphaFold3.0 models with pLDDT score overlaid were prepared in PyMOL using AF3 pLDDT coloring scheme with custom code (provided in Supplementary Data 6). Custom scripts and DepoCat database code were developed with the assistance of AI-based tools (ChatGPT, OpenAI)^94^. All codes were reviewed, tested, and validated by the authors.

### Reporting summary

Further information on research design is available in the Nature Portfolio Reporting Summary linked to this article.

## Supplementary information

Supplementary Information

Description of Additional Supplementary Files

Supplementary Dataset 1

Supplementary Dataset 2

Supplementary Dataset 3

Supplementary Dataset 4

Supplementary Dataset 5

Supplementary Dataset 6

Reporting Summary

Transparent Peer Review file

## Data Availability

The data generated in this study are presented in the Supplementary Information and Supplementary Data 1–6. The GenBank accession numbers are listed in Supplementary Data 3 and in the Methods section. The depolymerase structure models in PDB formats generated using AlphaFold3.0 and depolymerase characteristic metadata are available at DepoCat (https://depocat.uwr.edu.pl/). Source data associated with this paper are available at Zenodo 10.5281/zenodo.19927711.

## Code availability

All custom codes have been deposited at Zenodo and are publicly available at 10.5281/zenodo.19927711.
