# Supplementary material for: DepoCatalog: mapping diversity of 129 recombinantly produced Klebsiella phage depolymerases
Source: Nat Commun. 2026 May 22;17:6724. doi: 10.1038/s41467-026-73570-7 (PMC13385381; doi:10.1038/s41467-026-73570-7)
Supplement: Supplementary file 1 — Supplementary Information [file 41467_2026_73570_MOESM1_ESM.pdf]

DepoCatalog: mapping diversity of 129 recombinantly expressed *Klebsiella* phage depolymerases

SUPPLEMENTARY INFORMATION

SUPPLEMENTARY INFORMATION

Supplementary Figure 1. Depolymerase domain dissection approach and its legend used in the Supplementary Tables. Symbolic representation of monosaccharide units used in capsular polysaccharide (CPS) structure diagrams. CPS structures were obtained from the K-PAM resource (K-PAM: Klebsiella Pneumoniae Antigen Typing Resource – K Antigen. Available at: https://iith.ac.in/K-PAM/k_antigen.html (Accessed: 15 March 2026)[1].

Supplementary Figure 2. Depolymerases are classified according to the capsular types (K/KL) of *K. pneumoniae* that they recognize. Within each type group, proteins are further subdivided into structural subgroups when distinct structural models are present. The table provides the protein name, assigned structural model, protein length (amino acids), and the proteins within each group. Representative monomeric structure models predicted using AlphaFold3.0 (based on homotrimer prediction) is shown for each protein. For depolymerases with experimentally characterized cleavage sites and catalytic residues, the positions of glycosidic bond cleavage and amino acids forming the active site are indicated. The names of proteins produced and experimentally tested in this study are highlighted in bold.

Supplementary Figure 3. Structural similarity between depolymerases is presented as heatmaps based on pairwise amino acid sequence similarity and TM-score values calculated for the central β-helix and C-terminal domains or for the central domain alone. Selected structural superpositions illustrate conserved architecture within compared proteins.

Supplementary Figure 4. Schematic comparison of the glycan structures of several K-antigens recognized in pairs by some of the depolymerases grouped into: **A**. glucose as the first monomer category, **B**. galactose as the first monomer category. The diagrams illustrate the monosaccharide composition, linkage positions, and overall structural organization of each antigen, including branching patterns and ring types. CPS structures were obtained from the K-PAM resource (*K-PAM: Klebsiella Pneumoniae Antigen Typing Resource – K Antigen*. Available at: https://iith.ac.in/K-PAM/k_antigen.html (Accessed: 15 March 2026)[1].

Supplementary Figure 5. Depolymerase catalog presented as a comparison of representative depolymerases from distinct classes and subclasses. The tables summarize subclass distinctions, targeted K-types, polysaccharide subunit compositions, and protein lengths for each enzyme. Predicted 3D protein structures generated with AlphaFold3.0 are shown below each entry, illustrating domain architecture and overall structural differences across subclasses.

Supplementary Figure 6. Pairwise amino acid sequence comparison of five *Klebsiella pneumoniae* CPS-degrading depolymerases using BLASTP. Each panel (A-E) displays an alignment of one query protein against remaining four.

Supplementary Figure 7. Structural comparison of five *K. pneumoniae* CPS- degrading depolymerases (excluding N-terminal domains). (A) Heatmap of pairwise structural similarity between depolymerases, calculated using USalign. Color intensity corresponds to TM- scores, with higher values indicating greater structural similarity. (B) Structural alignments of protein pairs visualised in PyMOL. Each alignment includes root-mean-square-deviation (RMSD) and TM- score values to quantify structural congruence.

Supplementary Figure 8. Domain-specific structural comparison of *K. pneumoniae* CPS- degrading depolymerases. (A) Heatmap of pairwise structural similarity between the central domains of depolymerases, computed using USalign. TM-scores are color-coded, with higher values indicating greater structural similarity. (B) Heatmap of C-terminal domains comparisons, also based on TM-scores calculated using USalign. (C) Structural alignments of the central domains visualized in PyMOL, with associated root-mean-square deviation (RMSD) and TM-score values provided for each pairwise alignment. (D) Structural alignments of the C-terminal domains, also shown with root-mean-square deviations (RMSD) and TM-scores.

Supplementary Figure 9. Conservation-based analysis of *K. pneumoniae* CPS- degrading depolymerases. Multiple sequence alignments (MSA) of five depolymerases, with amino acid conservation colouring according to ConSurf scores. Conservation level range from variable (low scores) to highly conserved (high scores), visually emphasizing conserved motifs. Residues corresponding to the predicted active site regions marked with black rectangles, highlighting their conservation across sequences and spatial co- localization in the structures. This representation provides a sequence-level view of conserved, functionally relevant regions across the protein set.

Supplementary Figure 10. Conservation-based analysis of *K. pneumoniae* CPS-degrading depolymerases. Each panel displays amino acid sequence of depolymerase, colored according to residue conservation scores from ConSurf analysis. Below each amino acid, additional annotations indicate whether the residue is exposed or buried, or is classified as functionally, or structurally important. Residues corresponding to regions predicted as possible active site in Figure 4 are highlighted by black rectangles.

Supplementary Figure 11. Comparative genomic organization of selected CPS loci. Gene clusters are displayed with annotated sequence and cluster organisation similarities, enabling visualization of conserved and variable regions across the loci (Clinker)

Supplementary Figure 12. Representative images illustrating phage activity with halo formation, as well as recombinant enzyme halo visualization

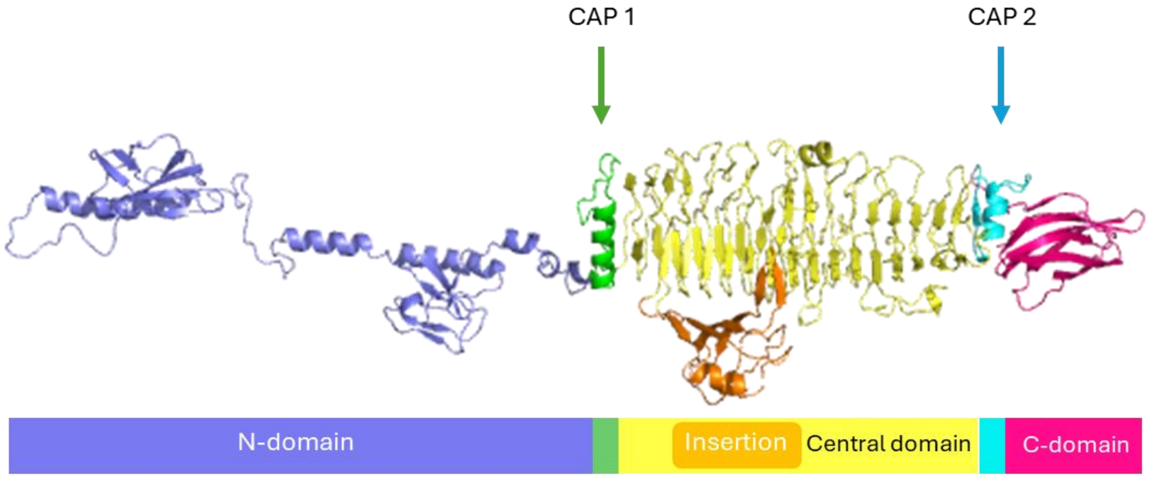

Identification and delineation of depolymerase domains based on Huang et al. 2024[2].

	N-domain
	Beta-helical central domain
	Non-typical central domain
	C-domain (including i.e. CBM, LEC domains)
	Insertion domain
	Chaperon domain
	Tail fiber domain
	Colanidase domain

Domain architecture legend used in this study

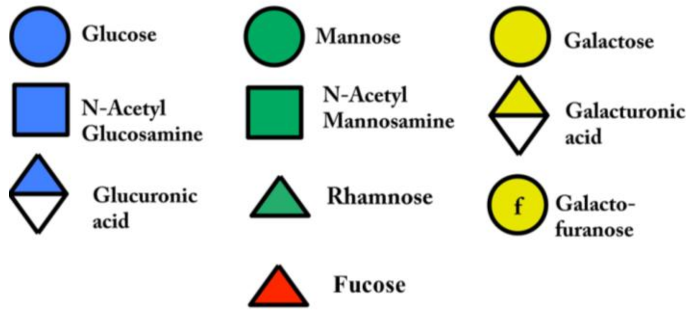

Symbolic representation of monosaccharide units used in polysaccharide structure diagrams

Supplementary Figure 1. Depolymerase domain dissection approach and its legend used in the Supplementary Tables. Symbolic representation of monosaccharide units used in capsular polysaccharide (CPS) structure diagrams. CPS structures were obtained from the K-PAM resource (*K-PAM: Klebsiella Pneumoniae Antigen Typing Resource – K Antigen*. Available at: https://iith.ac.in/K-PAM/k_antigen.html (Accessed: 15 March 2026)[1].

Supplementary Figure 2. Depolymerases are **classified according to the capsular types (K/KL)** of *K. pneumoniae* that they recognize. Within each type group, proteins are further subdivided into structural subgroups when distinct structural models are present. The table provides the protein name, assigned structural model, protein length (amino acids), and the proteins within each group. Representative **monomeric structure models predicted using AlphaFold3.0** (based on homotrimer prediction) is shown for each protein. For depolymerases with experimentally characterized **cleavage sites and catalytic residues**, the positions of glycosidic bond cleavage and amino acids forming the active site are indicated. The names of **proteins produced and experimentally tested in this study are highlighted in bold**.

Supplementary Figure 2. continued

Cleavage site
K1-ORF34 [3]

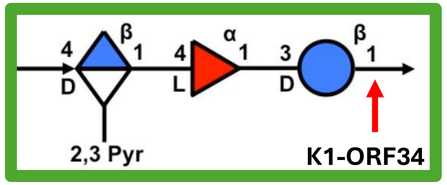

Active Site Residues **K1-ORF34**
Tyr311, His 373, and Arg397[3]

**K-type target**	**K1**						
**Group**	Group 1						
**Protein name**	S2-4	GBH001_056	K1_ORF34	**gp47**	**KLEO1gp03**	Kpv71_52	**gp09**
**Length**	888 aa	651 aa	651 aa	651 aa	651 aa	651 aa	644 aa
**No. of proteins in the group**	7 proteins						
**Predicted protein structure (AlphaFold3)**	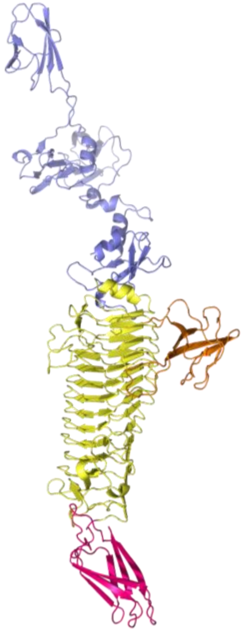	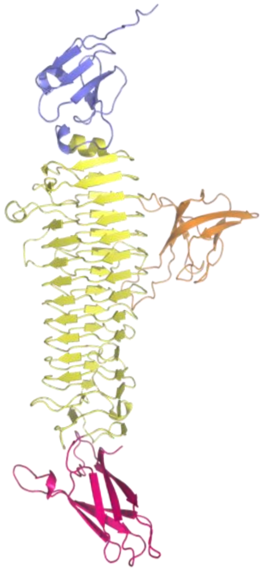	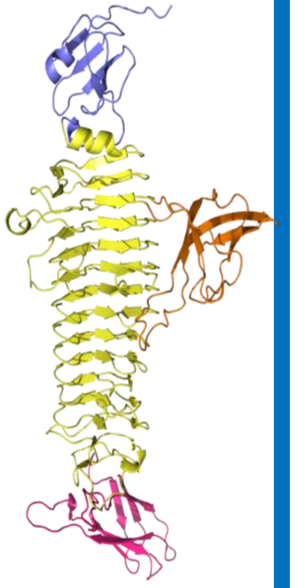	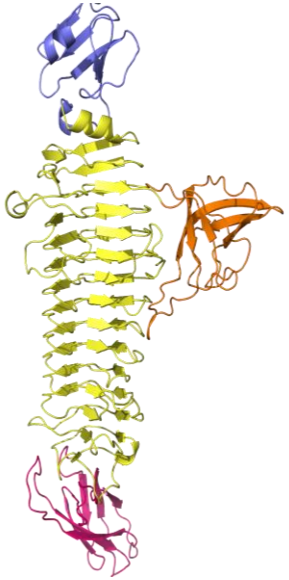	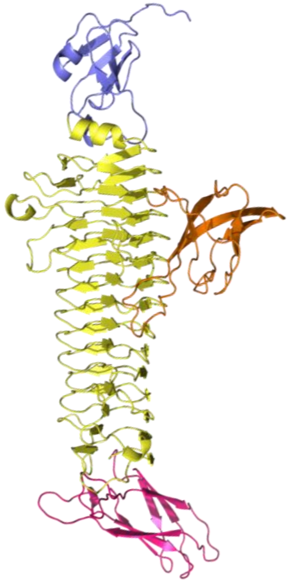	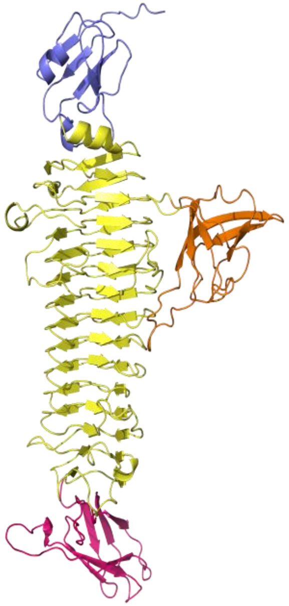	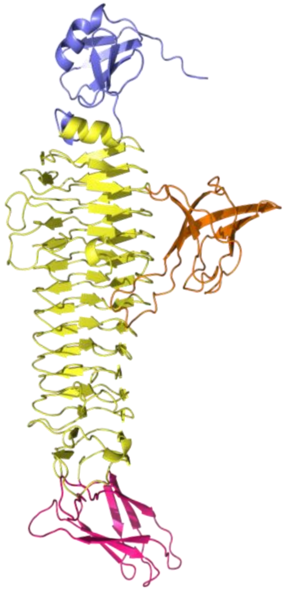

Supplementary Figure 2. continued

**K-type target**	**K2**	
**Group**	Group 1	Group 2
**Protein name**	DpK2	**KP24gp196**
**Length**	907 aa	660 aa
**No. of proteins in the group**	5 proteins	8 proteins
**Predicted protein structure (AlphaFold3)**	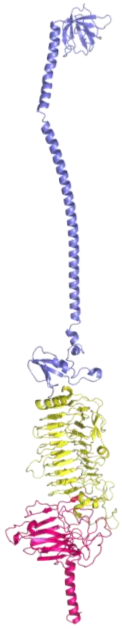	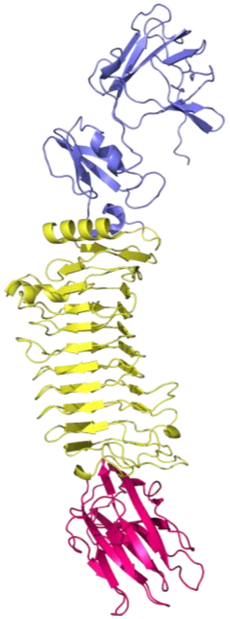

Cleavage sites
K2 depolymerases [4–6]

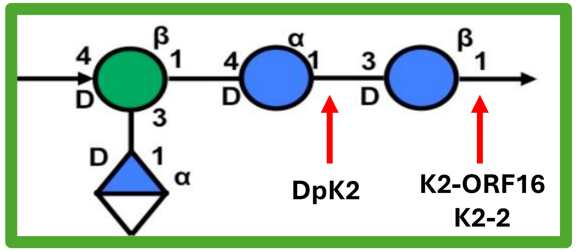

K2
CPS repeating unit

→4)-β-D-Man*p*-(1→4)-α-D-Glc*p*-(1→3)-β-D-Glc*p*-(1→
3
↑
1
α-D-Glc*p*A

DpK2 (cleaves the 1→3 linkage between α-D-Glc*p* and β-D-Glc*p*)

K2-ORF16, K2-2 (cleave the 1→ linkage after β-D-Glc*p*)

Supplementary Figure 2. continued

**K-type target**	**K2**/K13	**K2***	**K2***	**K2***	**K2**/K13
**Group**	Group 1				
**Protein name**	B1dep	DpK2	Depo32	BMacgp22	**gp81**
**Length**	907 aa	907 aa	907 aa	907 aa	897 aa
**No. of proteins in the group**	5 proteins				
**Predicted protein structure (AlphaFold3)**	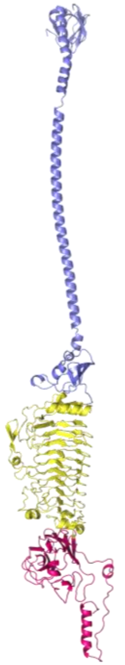	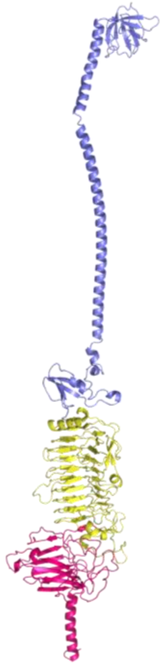	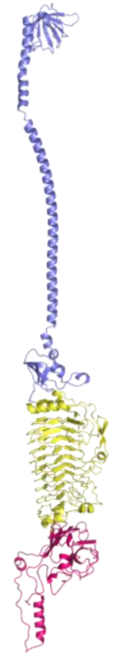	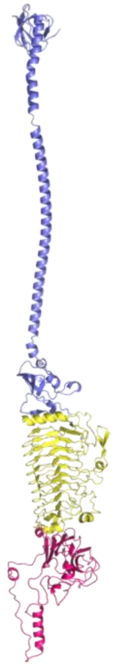	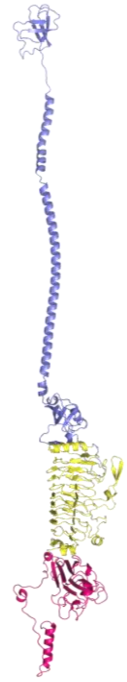

*protein was not tested against K13

Cleavage site DpK2[4]

K2
CPS repeating unit

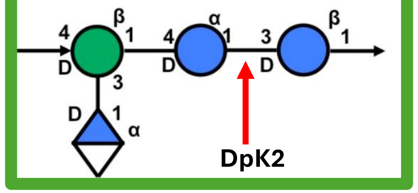

→4)-β-D-Man*p*-(1→4)-α-D-Glc*p*-(1→3)-β-D-Glc*p*-(1→
3
↑
1
α-D-Glc*pA*

DpK2

Active Site Residues **Depo32**[7]
Glu423, Glu545, Asp497, Asp546

Supplementary Figure 2. continued

Cleavage site[5,6]

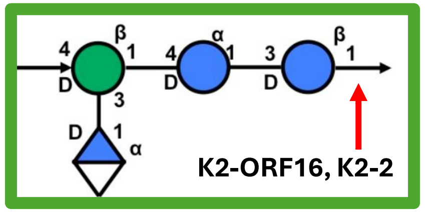

Active Site Residues **K2-2**
Glu267, Glu323[6]

**K-type target**	**K2***	**K2***	**K2**/K13	**K2**/K13	**K2***	**K2***	**K2/**K13	**K2**/K13
**Group**	Group 2							
**Protein name**	NPatgp22	K2-ORF16	**KP24gp196**	Kpv74_56	Dep1979	GBH038_054	K2-2	**KLEO13gp09**
**Length**	763 aa	668 aa	660 aa	577 aa	577 aa	577 aa	577 aa	524 aa
**No. of proteins in the group**	8 proteins							
**Predicted protein structure (AlphaFold3)**	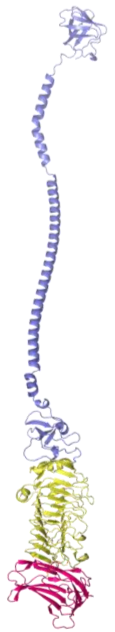	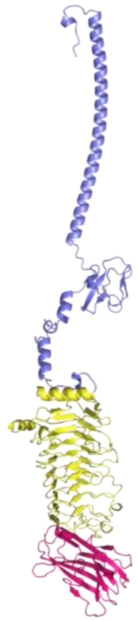	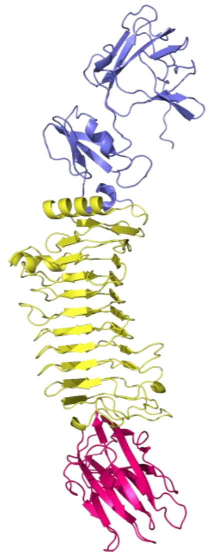	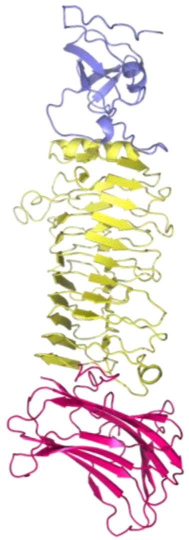	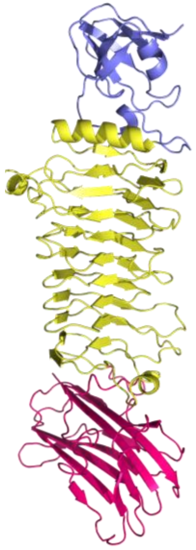	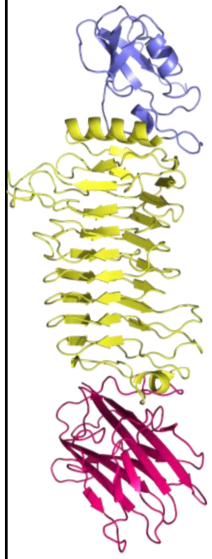	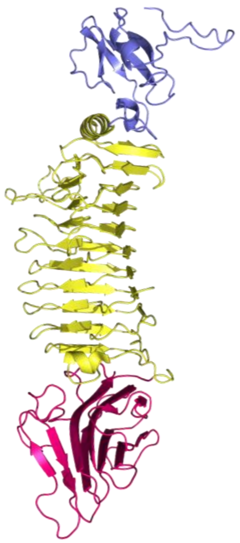	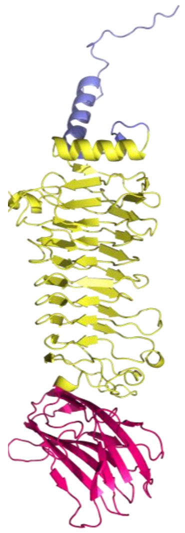

*protein was not tested against K13

Supplementary Figure 2. continued

K-type target	**K3.2/**KL110/ KL116/KL117	**K3.2/**KL116	**K3.1**	**K3.1**/K26**/** KL173/	**K5**		**K6**
**Group**	Group 1		Group 2		Group 1		Group 1
**Protein name**	**KP32gp37**	**CDS_0197**	**0391_03**	**CDS_0182**	**dep1011**	K5-4 ORF38	**CDS_0189**
**Length**	869 aa	774 aa	736 aa	598 aa	857 aa	684 aa	874 aa
**No. of proteins in the group**	2 proteins		2 proteins		2 proteins		1 protein
**Predicted protein structure (AlphaFold3)**	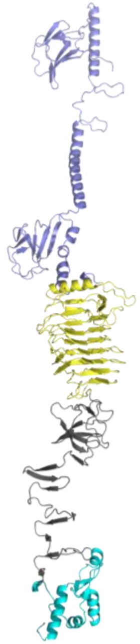	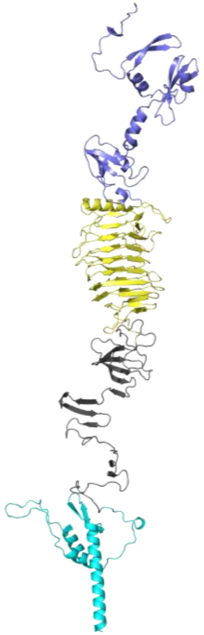	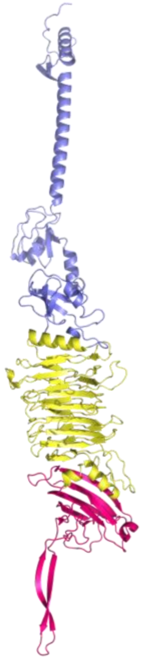	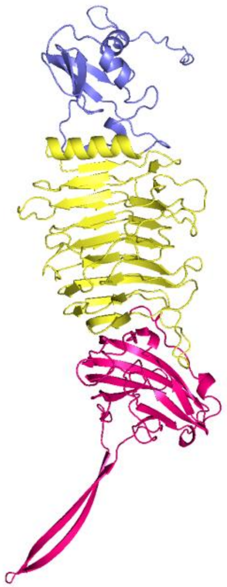	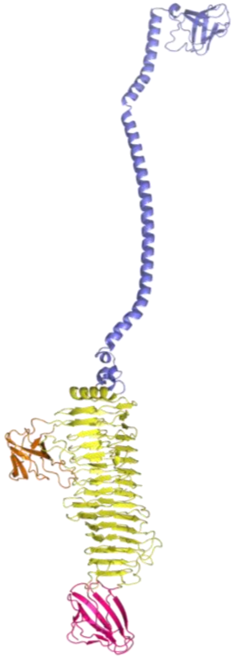	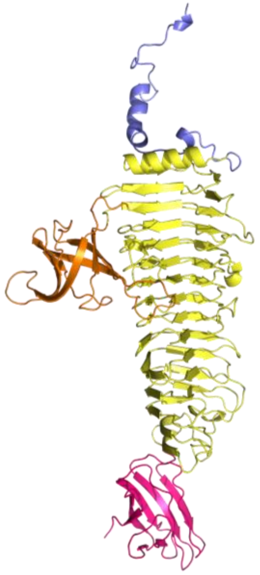	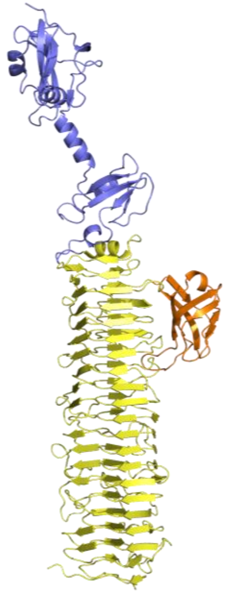

Supplementary Figure 2. continued

K-type target	**K7**	**K8**	**K9**/KL184	**K10**	**K11**/KL181	**K11***
**Group**	Group 1	Group 1	Group 1	Group 1	Group 1	
**Protein name**	**K7dep**	**K5-4 ORF37**	**FKANgp229**	**KLEO25gp59**	**K11gp17**	S1-1
**Length**	1180 aa	749 aa	578 aa	513 aa	875 aa	702 aa
**No. of proteins in the group**	1 protein	1 protein	1 protein	1 protein	2 proteins	
**Predicted protein structure (AlphaFold3)**	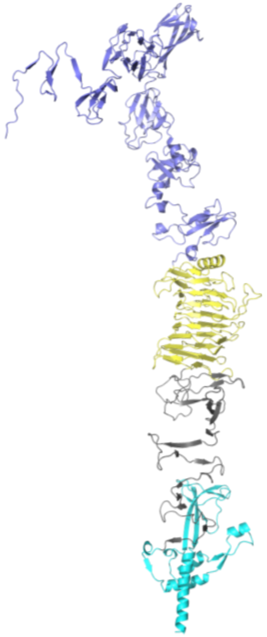	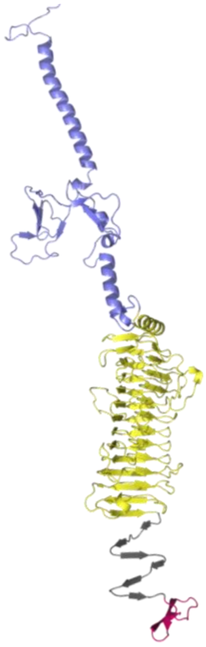	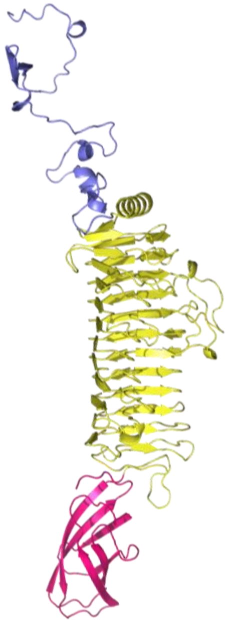	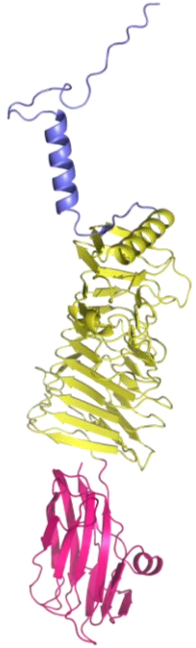	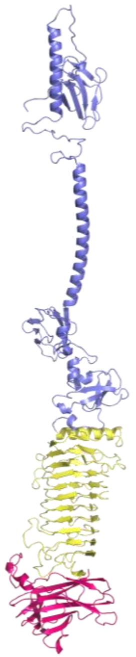	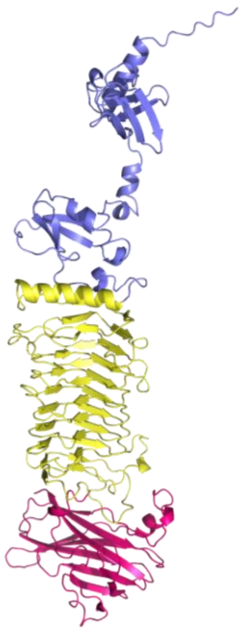

*protein was not tested against KL181

Supplementary Figure 2. continued

**K-type target**	**K13**/K2				
**Group**	Group 1		Group 2		
**Protein name**	B1dep	**gp81**	**KP24gp196**	K2-2	**KLEO13gp09**
**Length**	907 aa	897 aa	660 aa	577 aa	524 aa
**No. of proteins in the group**	2 proteins		3 proteins		
**Predicted protein structure (AlphaFold3)**	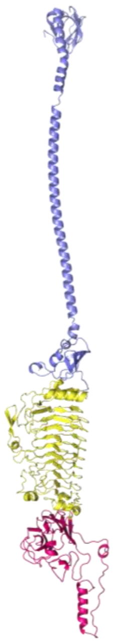	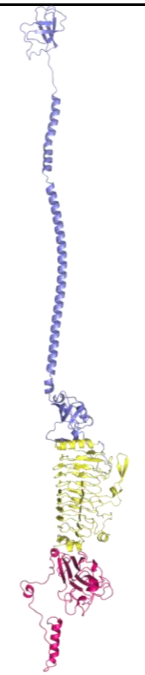	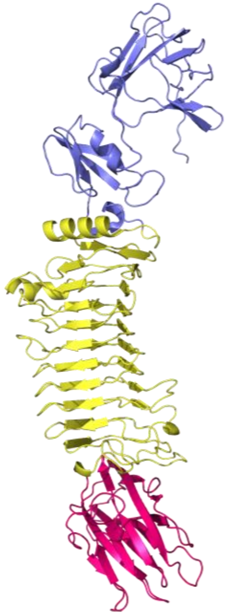	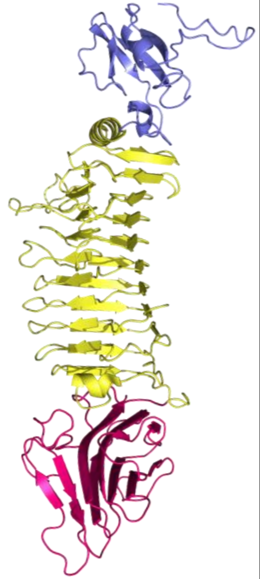	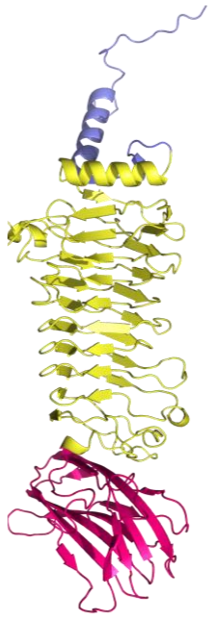

Supplementary Figure 2. continued

**K-type target**	**K14**		**K19**	**K20**	**K20**/KL132
**Group**	Group 1		Group 1	Group 1	Group 2
**Protein name**	**0574_17**	**KLEO26gp185**	**KP24gp304**	**Kl-dep**	**K20dep**
**Length**	873 aa	834 aa	755 aa	790 aa	723 aa
**No. of proteins in the group**	2 proteins		1 protein	1 protein	1 protein
**Predicted protein structure (AlphaFold3)**	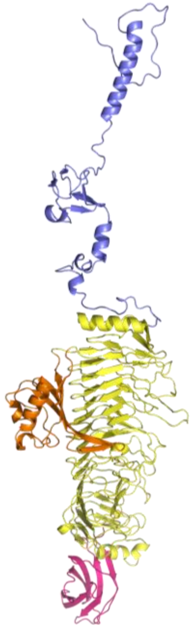	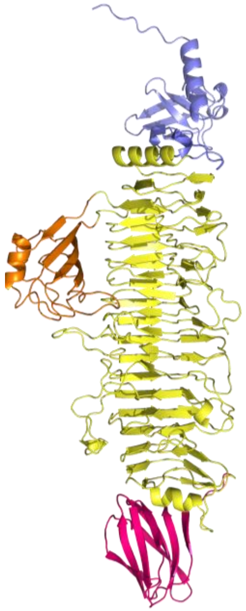	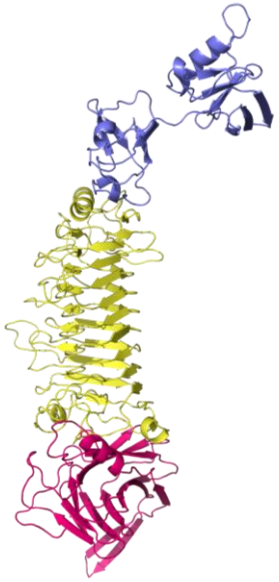	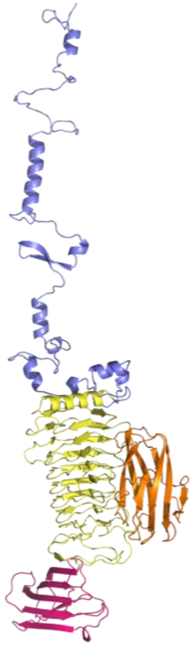	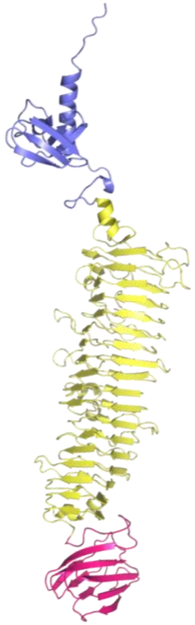

Supplementary Figure 2. continued

K-type target	K21*	K21/KL163	KL21*
**Group**	Group 1		
**Protein name**	S1-3	**KP32gp38**	RBP2
**Lenght**	651 aa	576 aa	575 aa
**No. of proteins in the group**	3 proteins		
**Predicted protein structure (AlphaFold3)**	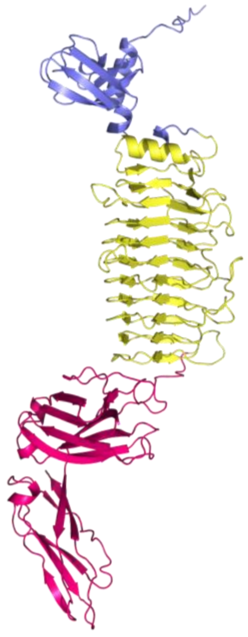	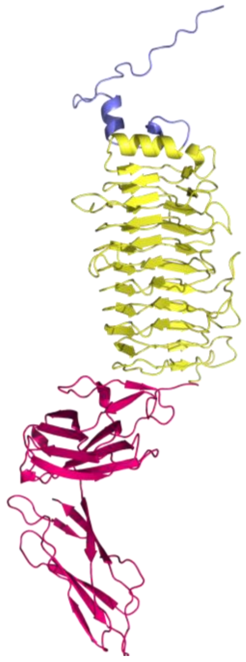	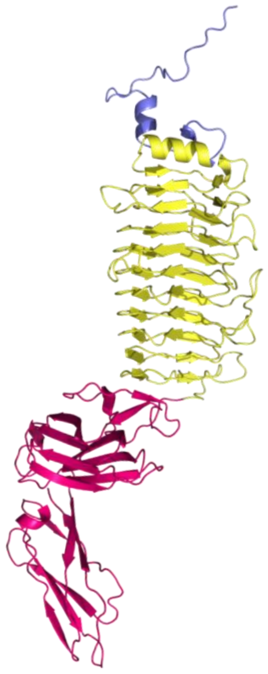

*protein was not tested against KL163

Cleavage site RBP2 [8]

K21 CPS repeating unit

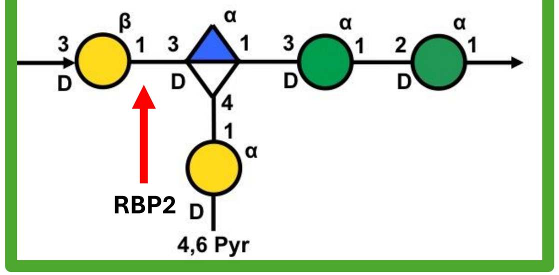

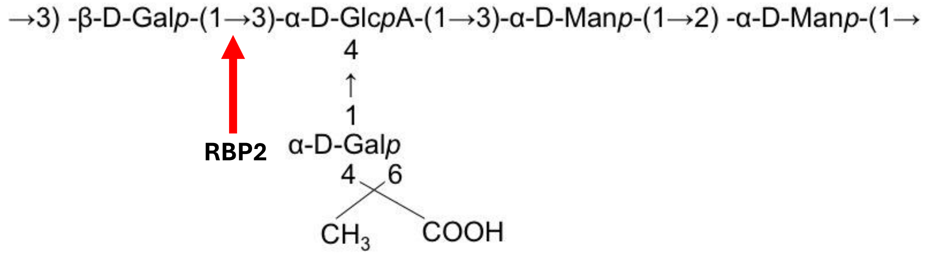

Active Site Residues **KP32gp38** [9]

Glu239, Asp229, Asp241, Glu170

Supplementary Figure 2. continued

K-type target	**K22**/K37/KL111	**K23**				
**Group**	Group 1	Group 1				
**Protein name**	**KLEO13gp10**	**1409_59**	**1441_47**	**1248_57**	DepS8	Dep622
**Length**	777 aa	852 aa	704 aa	704 aa	607 aa	555 aa
**No. of proteins in the group**	1 protein	5 proteins				
**Predicted protein structure (AlphaFold3)**	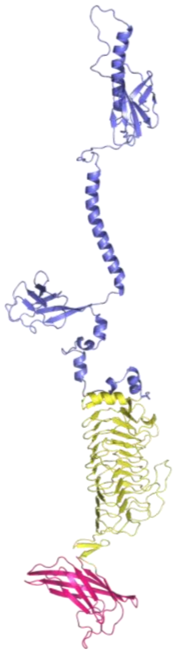	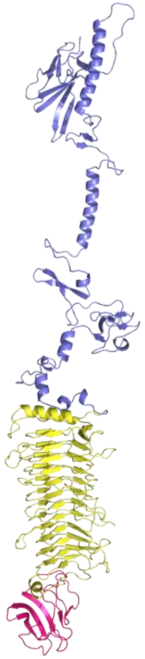	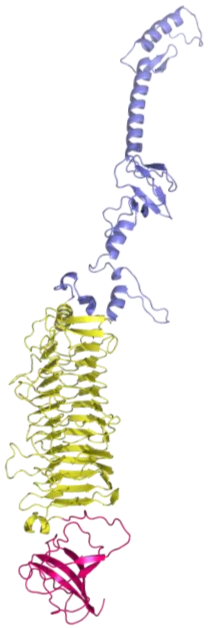	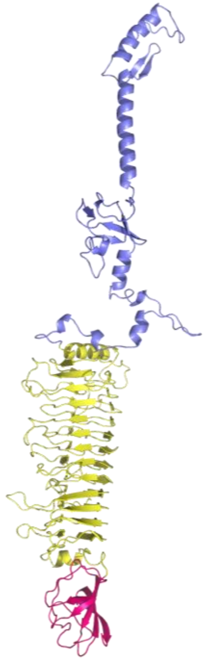	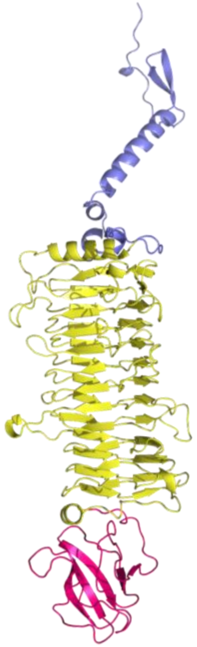	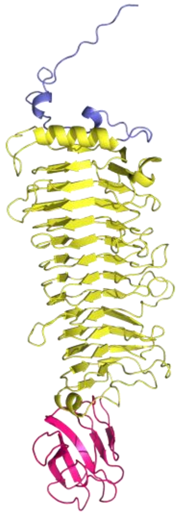

Supplementary Figure 2. continued

**K-type target**	**K25**		**K26**/KL173/K3.1	**K27**	
**Group**	Group 1		Group 1	Group 1	
**Protein name**	**KP24gp300**	S2-2	**CDS_0182**	K27dep	**gp12**
**Length**	598 aa	584 aa	598 aa	1294 aa	1242 aa
**No. of proteins in the group**	2 proteins		1 protein	2 proteins	
**Predicted protein structure (AlphaFold3)**	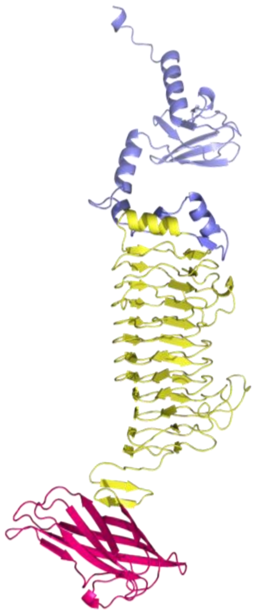	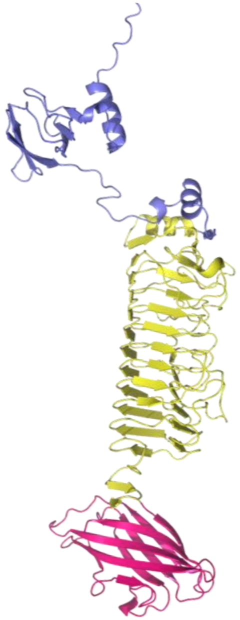	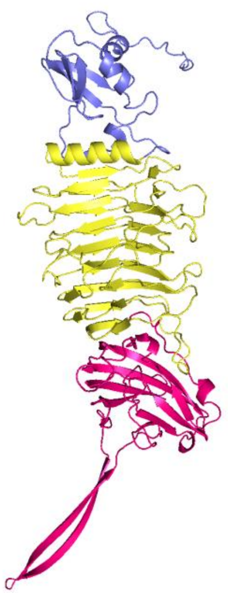	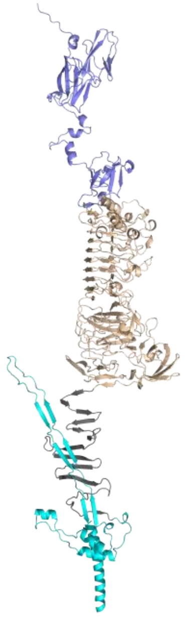	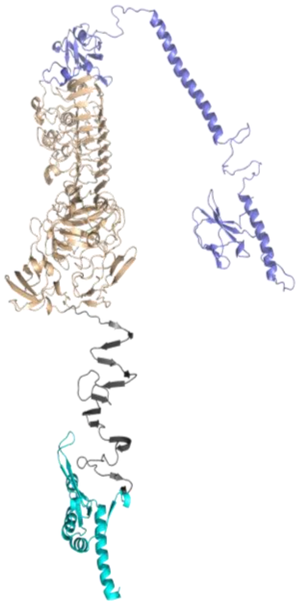

Supplementary Figure 2. continued

**K-type target**	**K28**		**K30***	**K30**/K69		**K31**	
**Group**	Group 1		Group 1			Group 1	
**Protein name**	**1251_37**	**FKANgp225**	Dop5	**K5-2 ORF37**	S2-6	**KLEO26gp187**	**K11gp0043**
**Length**	677 aa	605 aa	792 aa	792 aa	767 aa	675 aa	596 aa
**No. of proteins in the group**	2 proteins		3 proteins			2 proteins	
**Predicted protein structure (AlphaFold3)**	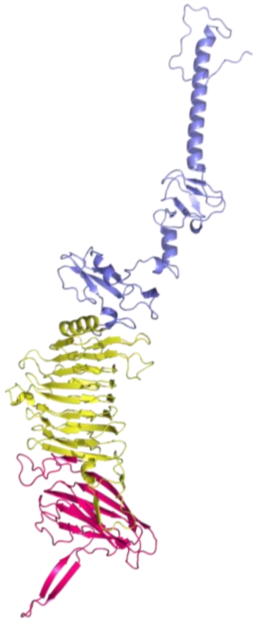	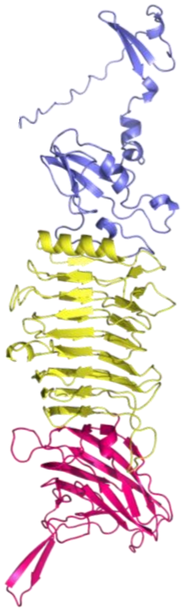	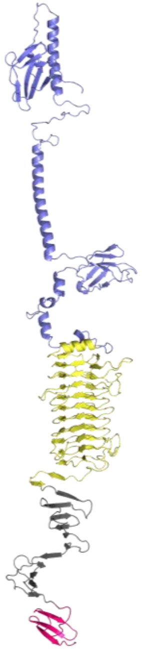	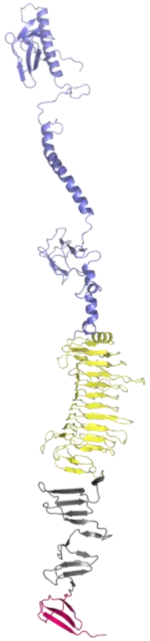	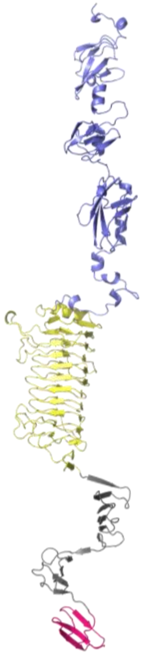	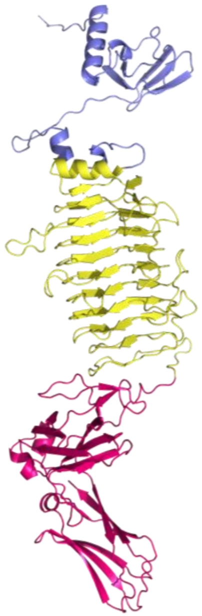	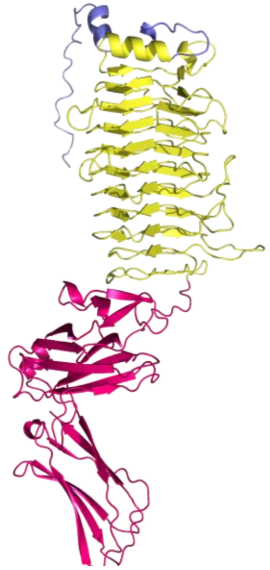

*protein was not tested against K69

Supplementary Figure 2. continued

K-type target	**K32**	**K35**				**K37**/KL111/K22	**K38**
**Group**	Group 1	Group 1			Group 2	Group 1	Group 1
**Protein name**	**914_77**	**KP24gp301**	**CDS_0180**	S2-3	**KLEO26gp181**	**KLEO13gp10**	**617_77**
**Length**	536 aa	915 aa	915 aa	779 aa	1039 aa	777 aa	1258 aa
**No. of proteins in the group**	1 protein	3 proteins			1 protein	1 protein	1 protein
**Predicted protein structure (AlphaFold3)**	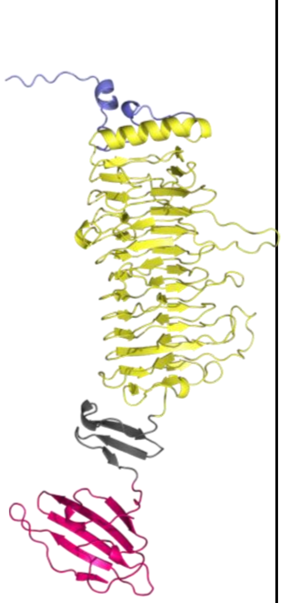	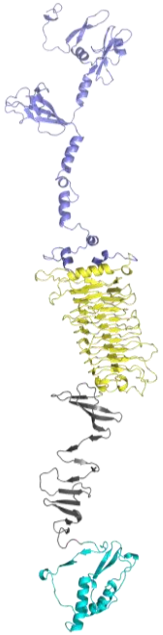	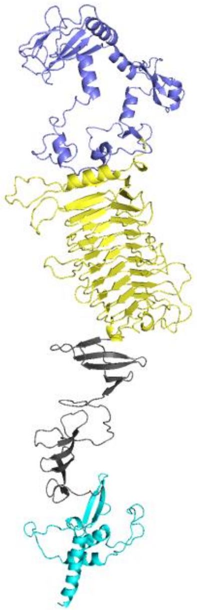	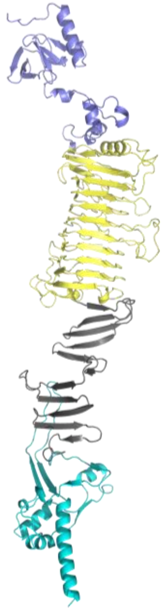	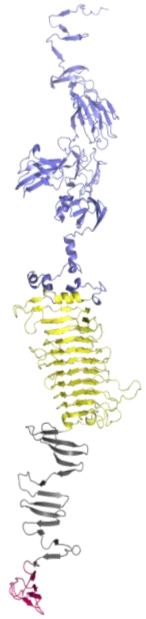	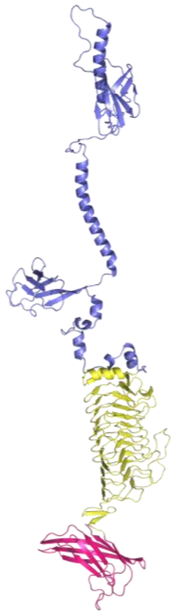	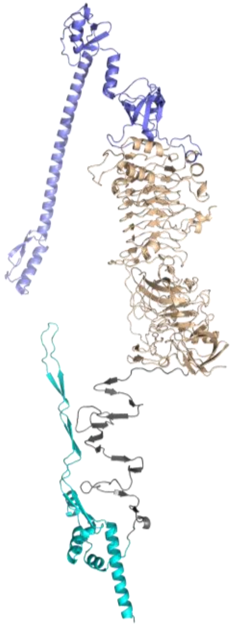

Supplementary Figure 2. continued

**K-type target**	**K39**	**K46**/KL146	**K46**	**K47.1**			**K47.2**	
**Group**	Group 1	Group 1	Group 2	Group 1			Group 2	
**Protein name**	**FKANgp227**	**KP24gp308**	**248_38**	Dep42	Dpo42	**KLEO27gp5**	Dpo43	**KLEO27gp6**
**Length**	820 aa	737 aa	693 aa	793 aa	793 aa	793 aa	641 aa	641 aa
**No. of proteins in the group**	1 protein	1 protein	1 protein	3 proteins			2 proteins	
**Predicted protein structure (AlphaFold3)**	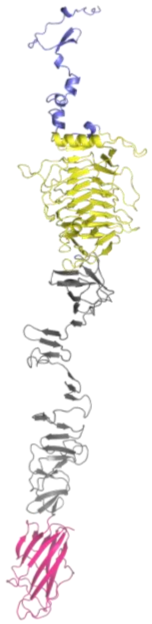	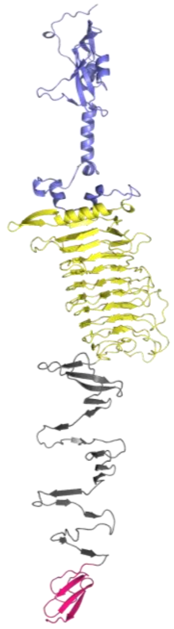	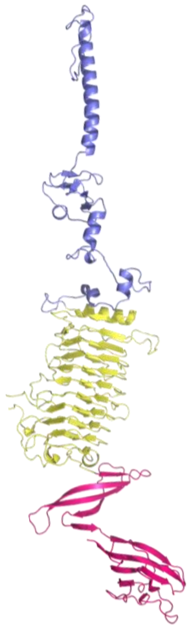	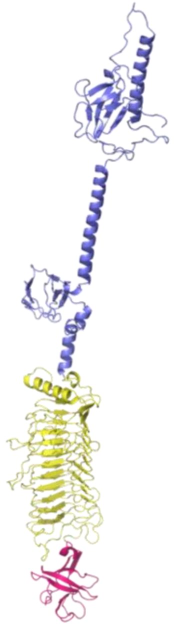	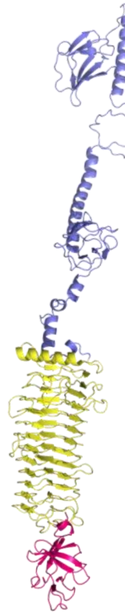	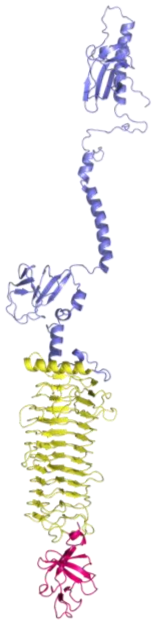	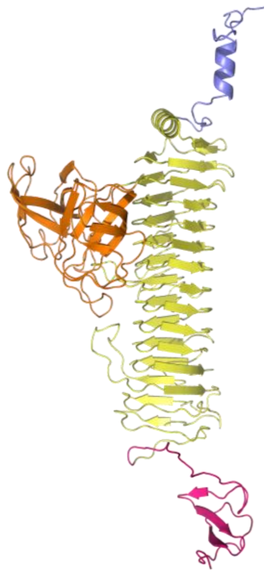	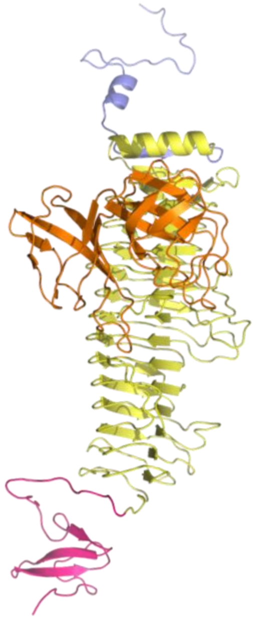

Supplementary Figure 2. continued

Cleavage site gp531[10]

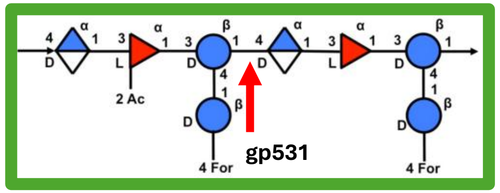

**K-type target**	**K51**	**K52**	**K54**				
**Group**	Group 1	Group 1	Group 1				
**Protein name**	**GBH019_279**	**434_33**	**gp531**	Dep_Z	Dep_C	Dep_Y	**CDS_0179**
**Length**	809 aa	859 aa	895 aa	708 aa	694 aa	692 aa	601 aa
**No. of proteins in the group**	1 protein	1 protein	5 proteins				
**Predicted protein structure (AlphaFold3)**	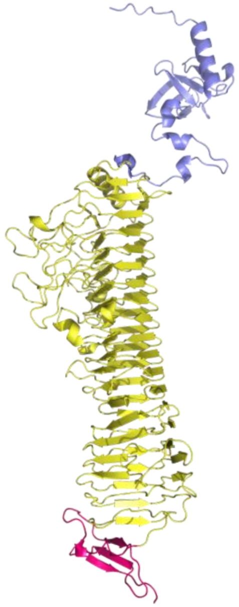	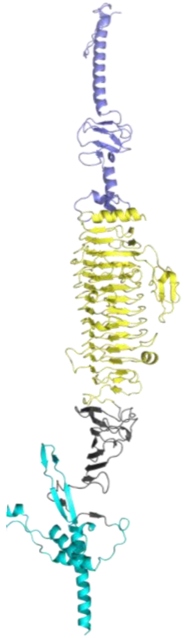	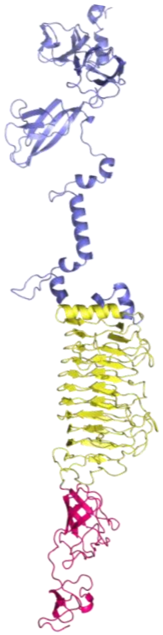	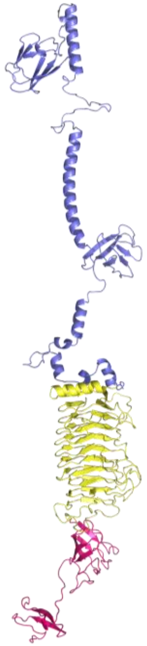	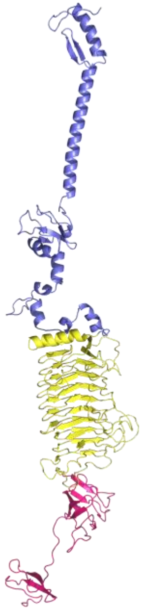	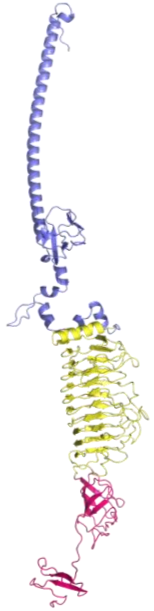	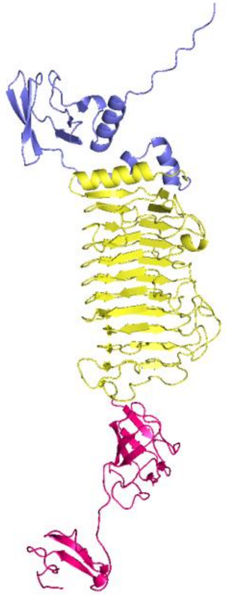

Supplementary Figure 2. continued

Cleavage site Dep_kpv767 Dep_kpv79[11]

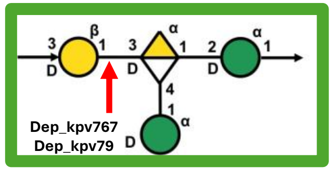

*protein was not tested against K68

K-type target	K56	K57*	K57/K68	K57*	K57/K68	K57*	K57/K68
**Group**	Group 1	Group 1					
**Protein name**	**K56dep**	Dep_kpv767	**CDS_0060**	Dep_kpv79	**gp157**	Dep_ZX1	**KLEO7gp25**
**Length**	678 aa	843 aa	739 aa	721 aa	628 aa	614 aa	570 aa
**No. of proteins in the group**	1 protein	6 proteins					
**Predicted protein structure (AlphaFold3)**	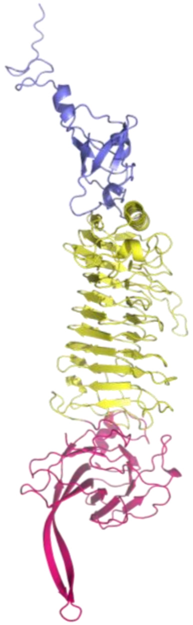	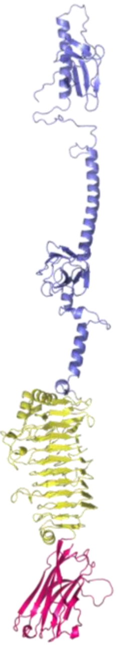	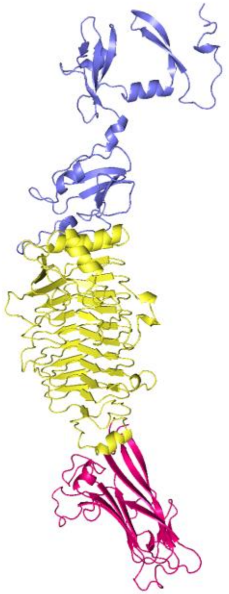	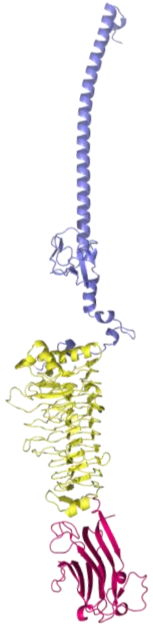	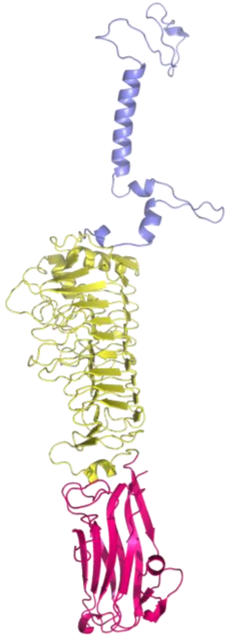	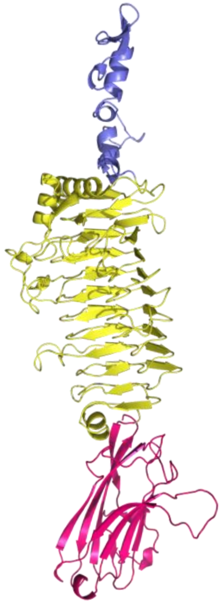	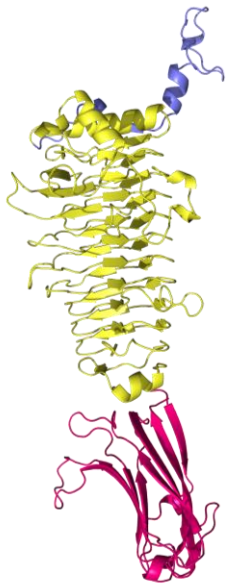

Supplementary Figure 2. continued

K-type target	K60		K61	K62			
**Group**	Group 1		Group 1	Group 1		Group 2	
**Protein name**	**1723_59**	**1724_71**	**KP24gp310**	**0367_12**	**0391_11**	K62-Dpo30	**914_74**
**Length**	951 aa	843 aa	679 aa	851 aa	839 aa	692 aa	666 aa
**No. of proteins in the group**	2 proteins		1 protein	2 proteins		2 proteins	
**Predicted protein structure (AlphaFold3)**	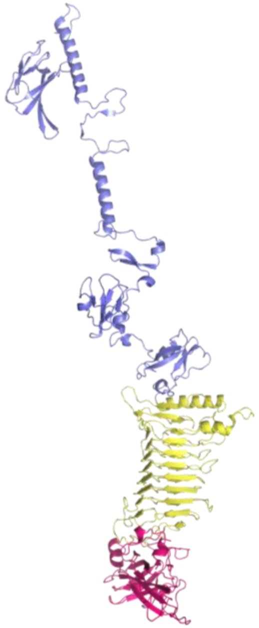	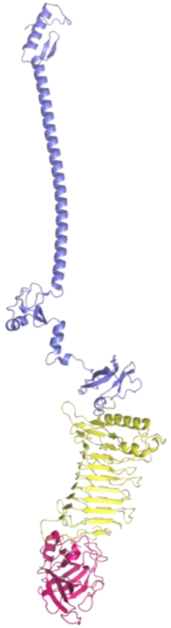	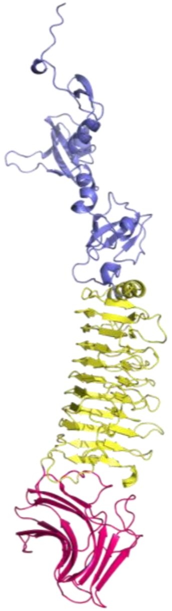	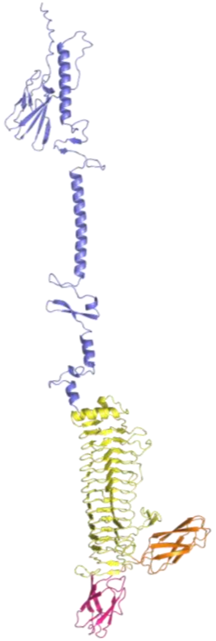	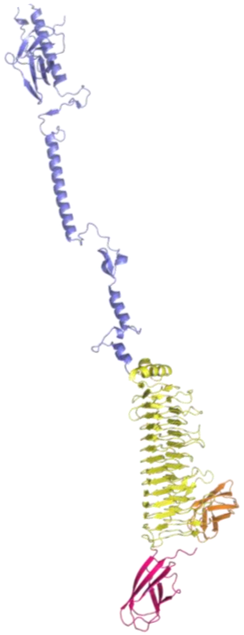	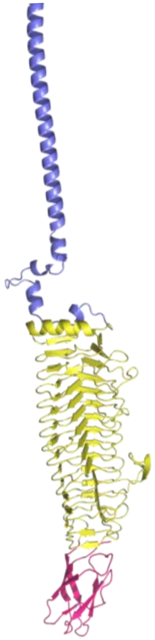	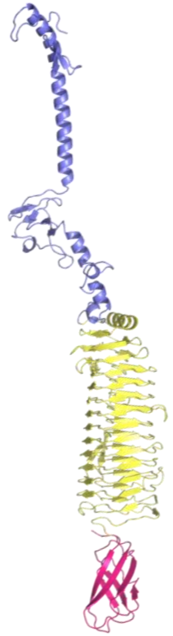

Supplementary Figure 2.
continued

K-type target	K63	
Group	Group 1	
Protein name	KP36gp50	KP34gp57
Length	883 aa	630 aa
No. of proteins in the group	2 proteins	
Predicted protein structure (AlphaFold3)	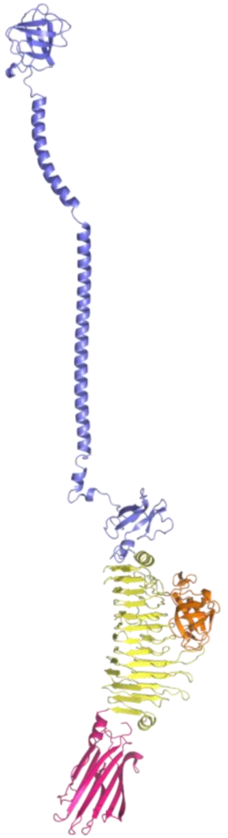	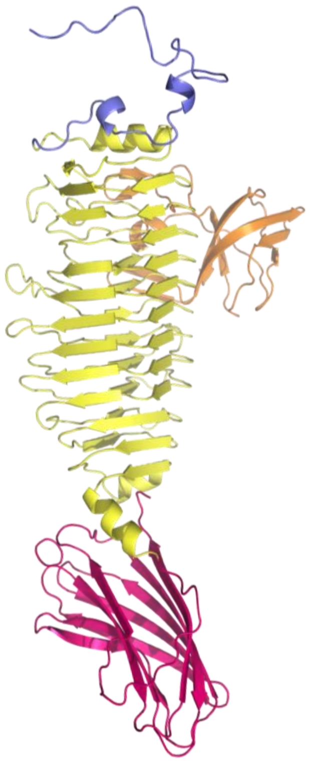

Cleavage site KP34gp57[12]

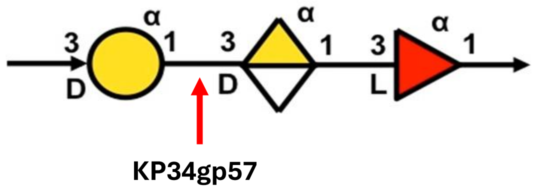

→3)-α-D-Gal*p*-(1→3)-α-D-Gal*p*A-(1→3)-α-L-Fuc*p*-(1→

KP34gp57

K63
CPS repeat unit

Active Site Residues **KP34gp57**
Glu266/Glu300

Supplementary Figure 2. continued

**K-type target**	**K64***		**K64/**KL178	**K64***	**K64/**KL178		
**Group**	Group 1				Group 2		
**Protein name**	P510dep	K64-ORF41	**KLEO28gp32**	S2-5	**1091_44**	**CDS_0184**	**KP24gp303**
**Length**	1017 aa	1017 aa	1017 aa	996 aa	880 aa	739 aa	661 aa
**No. of proteins in the group**	4 proteins				3 proteins		
**Predicted protein structure (AlphaFold3)**	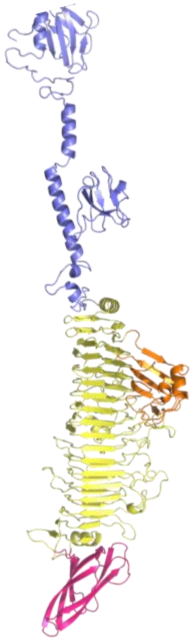	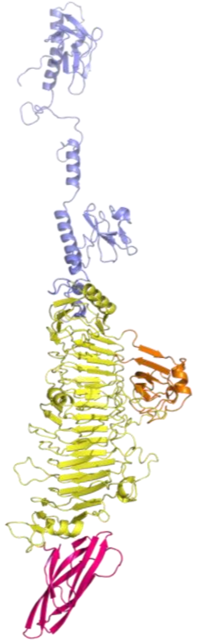	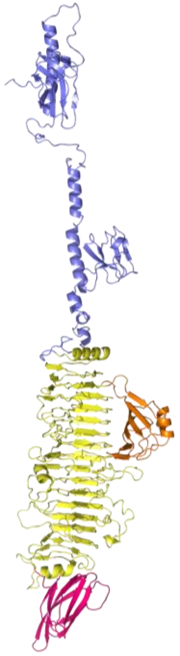	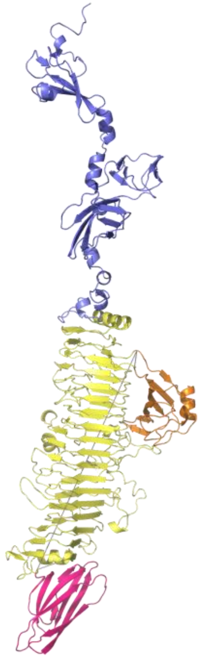	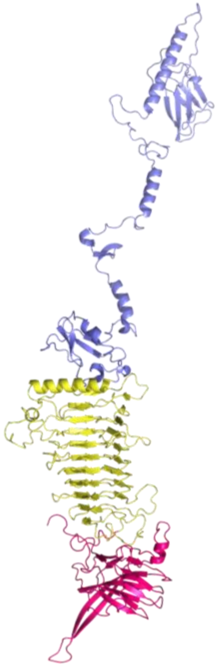	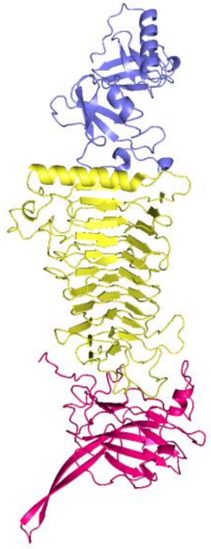	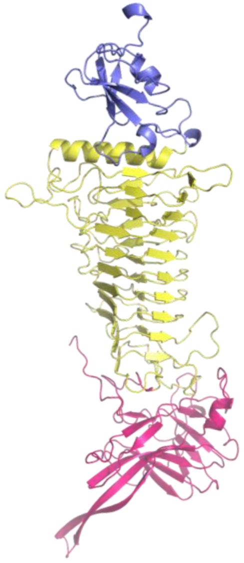

*protein was not tested against KL178

Supplementary Figure 2. continued

**K-type target**	**K68**/K57			**K69**/K30		**K74/K80**
**Group**	Group 1			Group 1		Group 1
**Protein name**	**CDS_0060**	**gp157**	**KLEO7gp25**	**K5-2 ORF37**	S2-6	**CDS_0190_1**
**Length**	739 aa	628 aa	570 aa	792 aa	767 aa	690 aa
**No. of proteins in the group**	3 proteins			2 proteins		1 protein
**Predicted protein structure (AlphaFold3)**	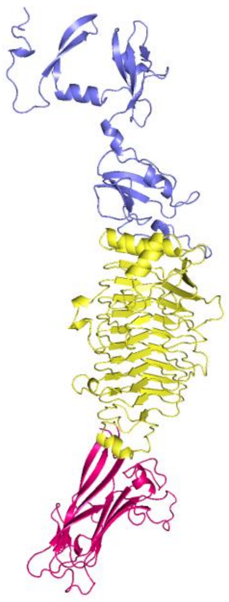	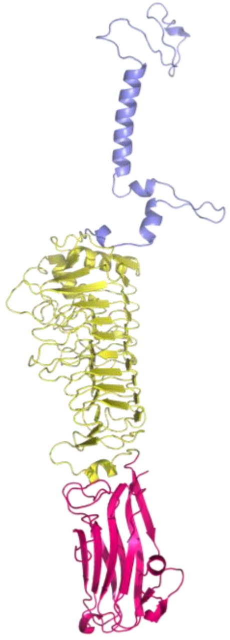	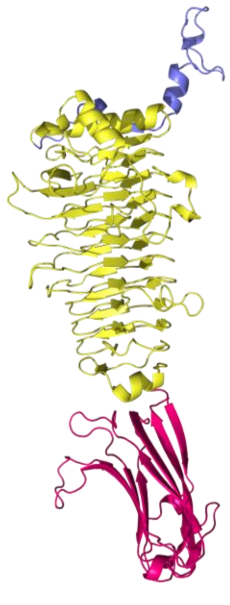	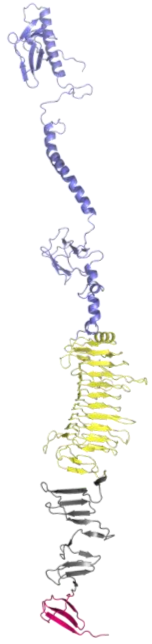	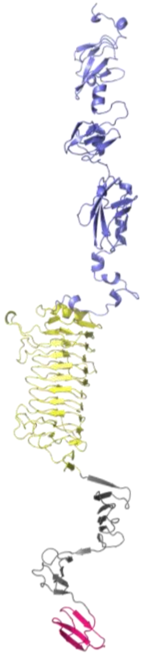	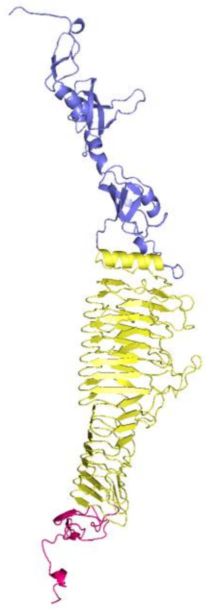

Supplementary Figure 2. continued

K-type target	KL101 (KN1)/KL120/KL170		KL102 (KN2)	KL108	
Group	Group 1		Group 1	Group 1	
Protein name	**KN1dep**	**Dp42**	**ORF96**	Dep108.2	**Dep108.1**
Length	820 aa	820 aa	1245 aa	592 aa	590 aa
No. of proteins in the group	2 proteins		1 protein	2 proteins	
Predicted protein structure (AlphaFold3)	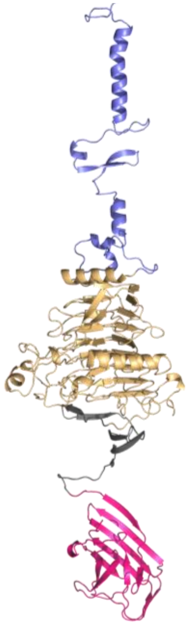	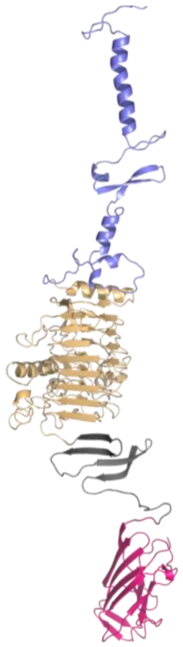	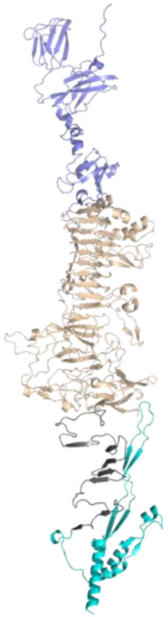	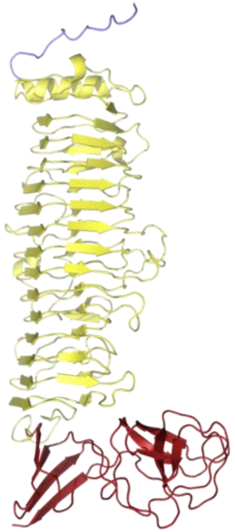	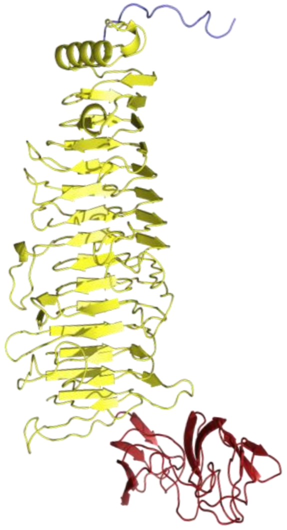

Cleavage site Dep108.1, Dep108.2[13]

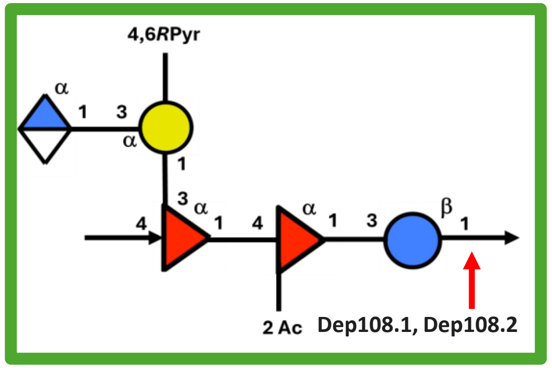

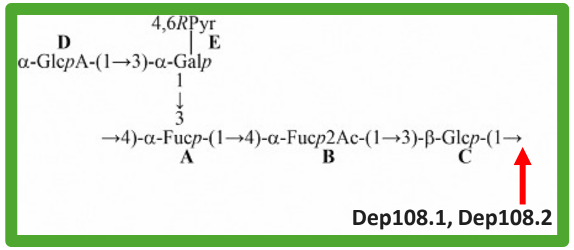

KL108
CPS repeat unit

Supplementary Figure 2. continued

K-type target	**KL110**/KL116/KL117/K3.2	**KL110**/KL116	**KL111**/K22/K37	**KL111**		**KL114**
**Group**	Group 1	Group 2	Group 1			Group 1
**Protein name**	**KP32gp37**	**FKANgp223**	**KLEO13gp10**	**184_43**	**FKANgp232**	**KP24gp307**
**Length**	869 aa	581 aa	777 aa	639 aa	623 aa	742 aa
**No. of proteins in the group**	1 protein	1 protein	3 proteins			1 protein
**Predicted protein structure (AlphaFold3)**	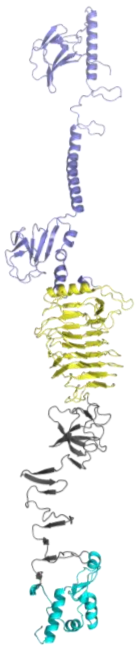	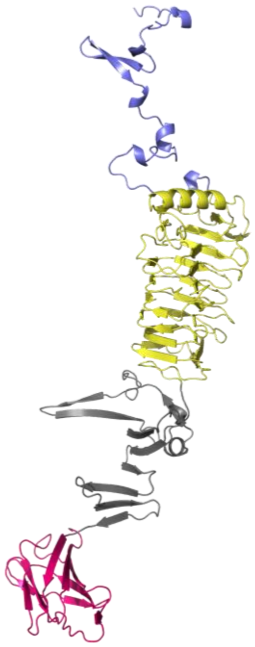	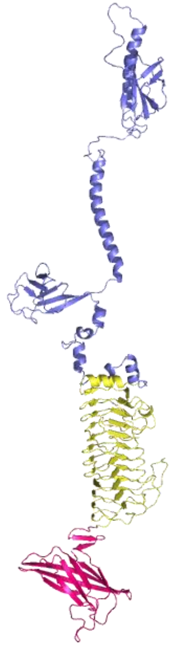	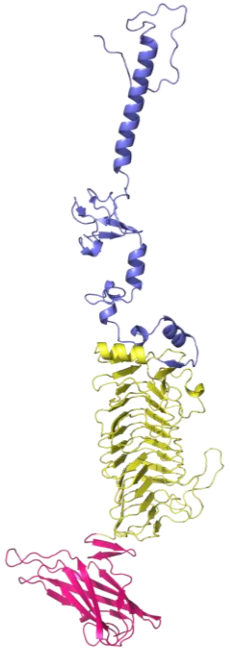	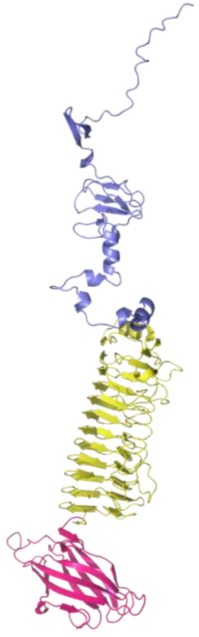	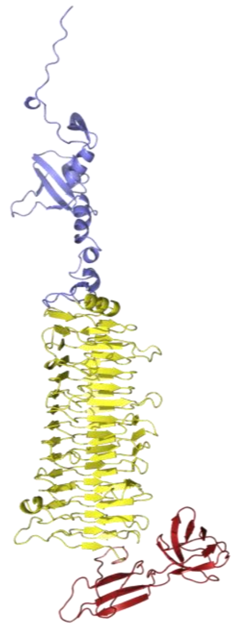

Supplementary Figure 2. continued

**K-type target**	**KL116/KL117**/K3.2/KL110	**KL116**/KL3.2	**KL116/**KL110	**KL120**/KL101 (KN1)/KL170		**KL121 (KN5)**	**KL121**
**Group**	Group 1		Group 2	Group 1		Group 1	
**Protein name**	**KP32gp37**	**CDS_0187**	**FKANgp223**	**KN1dep**	Dp42	S2-1	**CDS_0186**
**Length**	869 aa	774 aa	581 aa	820 aa	820 aa	1193 aa	700 aa
**No. of proteins in the group**	2 proteins		1 protein	2 proteins		2 proteins	
**Predicted protein structure (AlphaFold3)**	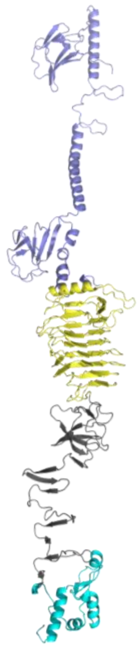	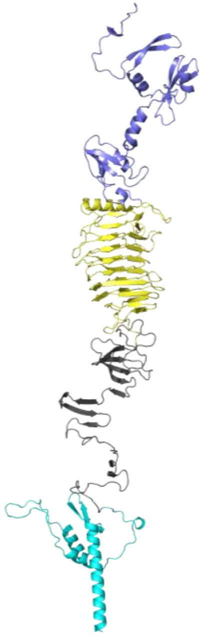	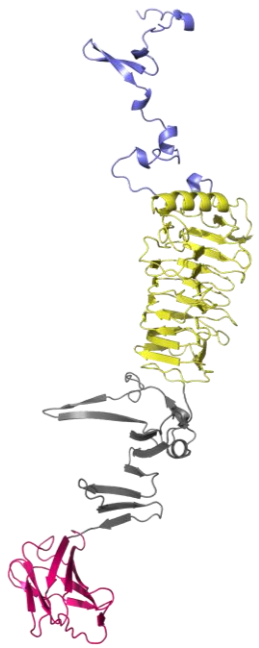	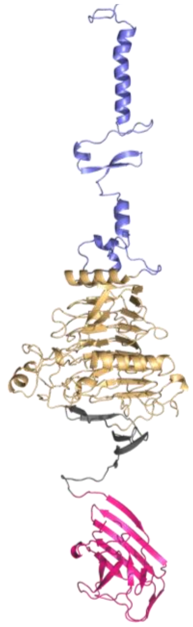	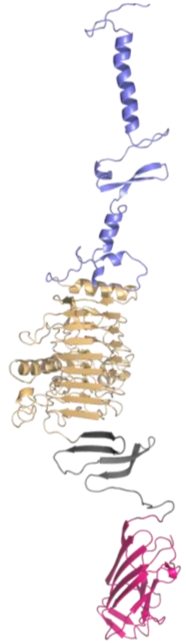	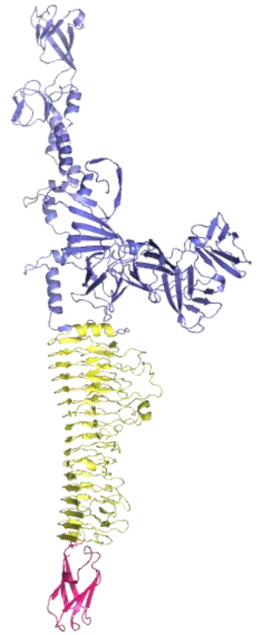	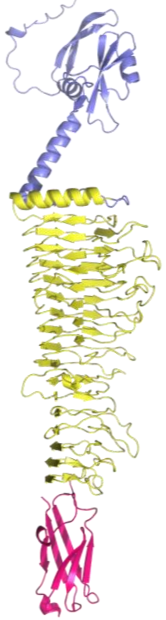

Supplementary Figure 2. continued

K-type target	KL122	KL124 (KN4)	KL124	KL124 (KN4)	KL127	KL134	KL137
Group	Group 1	Group 1	Group 2		Group 1	Group 1	Group 1
Protein name	0496_72	KN4dep	CDS_0178	S1-2	319_37	KP24gp168	KP24gp306
Length	968 aa	850 aa	803 aa	736 aa	794 aa	881 aa	883 aa
No. of proteins in the group	1 protein	1 protein	2 proteins		1 protein	1 protein	1 protein
Predicted protein structure (AlphaFold3)	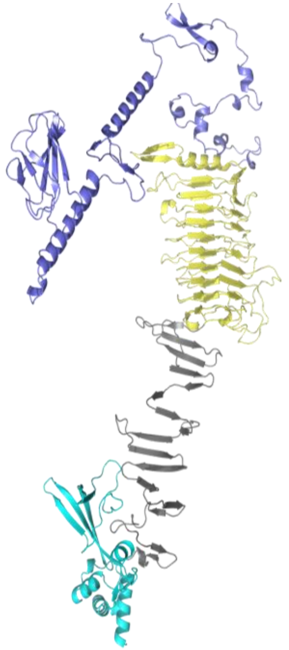	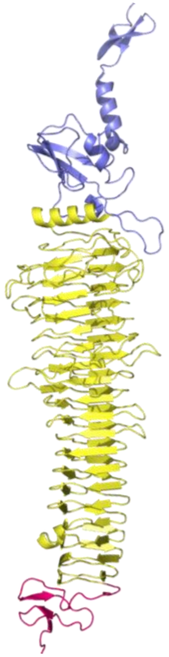	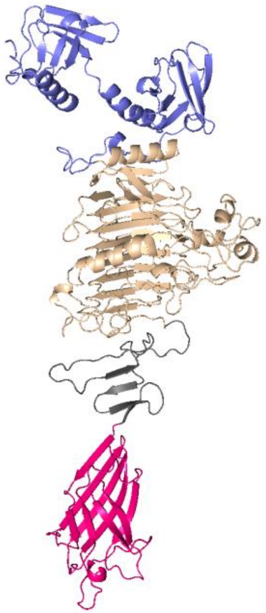	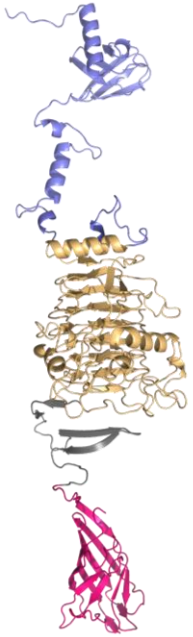	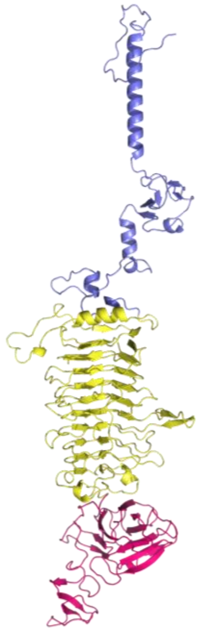	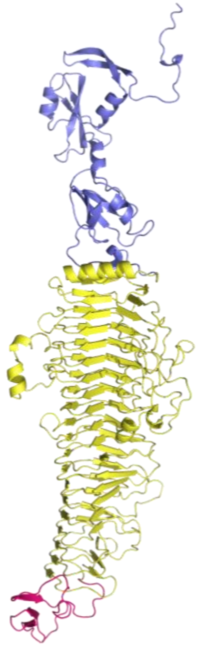	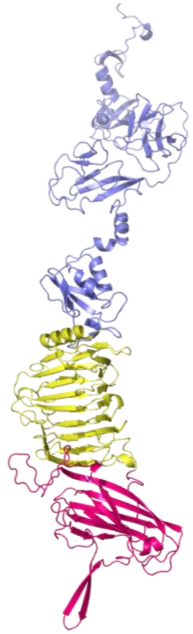

Supplementary Figure 2. continued

K-type target	**KL143**	**KL146**/K46	**KL148**		**KL151**
**Group**	Group 1	Group 1	Group 1	Group 2	Group 1
**Protein name**	**738_68**	**KP24gp308**	**CDS_0191**	**FKANgp220**	**CDS_0185**
**Length**	742 aa	737 aa	728 aa	591 aa	952 aa
**No. of proteins in the group**	1 protein	1 protein	1 protein	1 protein	1 protein
**Predicted protein structure (AlphaFold3)**	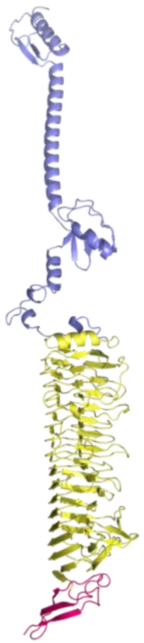	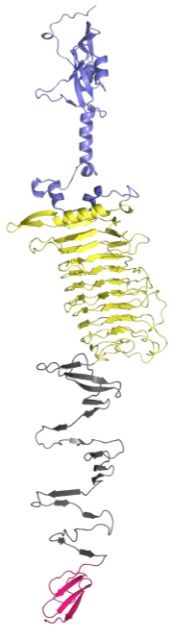	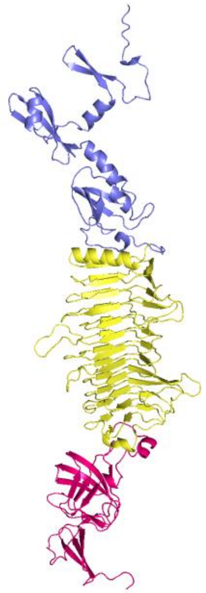	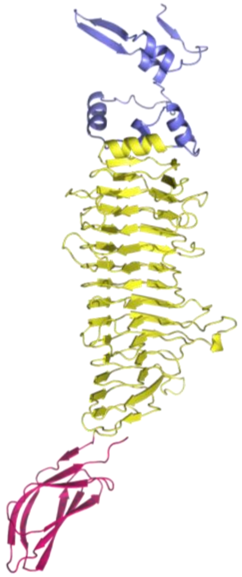	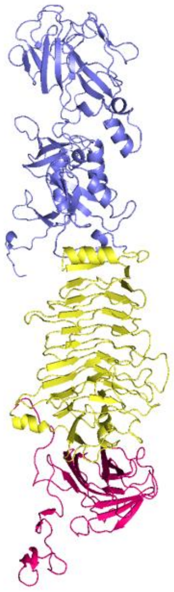

Supplementary Figure 2. continued

**K-type target**	**KL153**	**KL158**	**KL163**/K21	**KL169**
**Group**	Group 1	Group 1	Group 1	Group 1
**Protein name**	**FKANgp217**	**KP24gp309**	**KP32gp38**	**P560dep**
**Length**	644 aa	751 aa	576 aa	802 aa
**No. of proteins in the group**	1 protein	1 protein	1 protein	1 protein
**Predicted protein structure (AlphaFold3)**	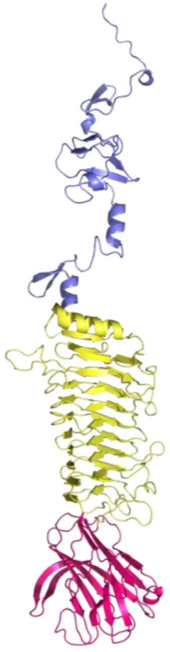	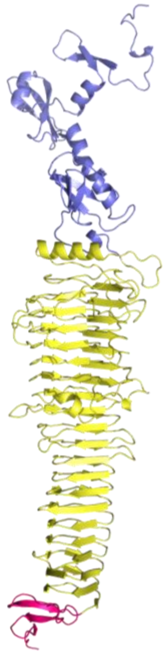	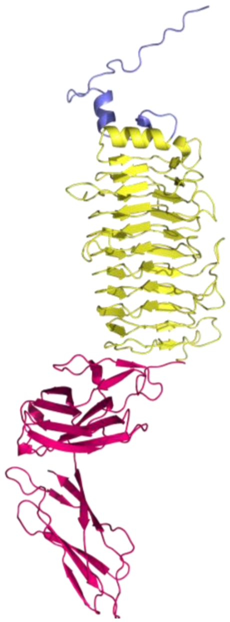	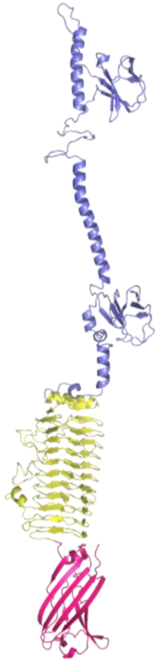

Supplementary Figure 2. continued

**K-type target**	**KL170**/KL101 (KN1)/KL120		**KL173**/K3.1/K26
**Group**	Group 1		Group 1
**Protein name**	**KN1dep**	**Dp42**	**CDS_0182**
**Length**	820 aa	820 aa	598 aa
**No. of proteins in the group**	2 proteins		1 protein
**Predicted protein structure (AlphaFold3)**	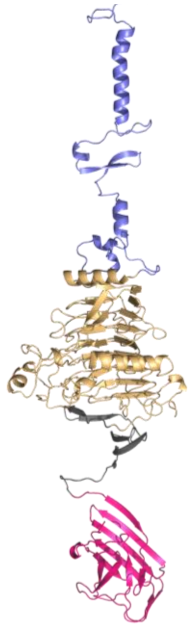	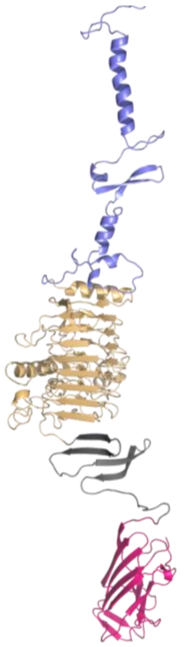	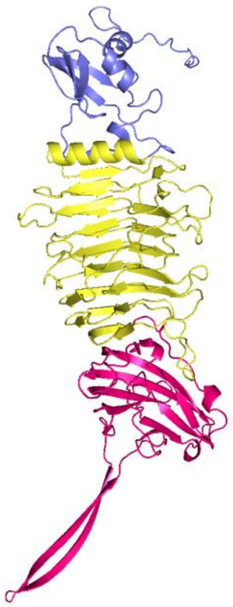

Supplementary Figure 2. continued

**K-type target**	**KL178**/K64				**KL181**/K11	**KL183 (KN3)**	**KL184**/K9
**Group**	Group 1	Group 2			Group 1	Group 1	Group 1
**Protein name**	**KLEO28gp32**	**1091_44**	**CDS_0184**	**KP24gp303**	**K11gp17**	**KN3dep**	**FKANgp229**
**Length**	1017 aa	880 aa	739 aa	661 aa	875 aa	792 aa	578 aa
**No. of proteins in the group**	1 protein	3 proteins			1 protein	1 protein	1 protein
**Predicted protein structure (AlphaFold3)**	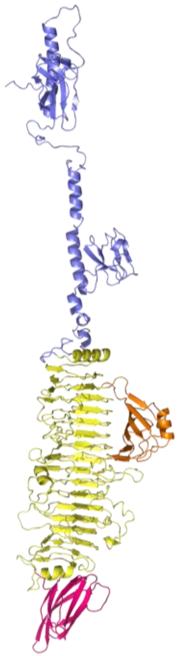	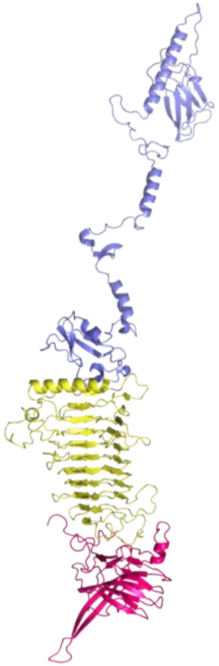	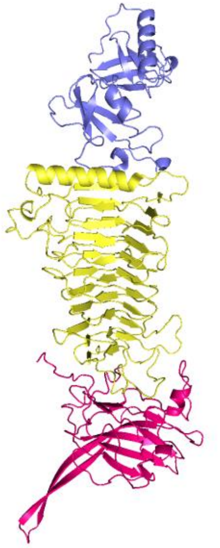	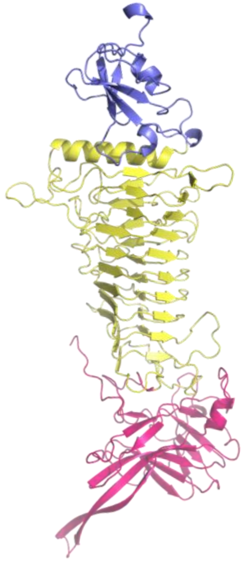	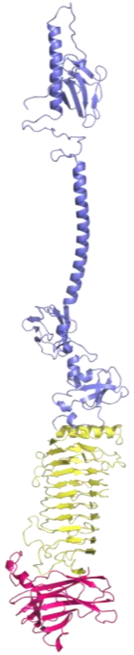	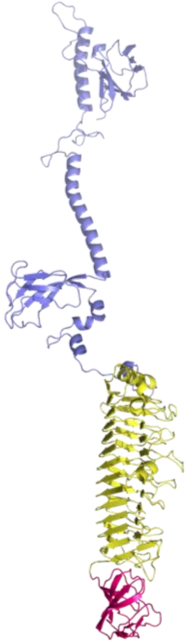	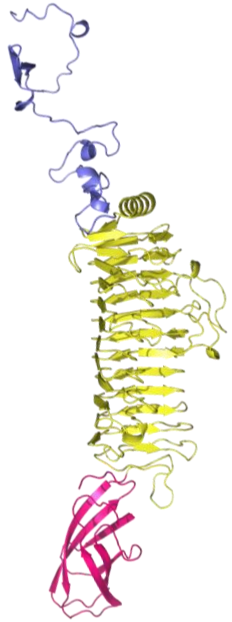

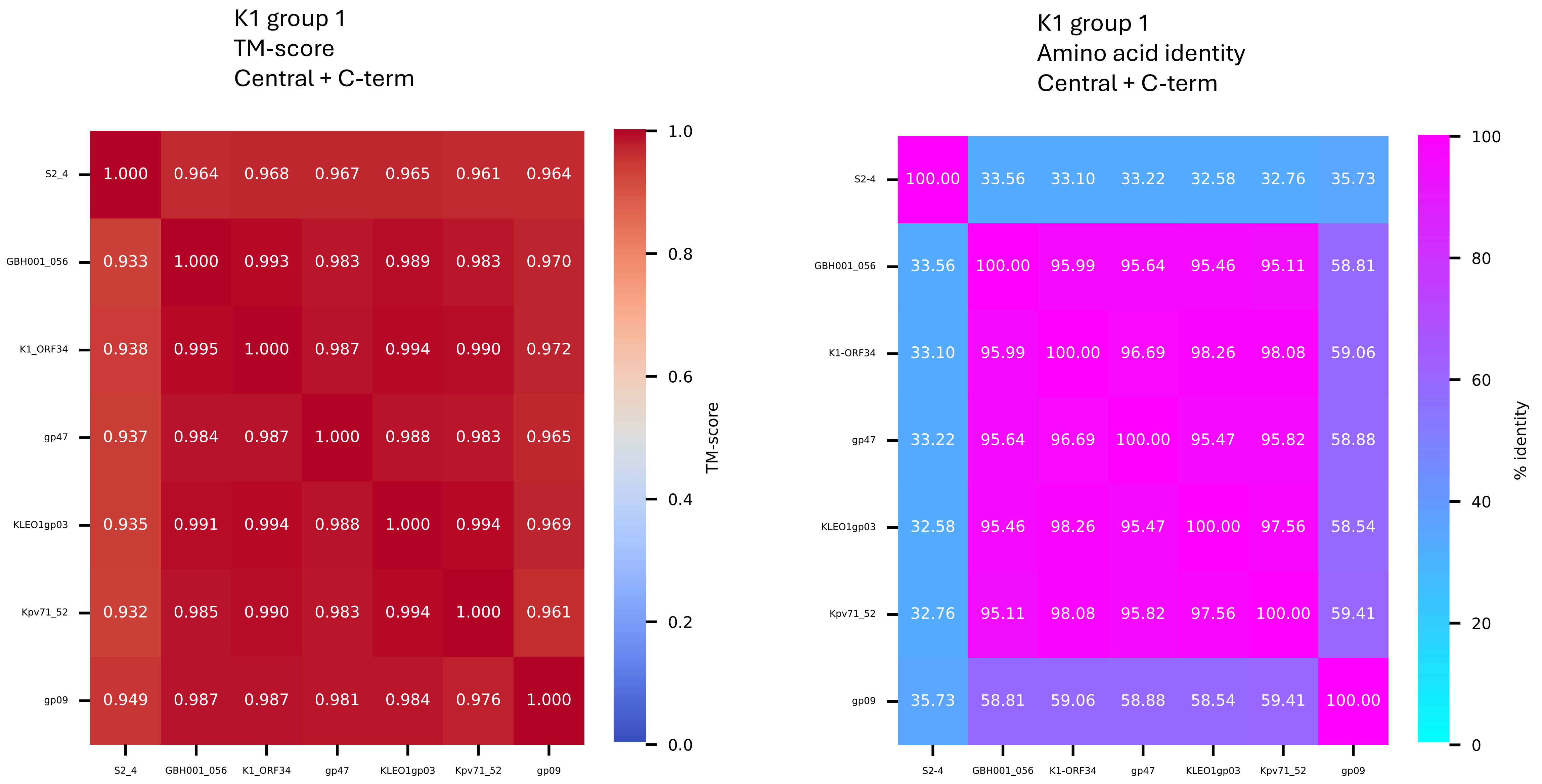

Supplementary Figure 3. Structural similarity between depolymerases is **presented as heatmaps** based on **pairwise amino acid sequence similarity** and **TM-score values** calculated for the central β-helix and C-terminal domains or for the central domain alone. Selected structural superpositions illustrate conserved architecture within compared proteins.

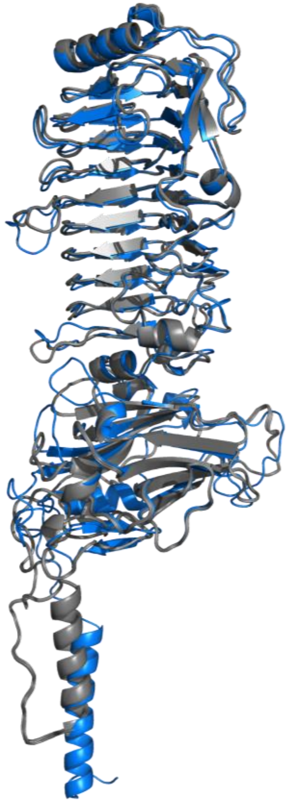

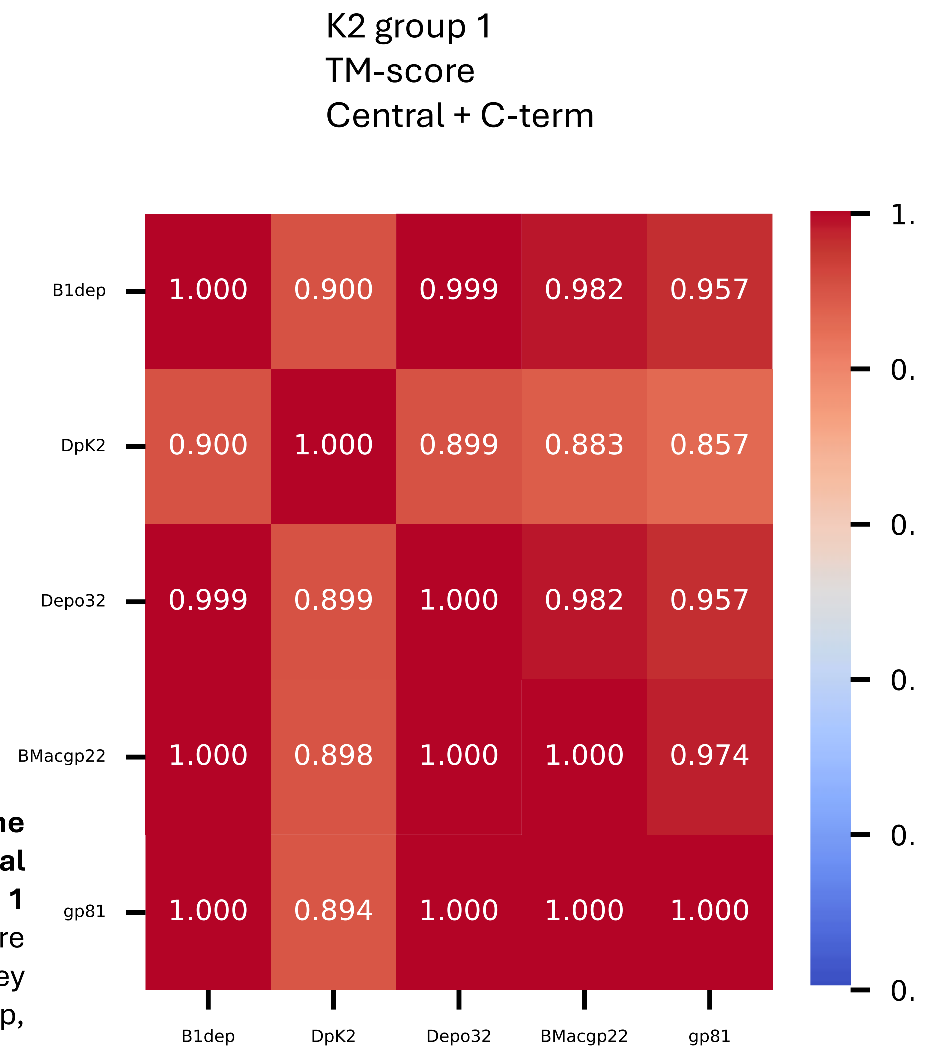

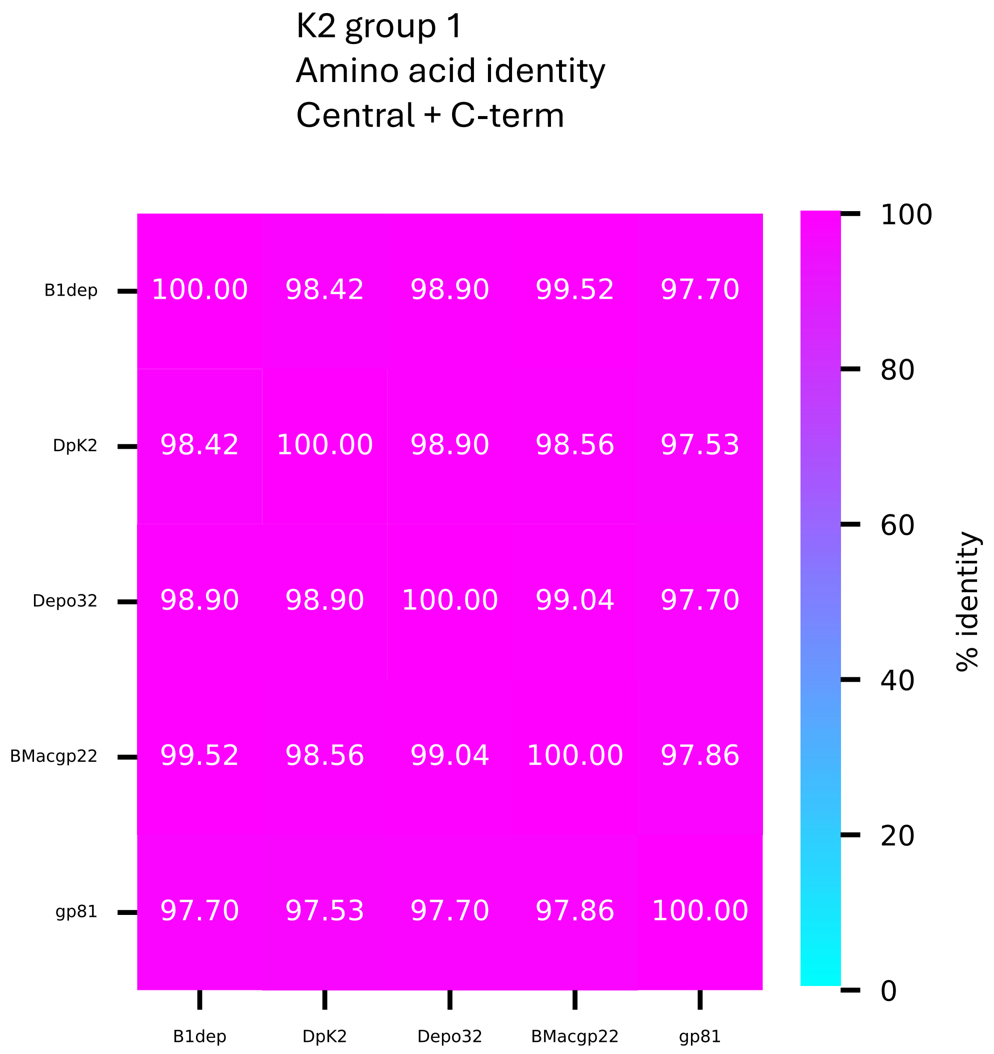

Structural comparison of the central β-helix and C-terminal domains within the K2 group 1 depolymerases. The blue structure represents DpK2, while the grey structures correspond to B1dep, BMacgp22, gp81, and Depo32.

Supplementary Figure 3. continued

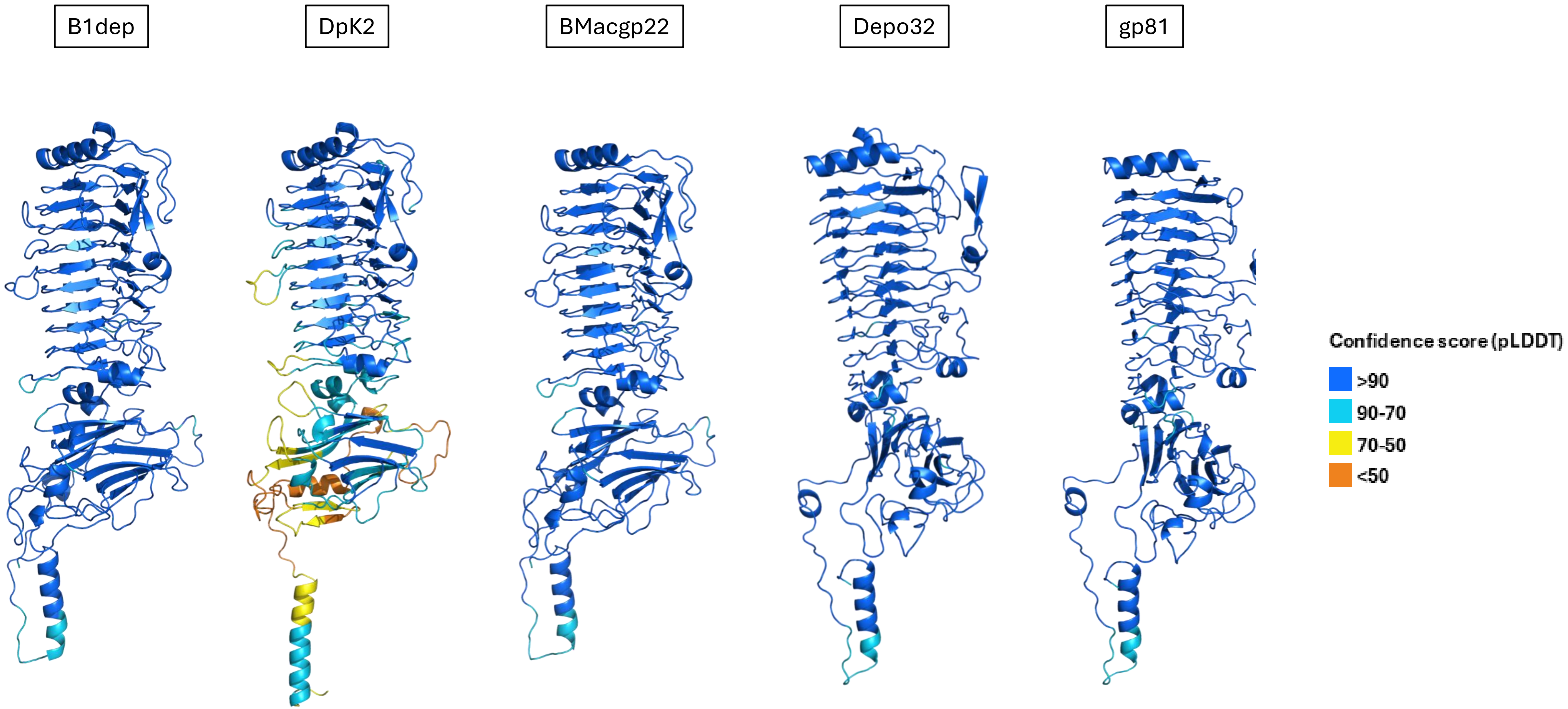

pLDDT colored structural models of B1dep, DpK2, BMacgp22, Depo32 and gp81. The difference in confidence scores of the DpK2 structural model prediction may be the reason for its lowered TM-score value in the structural comparison with other proteins from this group

Supplementary Figure 3. continued

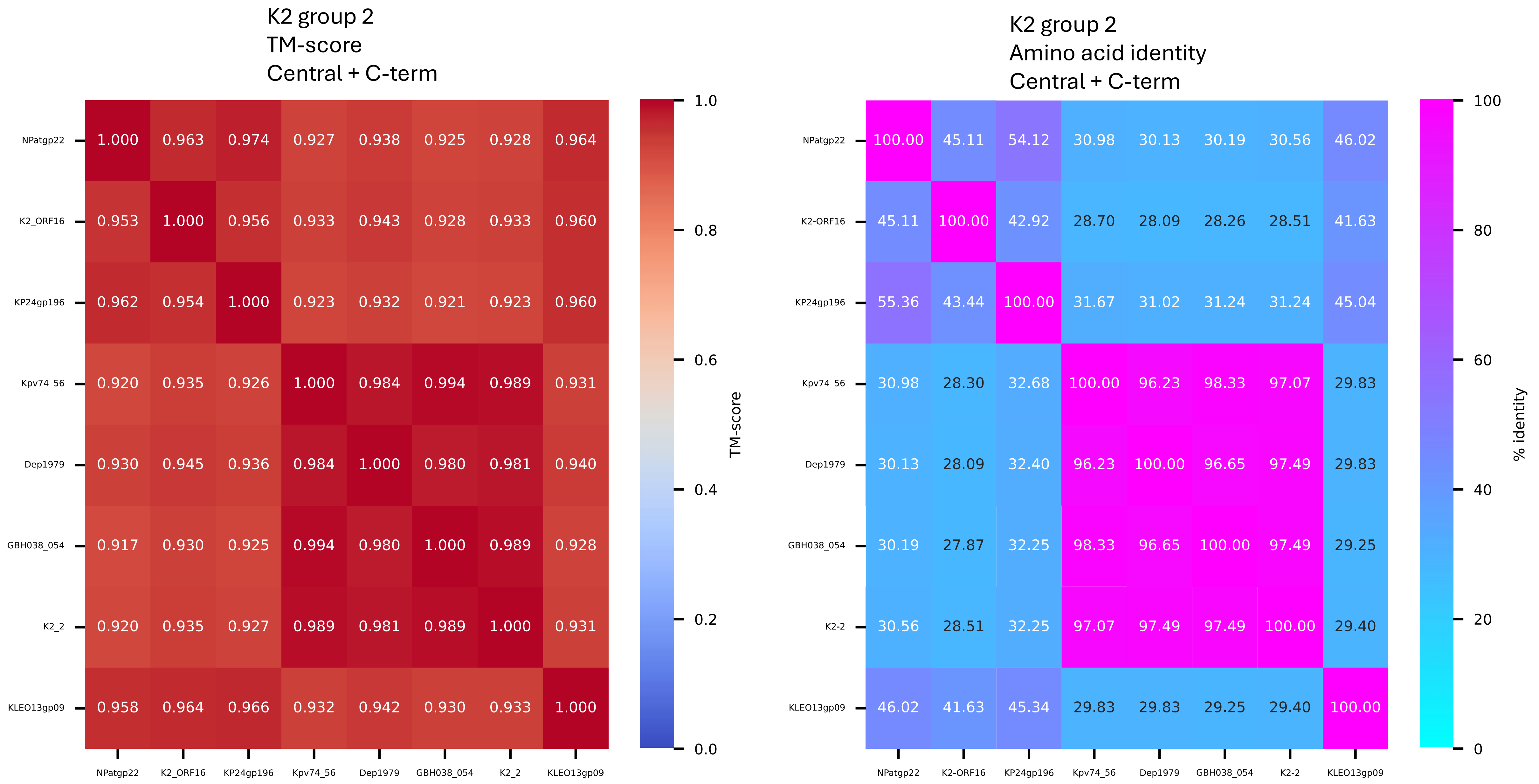

Supplementary Figure 3. continued

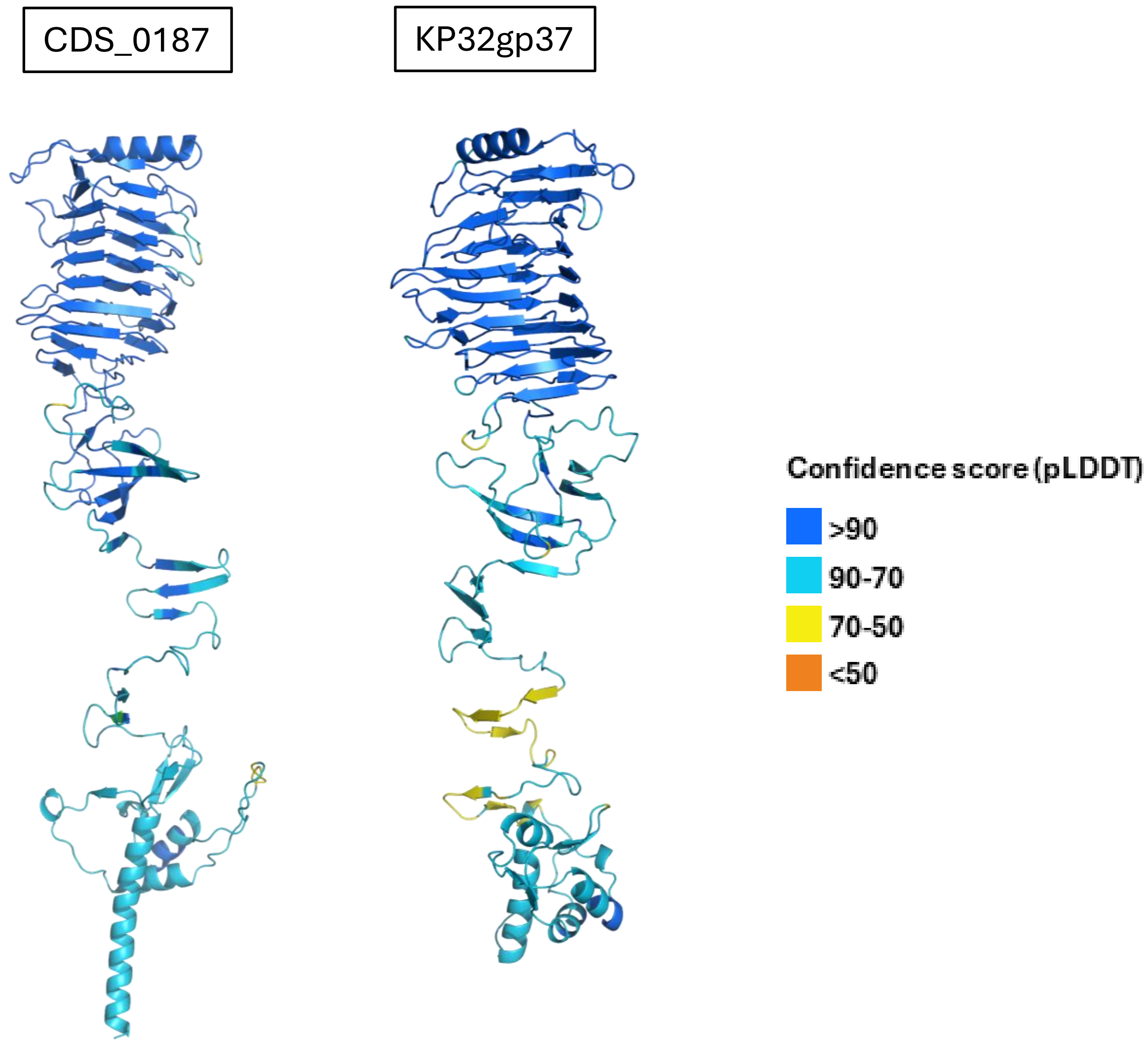

pLDDT colored structural models of CDS_0187 and KP32gp37

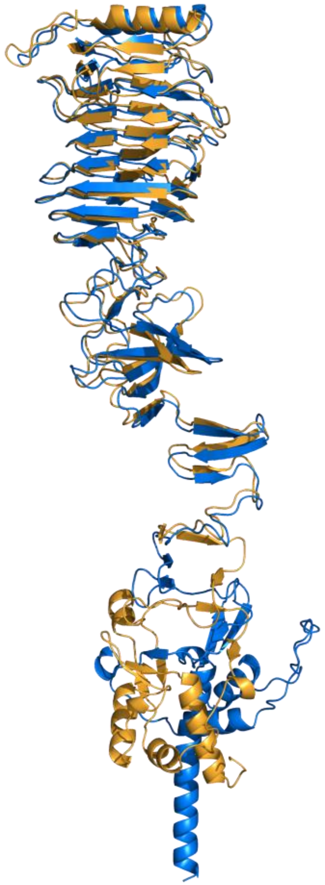

Structural comparison of the central β-helix and C-terminal domains within the K3 group 1 depolymerases. The blue structure represents CDS_0187, while the orange structure represents KP32gp37.

Supplementary Figure 3. continued

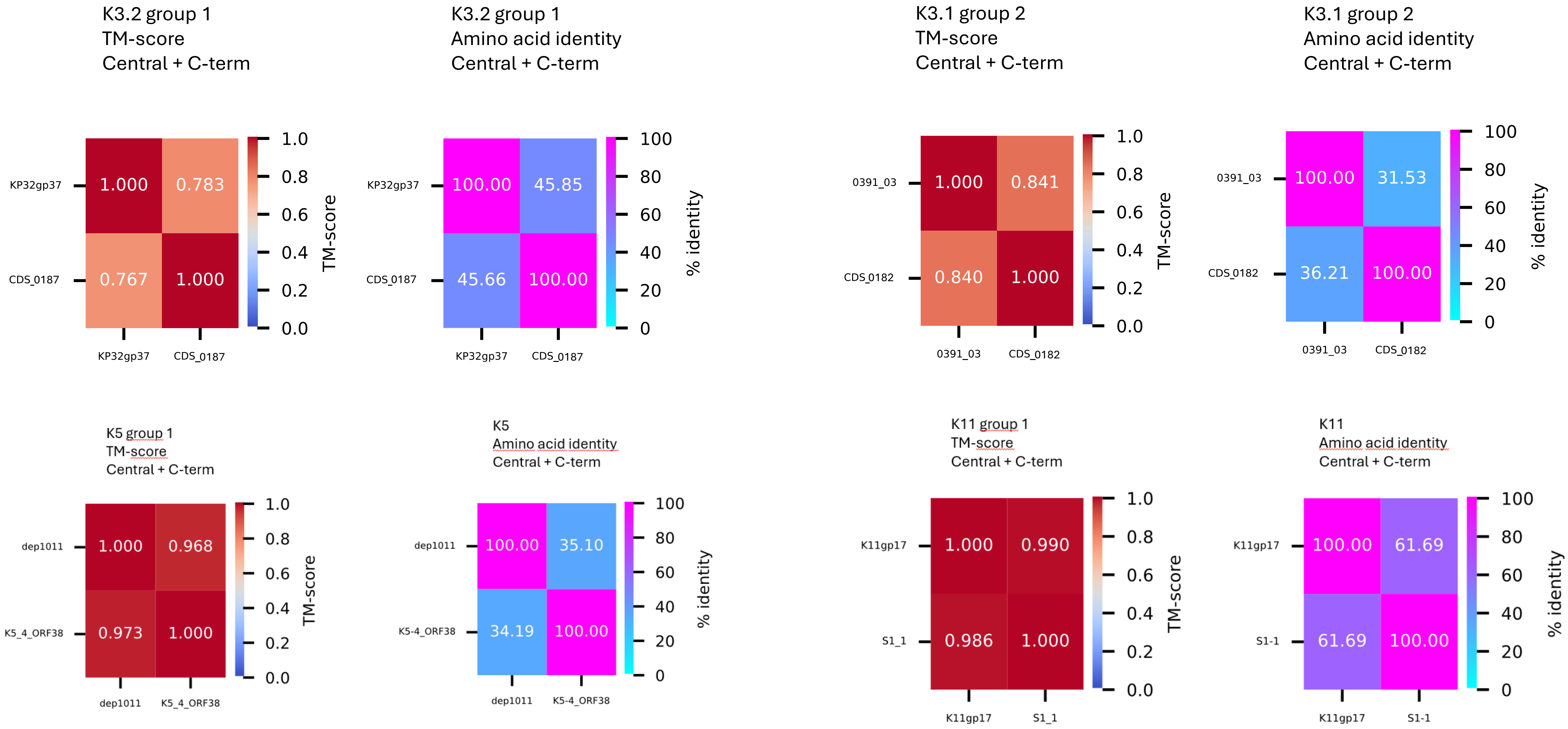

Supplementary Figure 3. continued

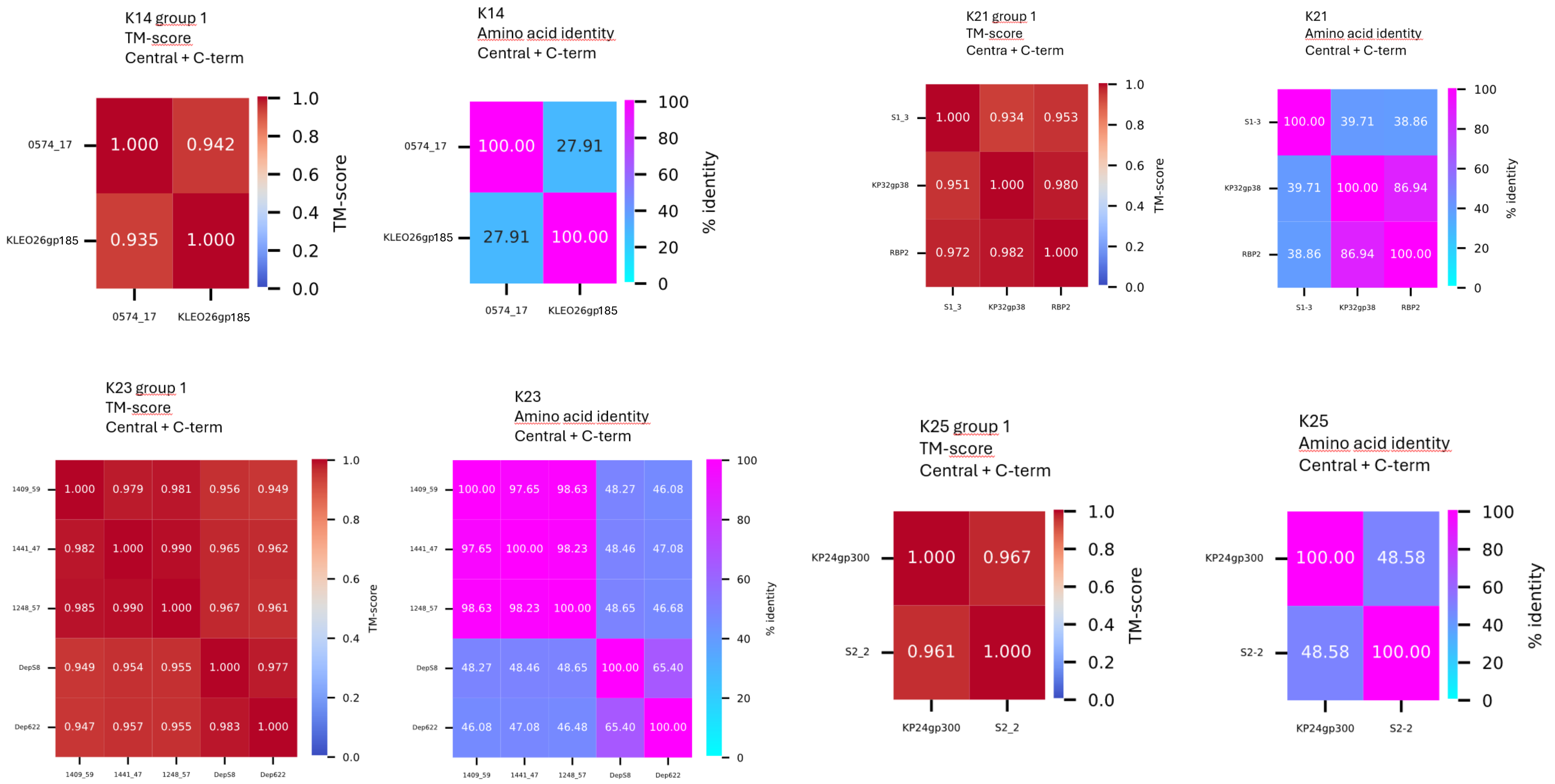

Supplementary Figure 3. continued

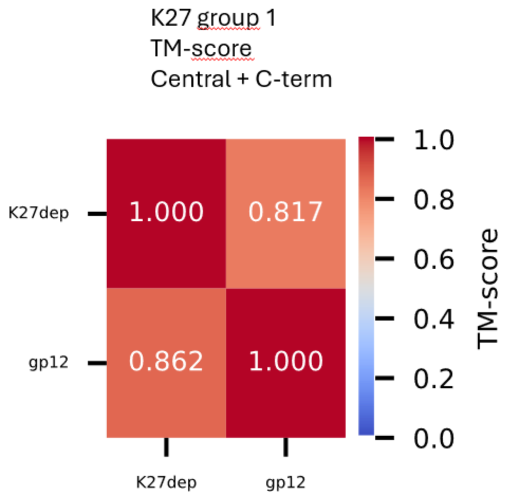

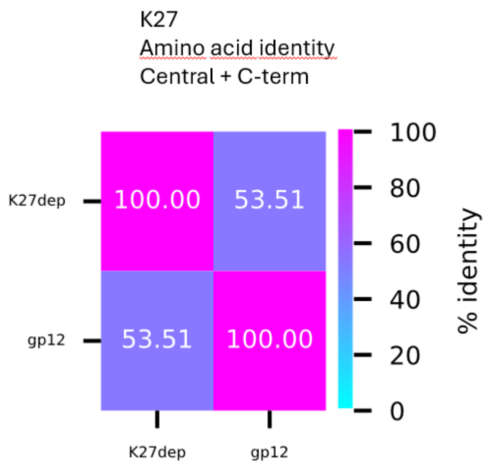

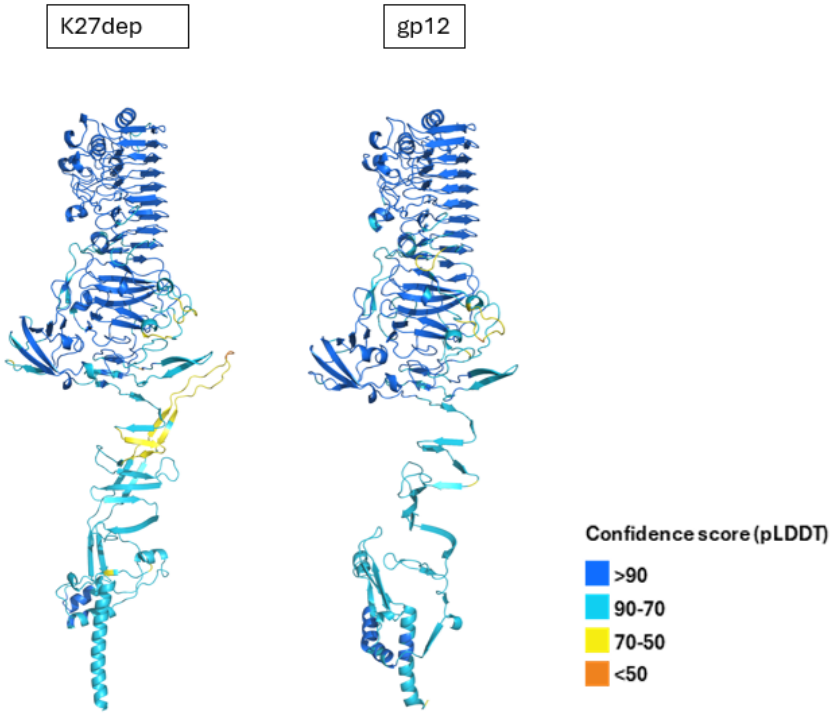

pLDDT colored structural models of K27dep and gp12. Lower confidence value in predicted structural model of K27dep in chaperone region of C-terminal damain and its overall different architecture may be one of the reason for TM-score differences in structural comparison.

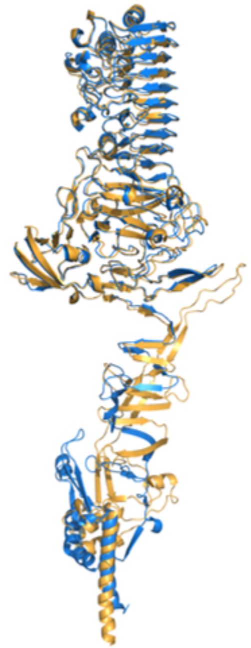

Structural comparison of the central β-helix and C-terminal domains within the K27 group 1 depolymerases. The blue structure represents K27dep, while the orange represents gp12.

Supplementary Figure 3. continued

K30_69 group 1
TM-score
Central + C-term

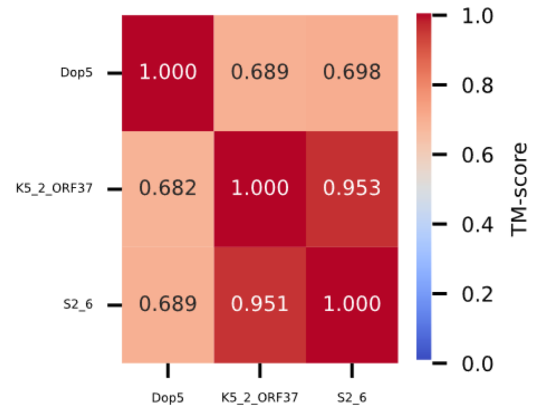

K30
Amino acid identity
Central + C-term

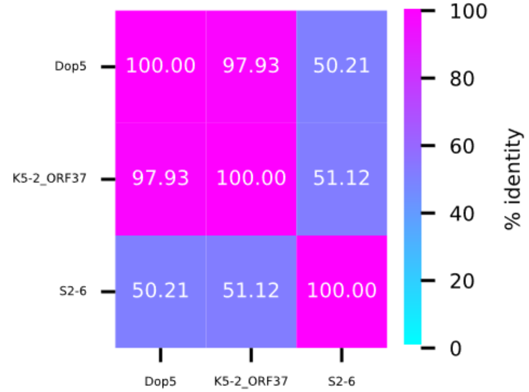

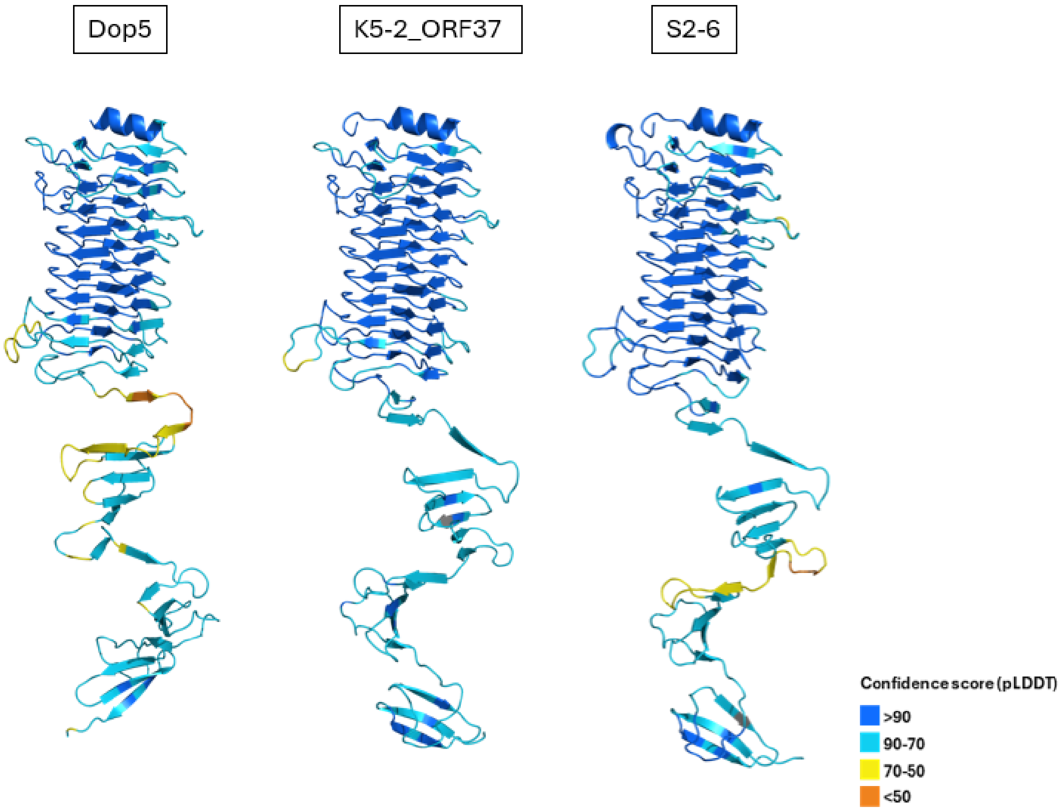

pLDDT colored structural models of Dop5, K5-2_ORF37 and S2-6. The difference in confidence scores of the Dop5 structural model prediction in the beginning of the C-terinal domain may be the reason for its lowered TM-score value in the structural comparison with other proteins from this group

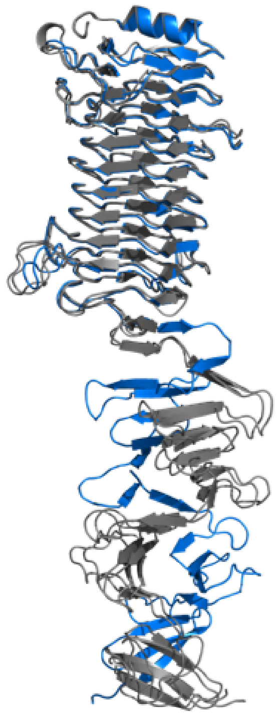

Structural comparison of the central β-helix and C-terminal domains within the K30-K69 group 1 depolymerases. The blue structure represents Dop5, while the gray represent K5-2_ORF37 and S2-6

Supplementary Figure 3. continued

K35 group 1
TM-score
Central + C-term

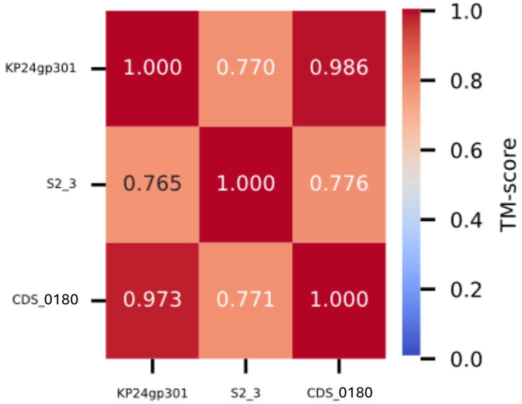

K35 group 1
Amino acid identity
Central + C-term

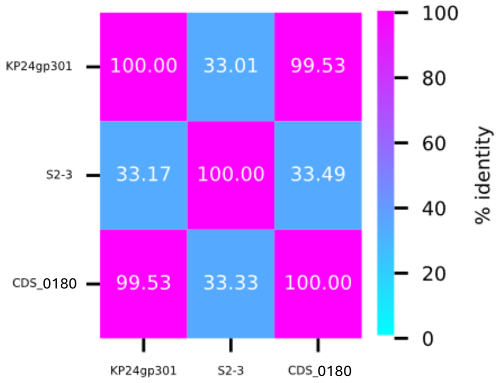

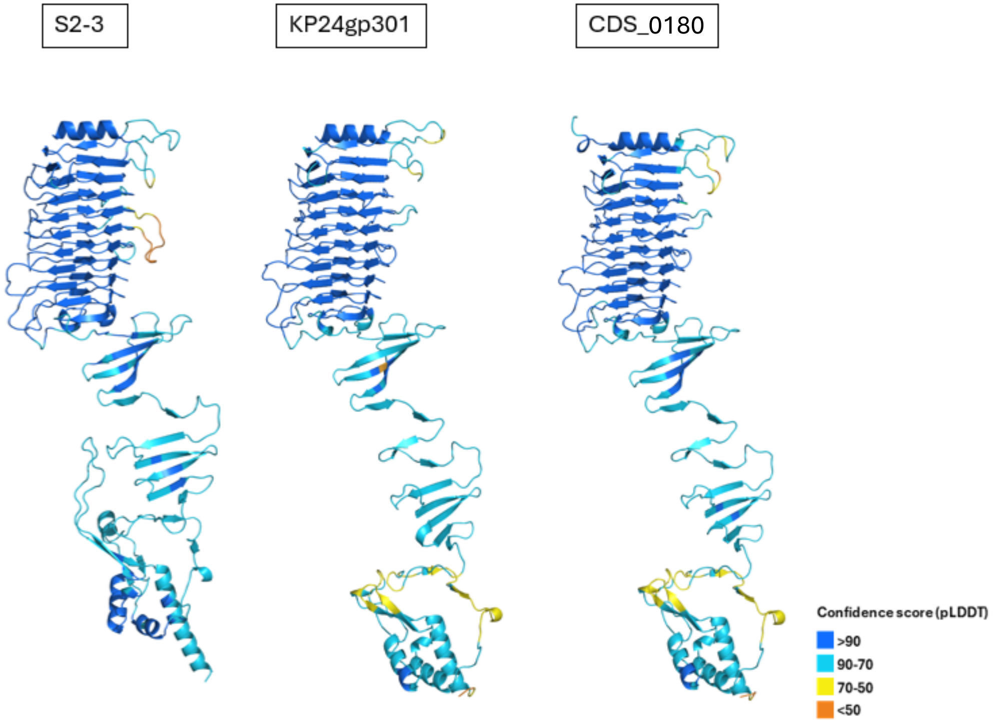

pLDDT colored structural models of S2-3, KP24gp301 and CDS_0180. Better confidence score of S2-3 predicted structural model in chaperon region in C-terminal domain may be the reason for its lower TM-score value in comparison to KP24gp301 and CDS_0180 structural models. In S2-3 structural model there is also a standalone loop with low pLDDT value which can also account for lower overall TM-score in structural comparison.

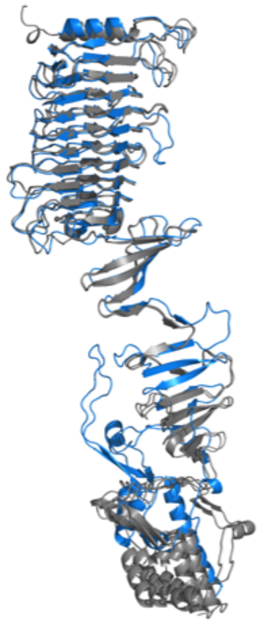

Structural comparison of the central β-helix and C-terminal domains within the K35 group 1 depolymerases. The blue structure represents S2-3, while the gray represent KP24gp301 and CDS_0180

Supplementary Figure 3. continued

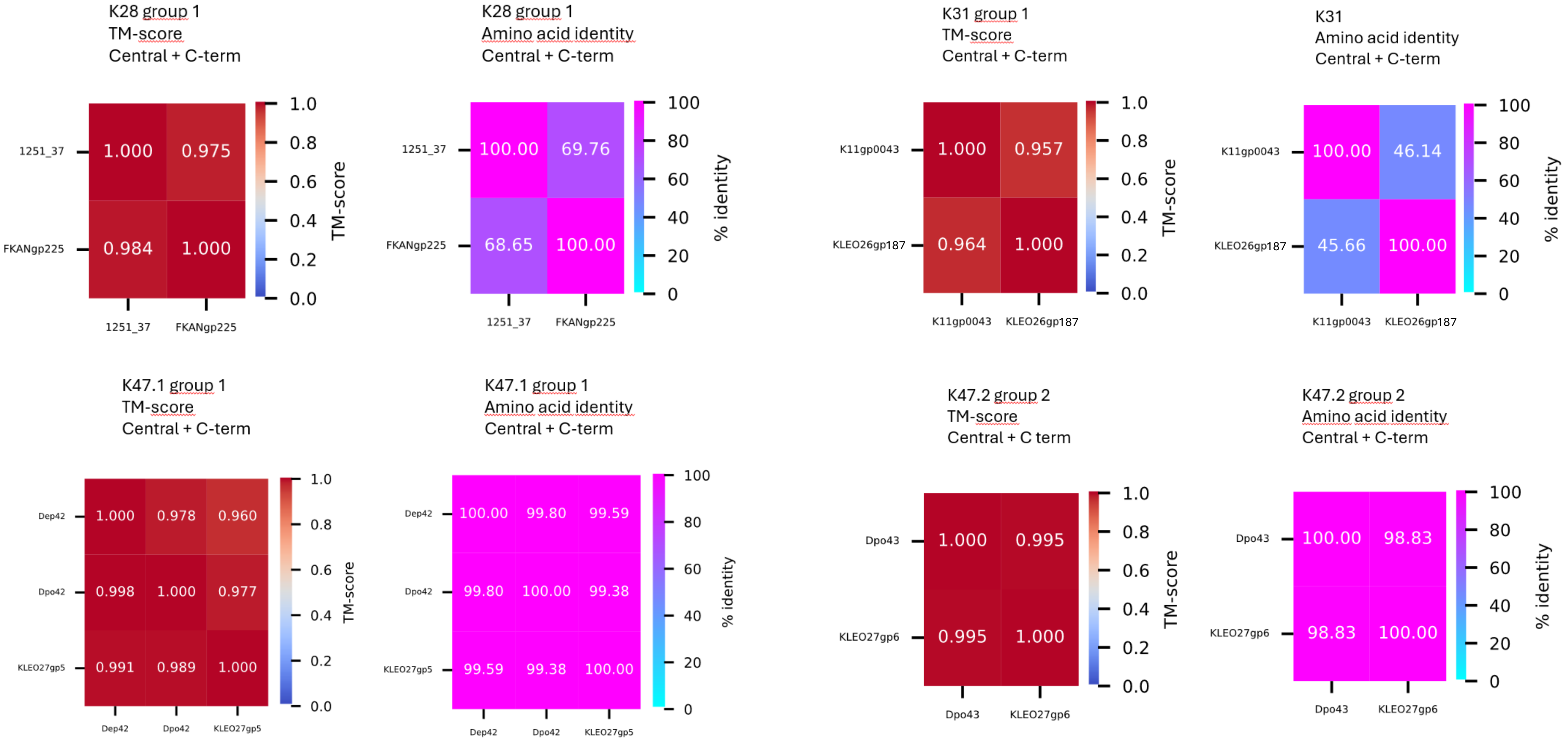

Supplementary Figure 3. continued

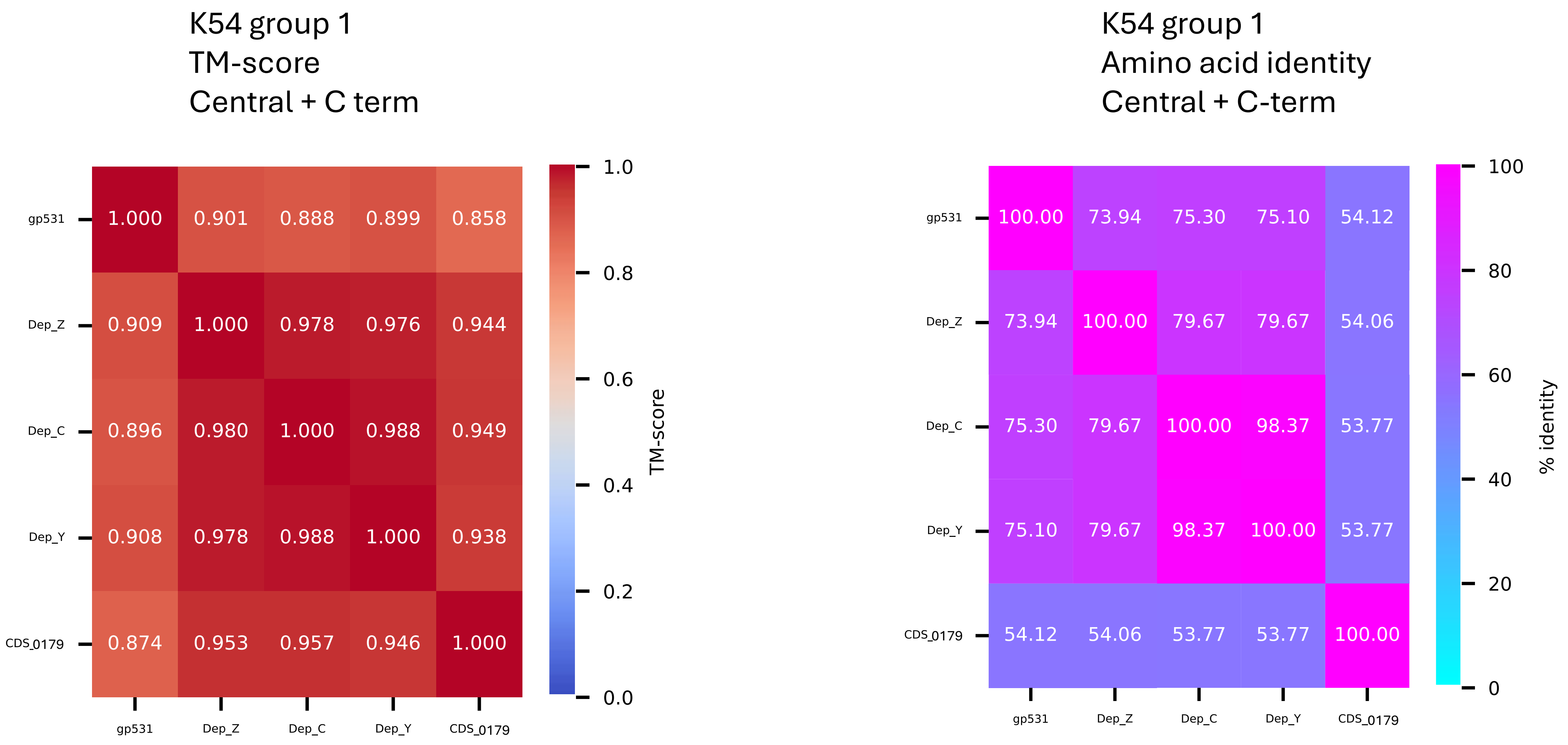

Supplementary Figure 3. continued

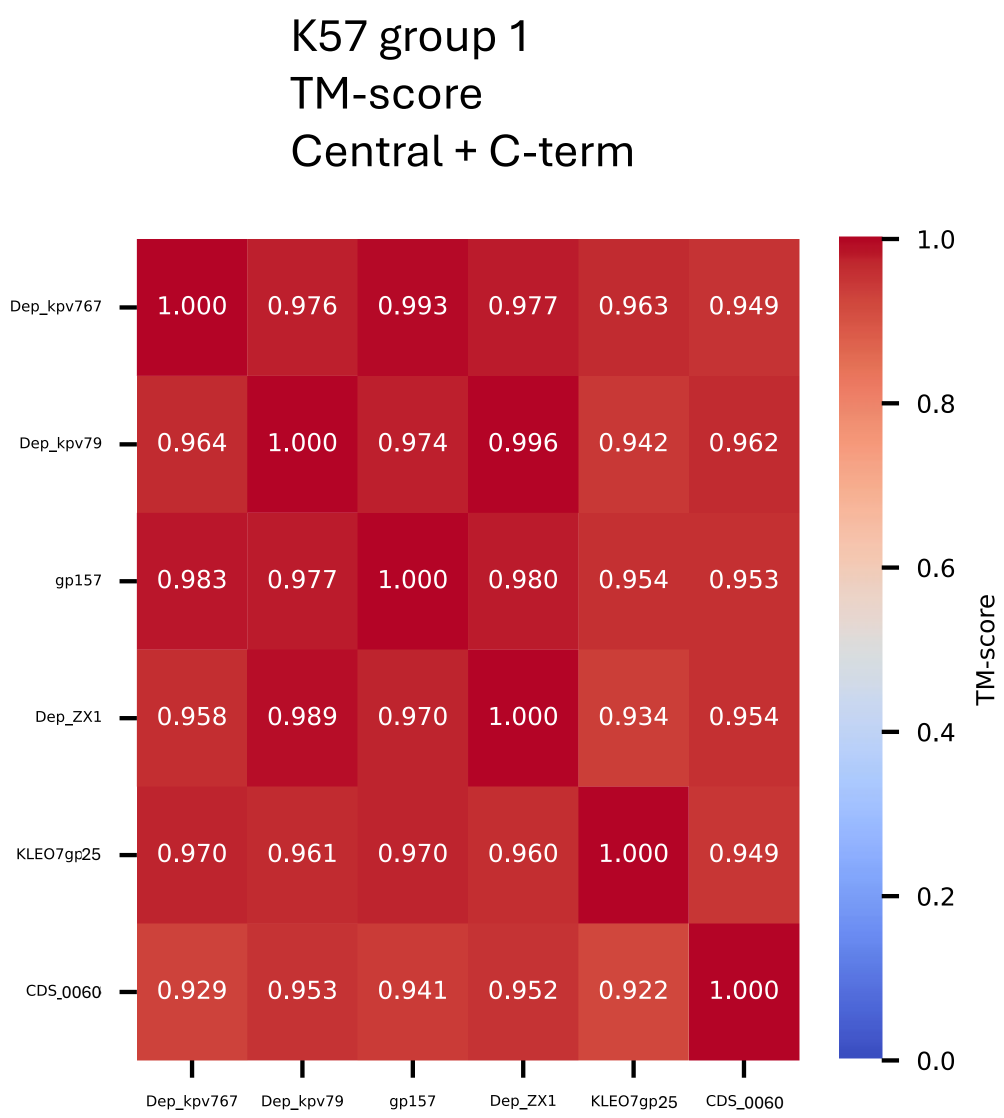

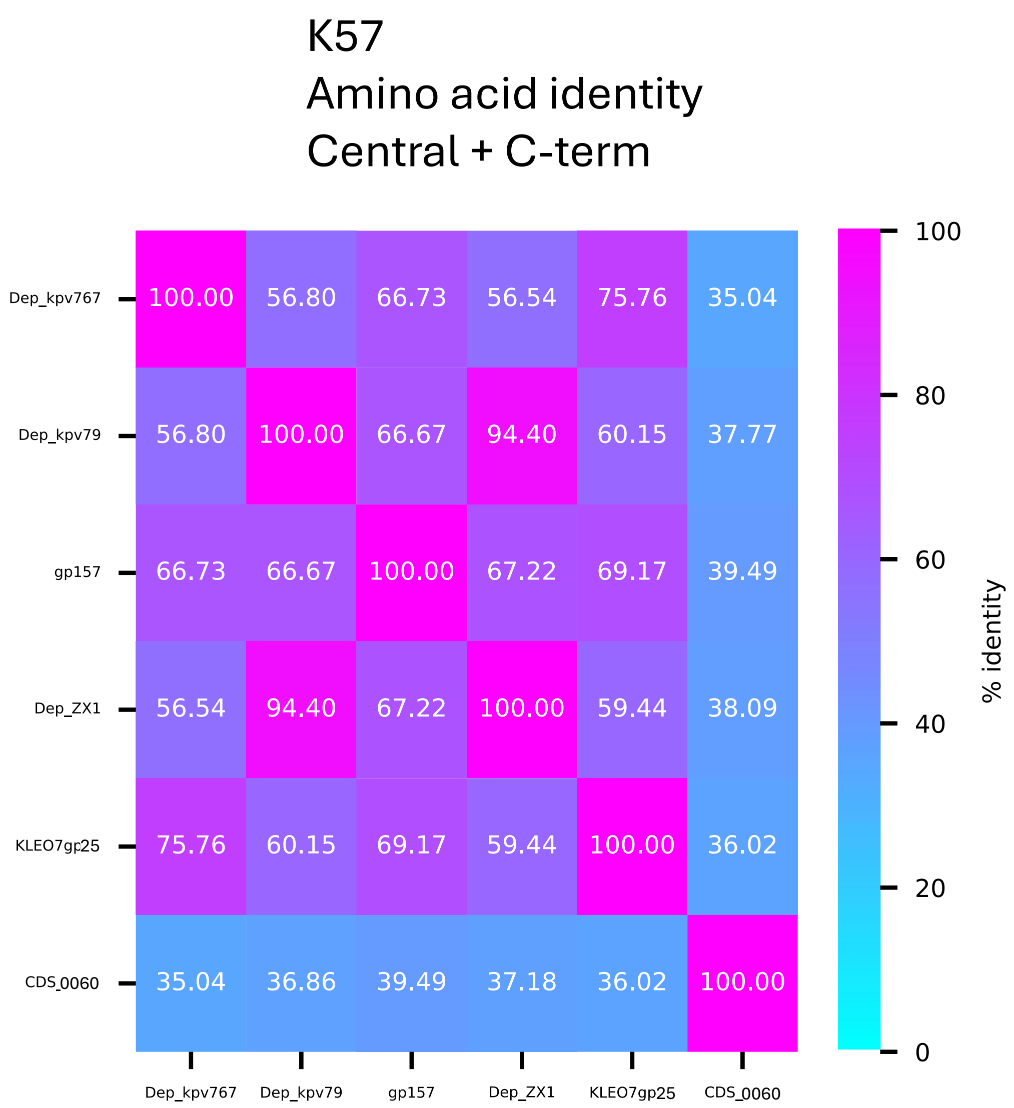

Supplementary Figure 3. continued

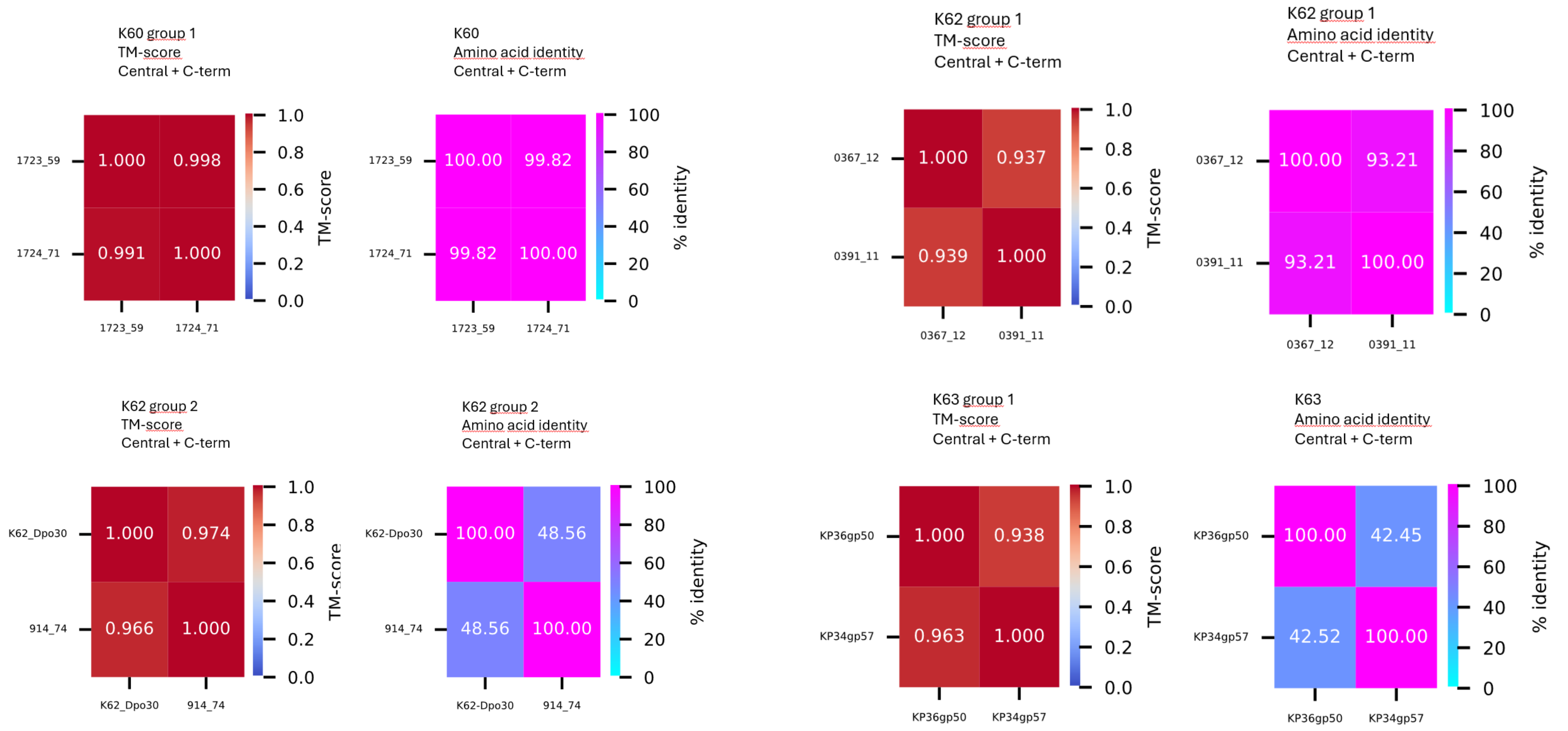

Supplementary Figure 3. continued

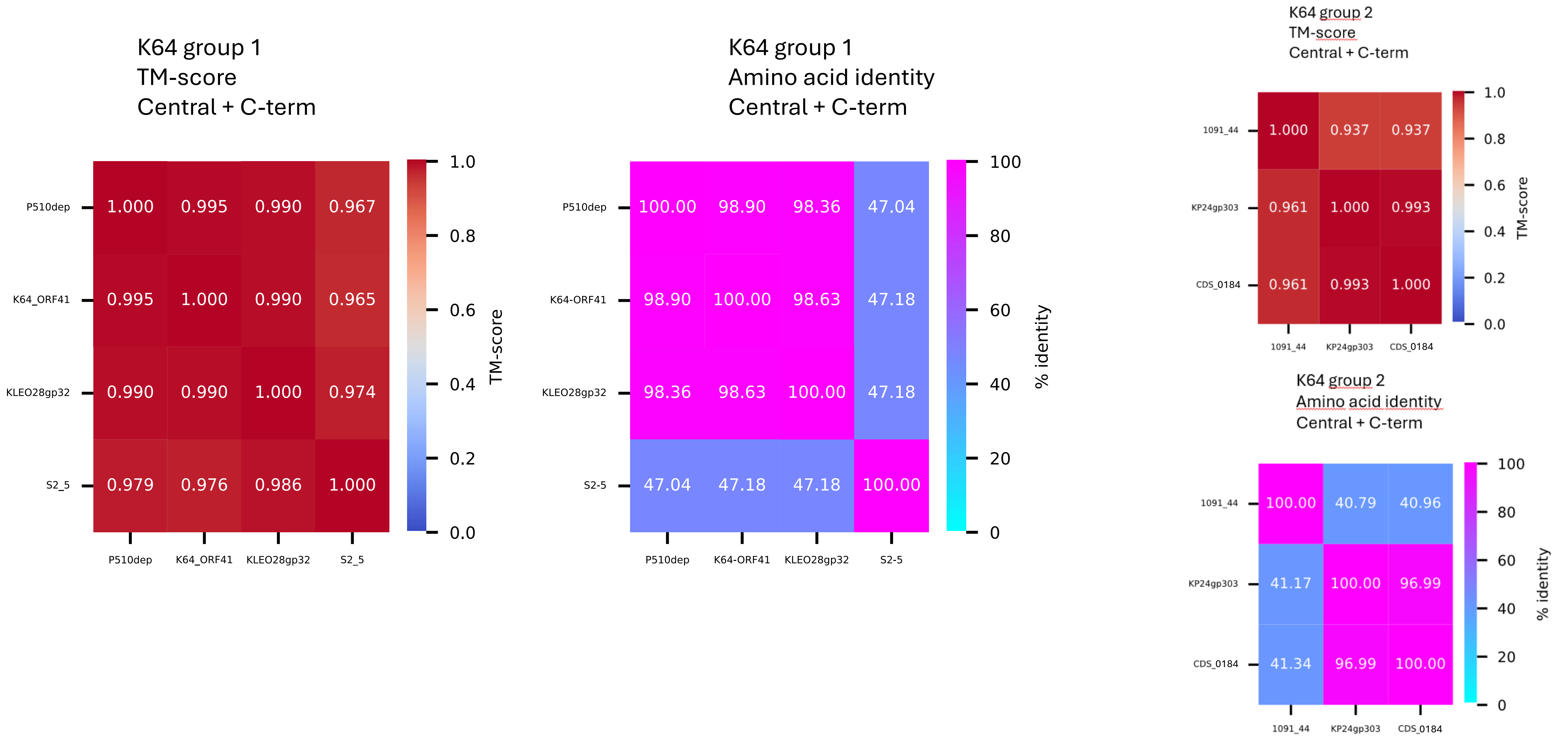

Supplementary Figure 3. continued

KN1_KL101 group 1
TM-score
Central + C-term

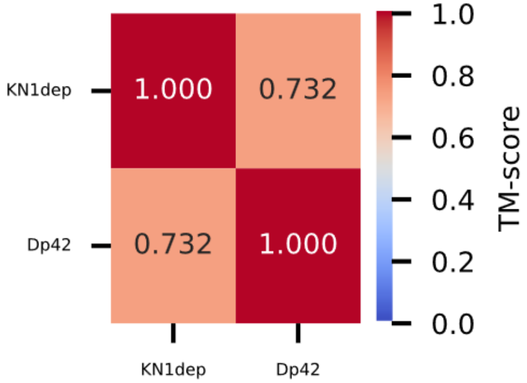

KL101
Amino acid identity
Central + C-term

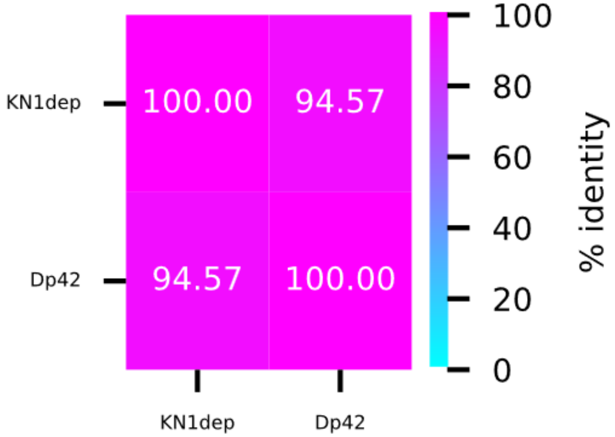

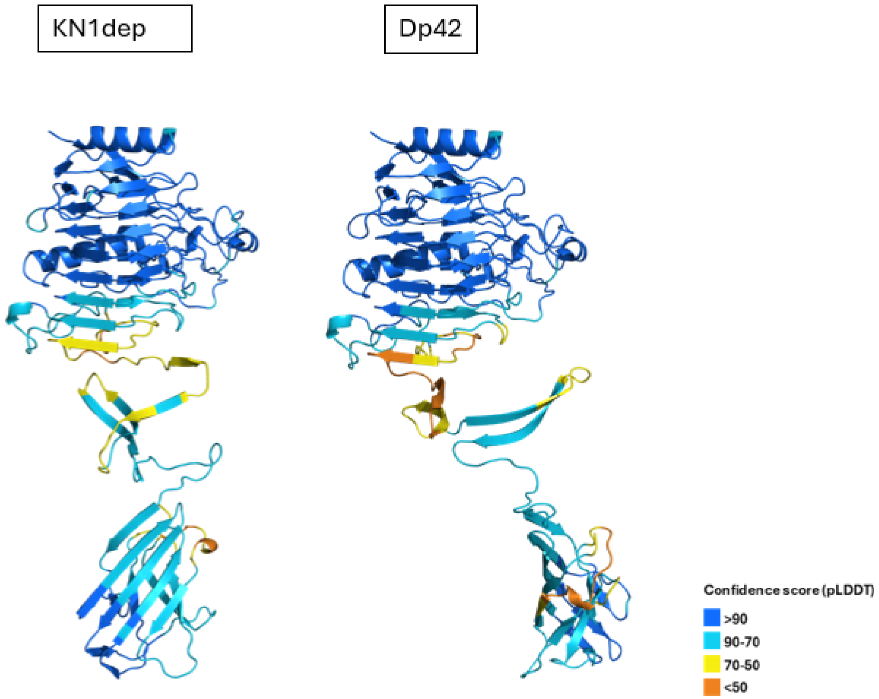

pLDDT colored structural models of KN1dep and Dp42. Lower confidence scores in structural models of both proteins may be one of the reason for TM-score differences in structural comparisons.

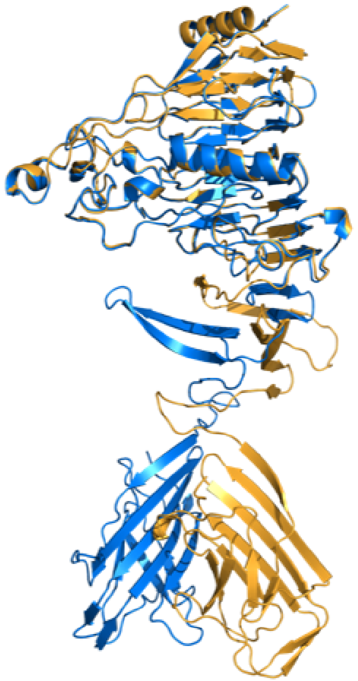

Structural comparison of the central β-helix and C-terminal domains within the KL101-KN1 group 1 depolymerases. The blue structure represents Dp42, while the orange represents KN1dep.

Supplementary Figure 3. continued

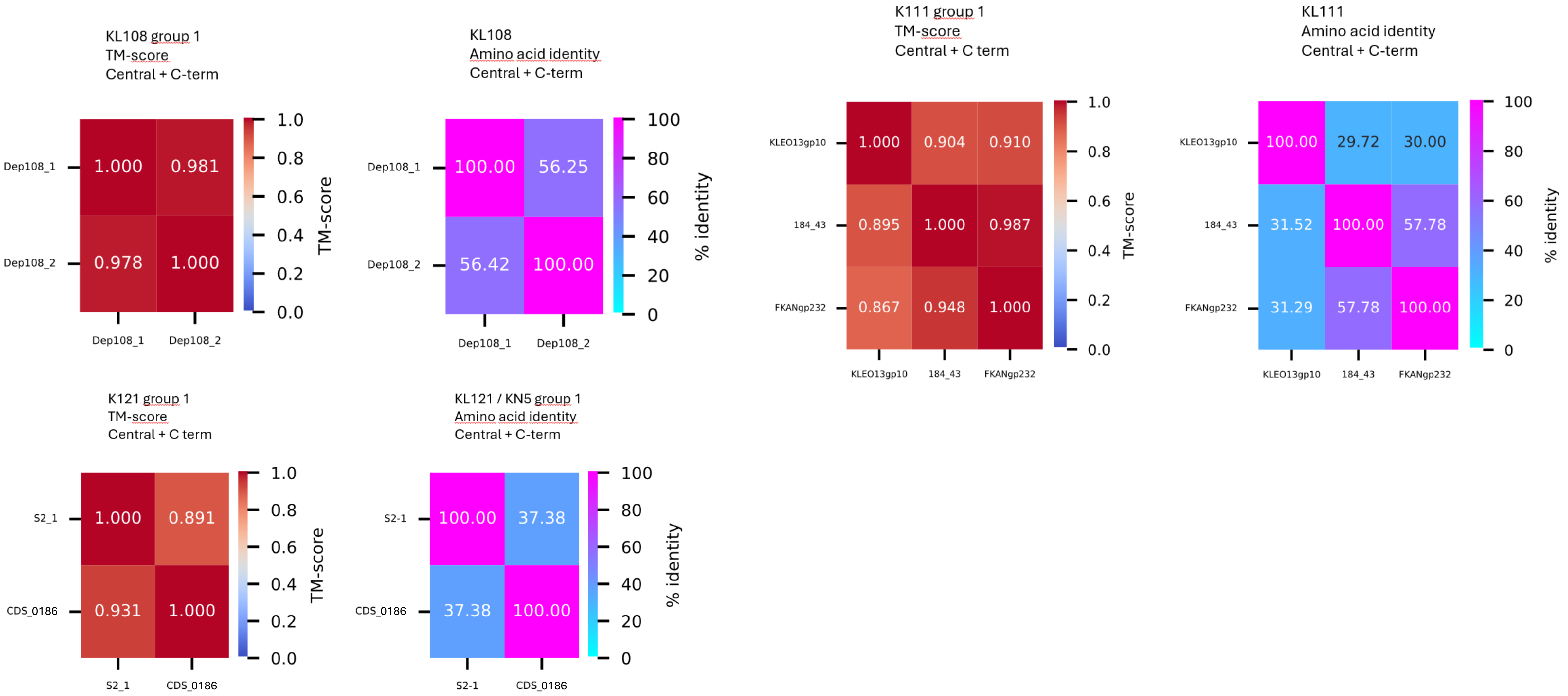

Supplementary Figure 3. continued

K124 group 1
TM-score
Central + C term

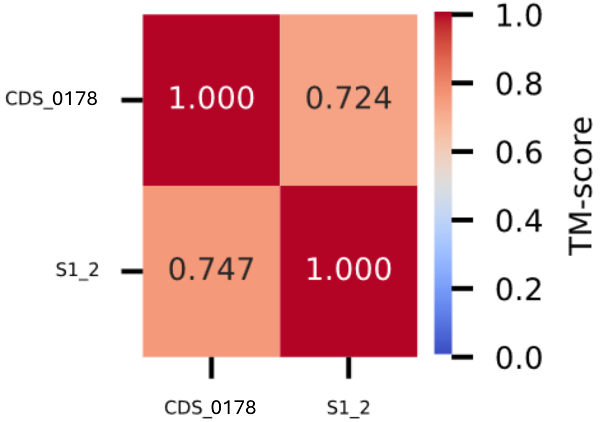

K124 / KN4 group 1
Amino acid identity
Central + C-term

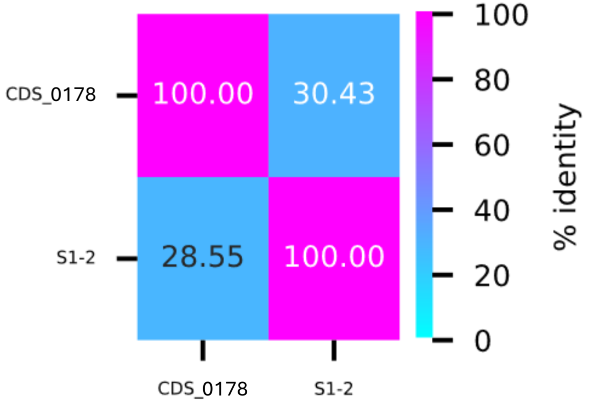

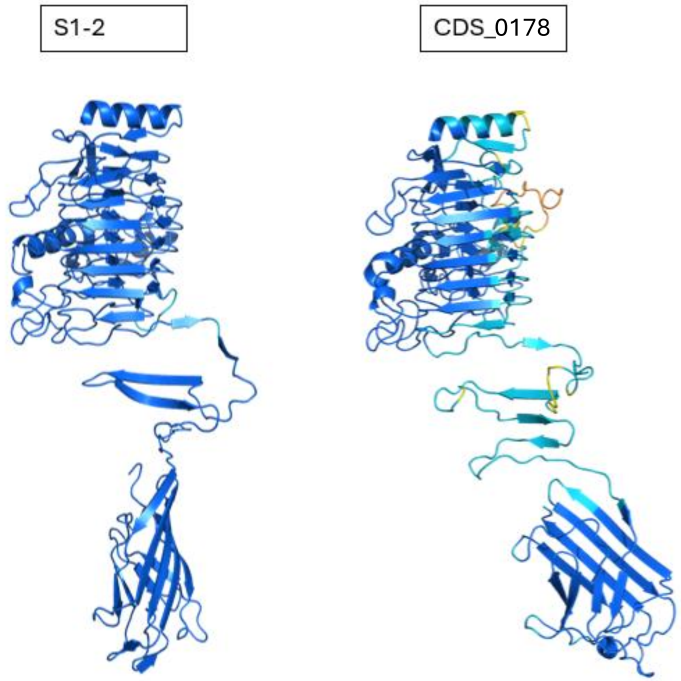

pLDDT colored structural models of S1-2 and CDS_0178

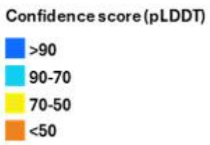

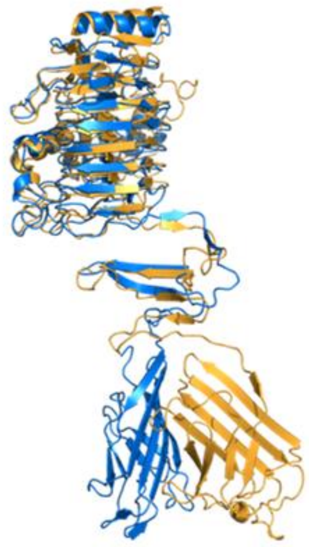

Structural comparison of the central β-helix and C-terminal domains within the KL124 group 1 depolymerases. The blue structure represents S1-2, while the orange represents CDS_0178. The differences in the structural arrangements in C-terminal domain is the main reason for TM-score differences.

Supplementary Figure 3. continued

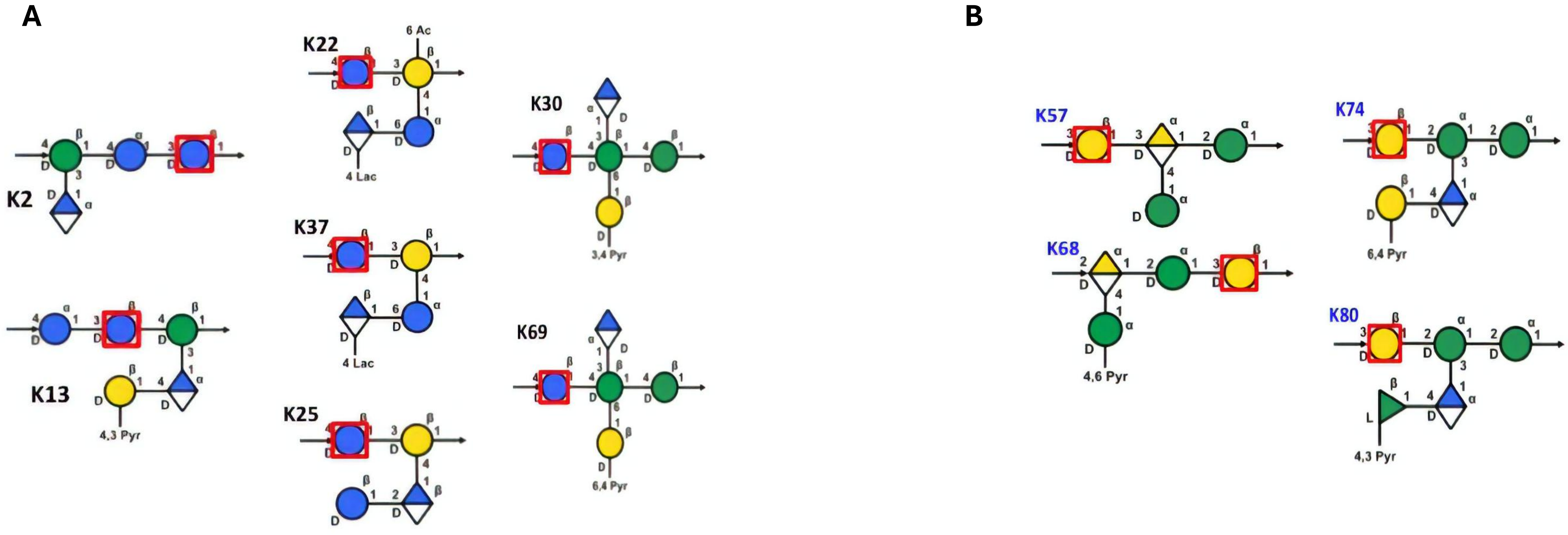

Supplementary Figure 4. Schematic comparison of the glycan structures of several K-antigens recognized in pairs by some of the depolymerases grouped into: **A**. glucose as the first monomer category **B**. galactose as the first monomer category. The diagrams illustrate the monosaccharide composition, linkage positions, and overall structural organization of each antigen, including branching patterns and ring types. CPS structures were obtained from the K-PAM resource (*K-PAM: Klebsiella Pneumoniae Antigen Typing Resource – K Antigen*. Available at: https://iith.ac.in/K-PAM/k_antigen.html (Accessed: 15 March 2026)[1].

Supplementary Figure 5. Depolymerase catalog presented as a comparison of representative depolymerases from distinct classes and subclasses. The tables summarize subclass distinctions, targeted K-types, polysaccharide subunit compositions, and protein lengths for each enzyme. Predicted 3D protein structures generated with AlphaFold3.0 are shown below each entry, illustrating domain architecture and overall structural differences across subclasses.

Supplementary Figure 5. continued

Class name	Class 1 „Classical depolymerase”												
Subclass	A „Vertical stand”									B „Pouf-like”		
Protein name	FKANgp229		KLEO25gp59	DepS8	KLEO7gp25		914_74	CDS_0186	FKANgp220	P560dep	KLEO13gp09		K11gp17
K-type target	K9	KL184	K10	K23	K57	K68	K62	KL121 (KN5)	KL148	KL169	K2	K13	K11
Polysaccharide subunit composisition	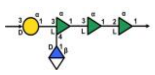	NC	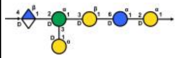	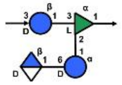	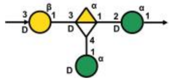	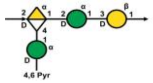	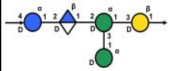	NC	NC	NC	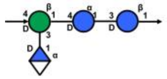	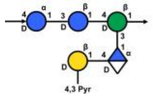	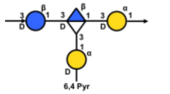
Protein length	578 aa		513 aa	607 aa	570 aa		666 aa	700 aa	591 aa	802 aa	524 aa		875 aa
Predicted protein structure (AlphaFold3)	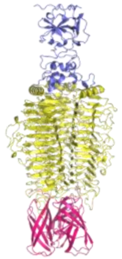		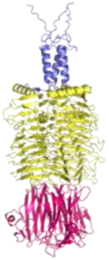	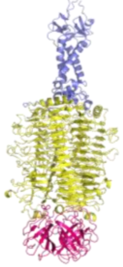	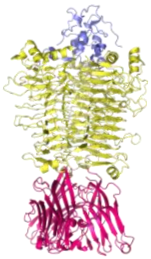		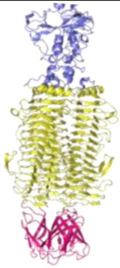	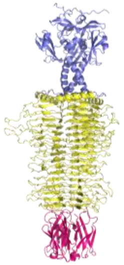	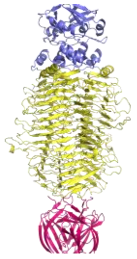	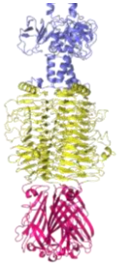	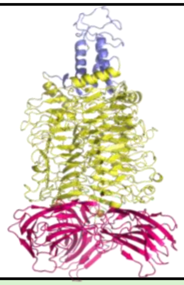		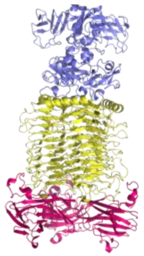

Class name	Class 1 „Classical depolymerase”												
Subclass	B „Pouf-like”												
Protein name	K11gp17	K20dep		KLEO13gp10			184_43	FKANgp232	KP24gp300	1724_71	KP24gp310	FKANgp217	KN3dep
K-type target	KL181	K20	KL132	K22	K37	KL111	KL111	KL111	K25	K60	K61	KL153	KL183 (KN3)
Polysaccharide subunit composisition	NC	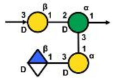	NC	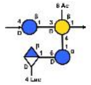	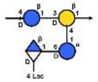	NC	NC	NC	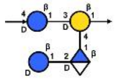	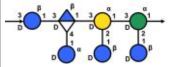	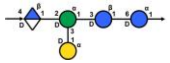	NC	NC
Protein length	875 aa	732 aa		777 aa			639 aa	623 aa	598 aa	843 aa	679 aa	644 aa	792 aa
Predicted protein structure (AlphaFold3)	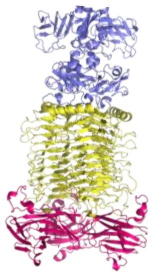	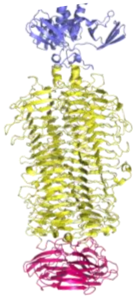		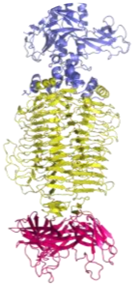			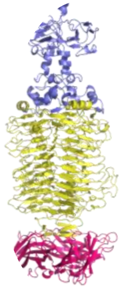	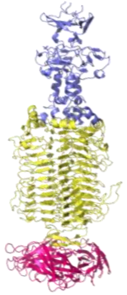	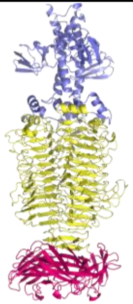	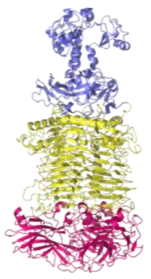	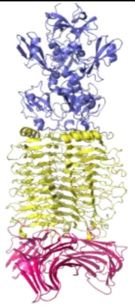	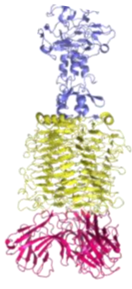	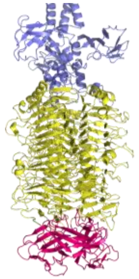

Supplementary Figure 5. continued

Class name	Class 1 „Classical depolymerase”													
Subclass	C „Spout-like”										D „Tripod”	E „Short C-domain”		
Protein name	0391_03	CDS_0182			KP24gp304	FKANgp225	K56dep	KP24gp303		KP24gp306	KLEO27gp5	GBH019_279	CDS_0190_1	
K-type target	K3.1	K3.1	K26	KL173	K19	K28	K56	K64	KL178	KL137	K47.1	K51	K74	K80
Polysaccharide subunit composisition	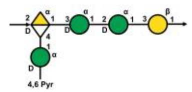		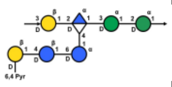	NC	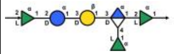	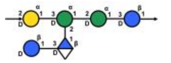	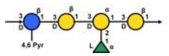	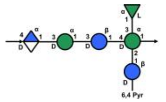	NC	NC	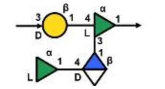	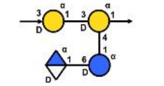	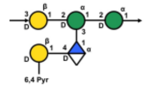	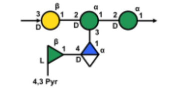
Protein length	736 aa	598 aa			755 aa	605 aa	678 aa	661 aa		883 aa	793 aa	809 aa	690 aa	
Predicted protein structure (AlphaFold3)	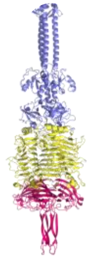	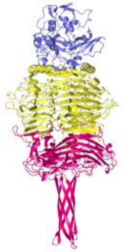			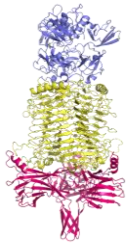	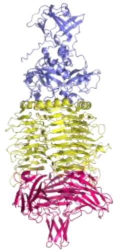	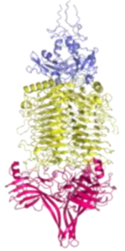	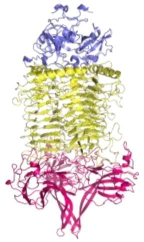		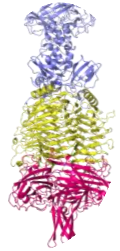	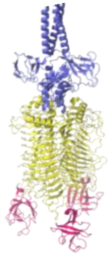	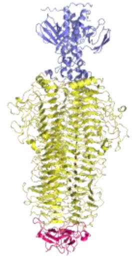	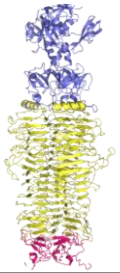	

Class name	Class 1 „Classical depolymerase”													
Subclass	E „Short C-domain”				F „Double C-domain”									
Protein name	KN4dep	KP24gp168	738_68	KP24gp309	gp81		KP32gp38		KLEO26gp187	248_38	gp531	319_37	CDS_0191	CDS_0185
K-type target	KL124 (KN4)	KL134	KL143	KL158	K2	K13	K21	KL163	K31	K46	K54	KL127	KL148	KL151
Polysaccharide subunit composisition	NC	NC	NC	NC	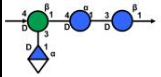	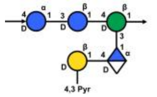	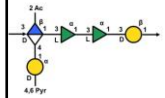	NC	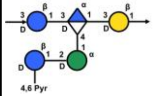	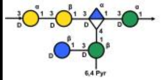	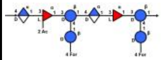	NC	NC	NC
Protein length	850 aa	881 aa	742 aa	751 aa	897 aa		576 aa		675 aa	693 aa	895 aa	794 aa	728 aa	952 aa
Predicted protein structure (AlphaFold3)	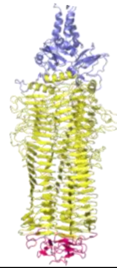	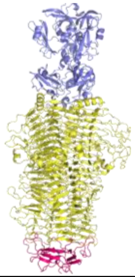	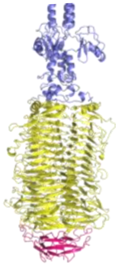	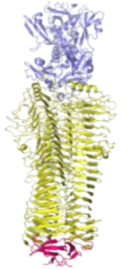	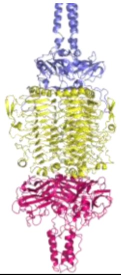		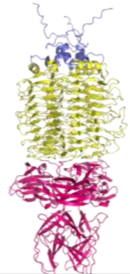		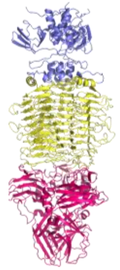	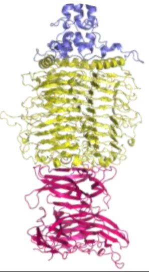	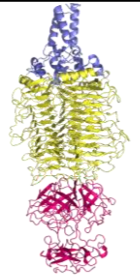	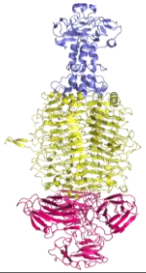	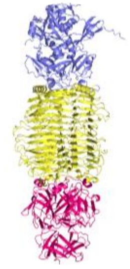	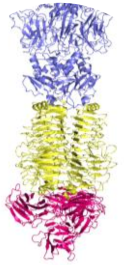

Supplementary Figure 5. continued

Class name	Class 2 „Insertion domain (ID)"										Class 3 „Tail fiber domain"		
Subclass	A „Classical insertion domain "							B „Middle ID"	C „Low-lay ID"	D „Double ID"	A „Tail fiber domain + classical C-domain"		
Protein name	KLEO1gp03	K5-4 ORF38	CDS_0189	0574_17	KP34gp57	KLEO28gp32		Kl-dep	0391_11	KLEO27gp6	K5-4 ORF37	S2-6	
K-type target	K1	K5	K6	K14	K63	K64	KL178	K20	K62	K47.2	K8	K30	K69
Polysaccharide subunit composisition	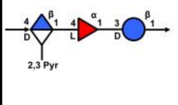	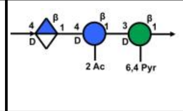	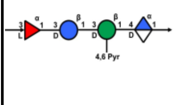	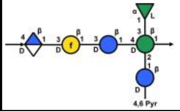	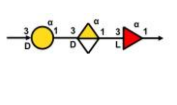	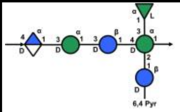	NC	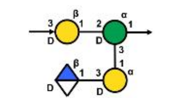	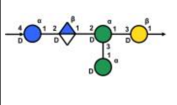	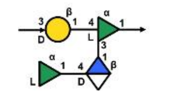	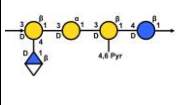	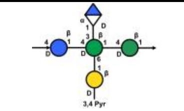	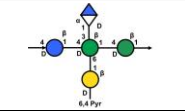
Protein length	651 aa	684 aa	874 aa	873 aa	630 aa	1017 aa		790 aa	839 aa	641 aa	749 aa	767 aa	
Predicted protein structure (AlphaFold3)	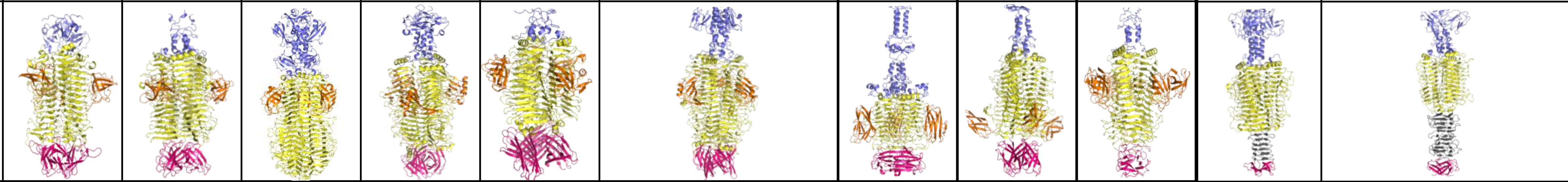												

Class name	Class 3 „Tail fiber domain"														
Subclass	A „Tail fiber domain + classical C-domain"							B „Tail fiber domain + chaperon domain"							
Protein name	914_77	KLEO26gp181	FKANgp227	KP24gp308		FKANgp223		CDS_0187		KP32gp37		K7dep	KP24gp301	434_33	0496_72
K-type target	K32	K35	K39	K46	KL146	KL110	KL116	K3.2	KL116	K3.2	KL110/KL116/KL117	K7	K35	K52	KL122
Polysaccharide subunit composisition	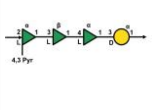	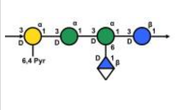	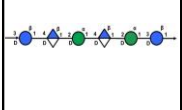	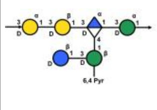	NC	NC	NC	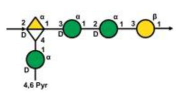	NC	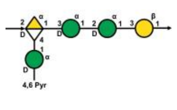	NC	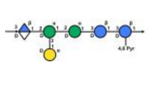	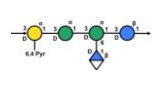	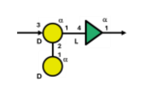	NC
Protein length	536 aa	1039 aa	820 aa	737 aa		581 aa		774 aa				1180 aa	915 aa	859 aa	968 aa
Predicted protein structure (AlphaFold3)	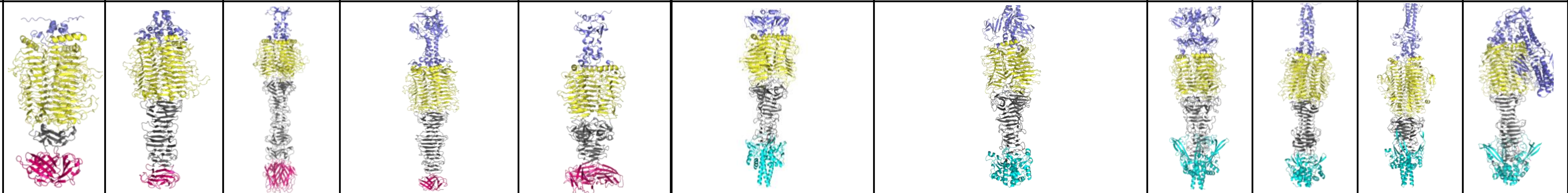														

Supplementary Figure 5. continued

Class name	Class 4 „αH-containing central domain+ (αH-CD)”							Class 5 „Colanidase-like depolymerase”	
Subclass	A „αH-CD + tail fiber domain+classical C-domain”				B „αH-CD + tail fiber domain + chaperon domain”				
Protein name	Dp42			S1-2	gp12	617_77	ORF96	Dep108.1	KP24gp307
K-type targeted	KL101 (KN1)	KL120	KL170	KL124 (KN4)	K27	K38	KL102 (KN2)	KL108	KL114
Polysaccharide subunit composisition	NC	NC	NC	NC	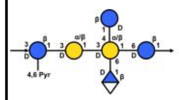	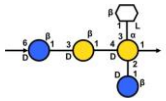	NC	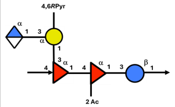	NC
Protein length	820 aa			736 aa	1242 aa	1258 aa	1245 aa	590 aa	742 aa
Predicted protein structure (AlphaFold3)	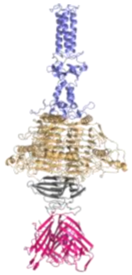			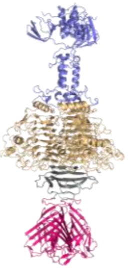	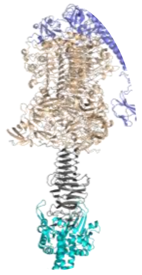	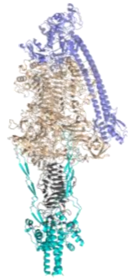	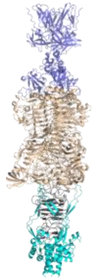	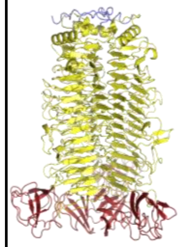	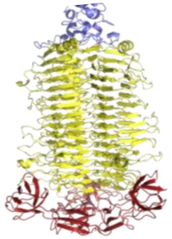

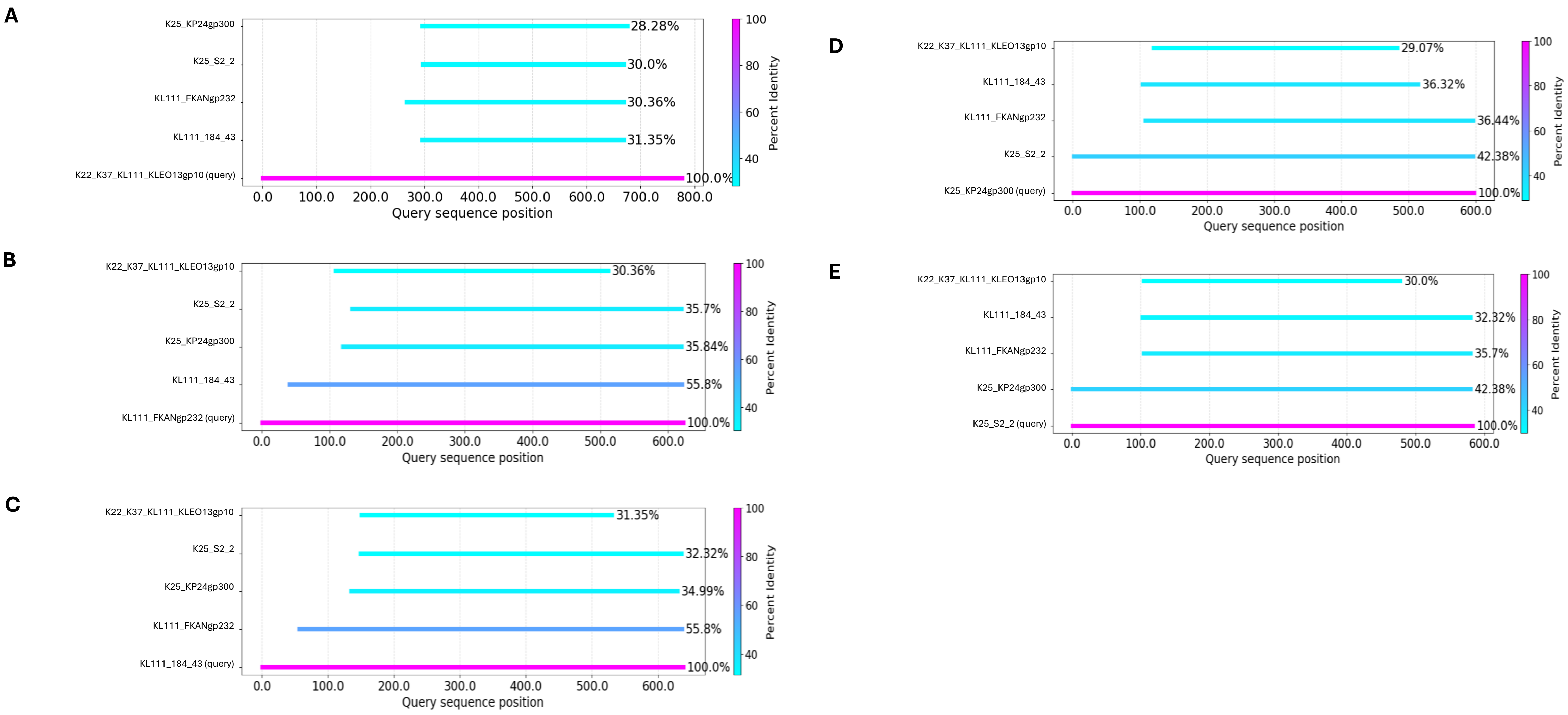

Supplementary Figure 6. Pairwise amino acid sequence comparison of five *Klebsiella pneumoniae* CPS-degrading depolymerases using BLASTP. Each panel (A-E) displays an alignment of one query protein against remaining four.

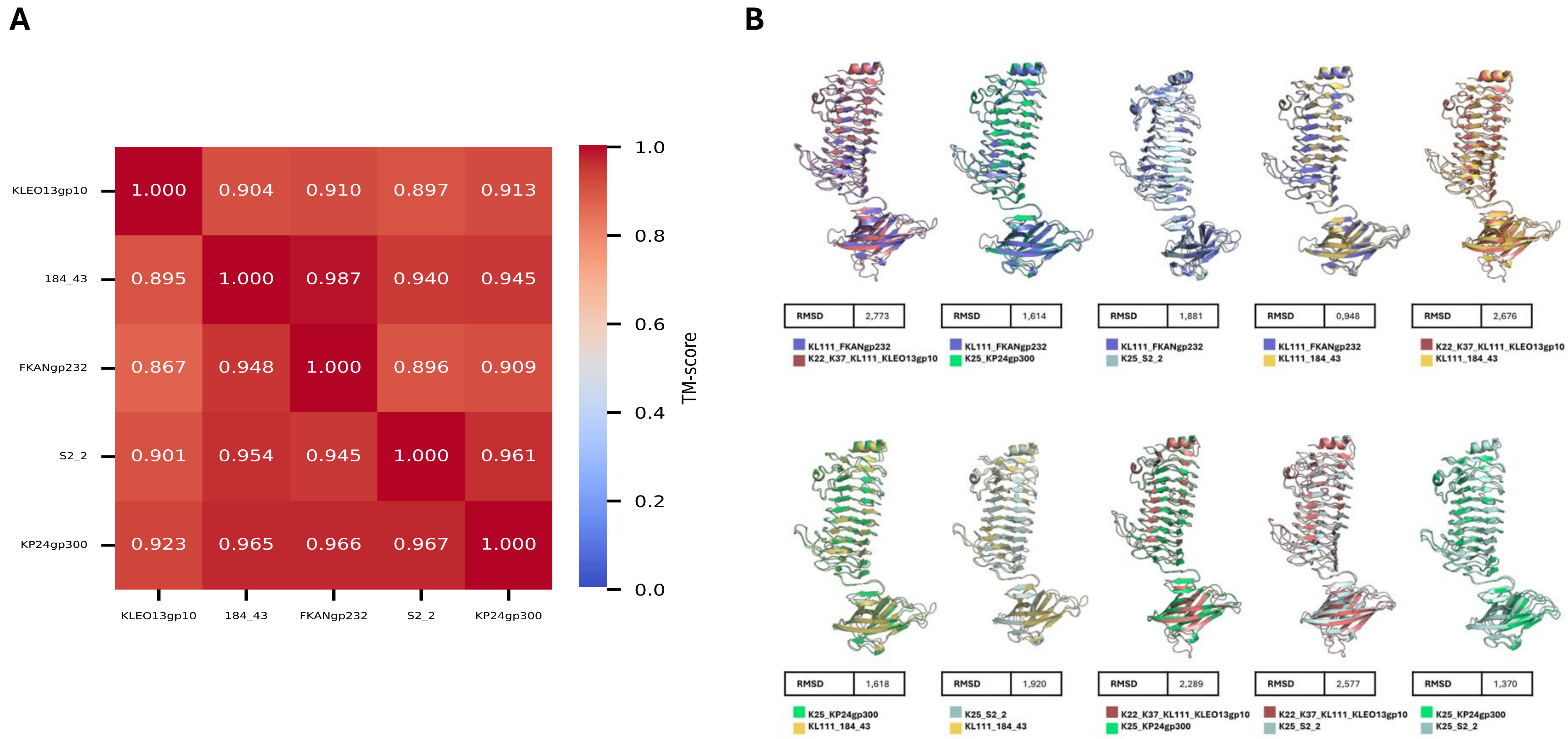

Supplementary Figure 7. Structural comparison of five *K. pneumoniae* CPS- degrading depolymerases (excluding N-terminal domains). (A) Heatmap of pairwise structural similarity between depolymerases, calculated using USalign. Color intensity corresponds to TM- scores, with higher values indicating greater structural similarity. (B) Structural alignments of protein pairs visualised in PyMOL. Each alignment includes root-mean-square-deviation (RMSD) and TM- score values to quantify structural congruence.

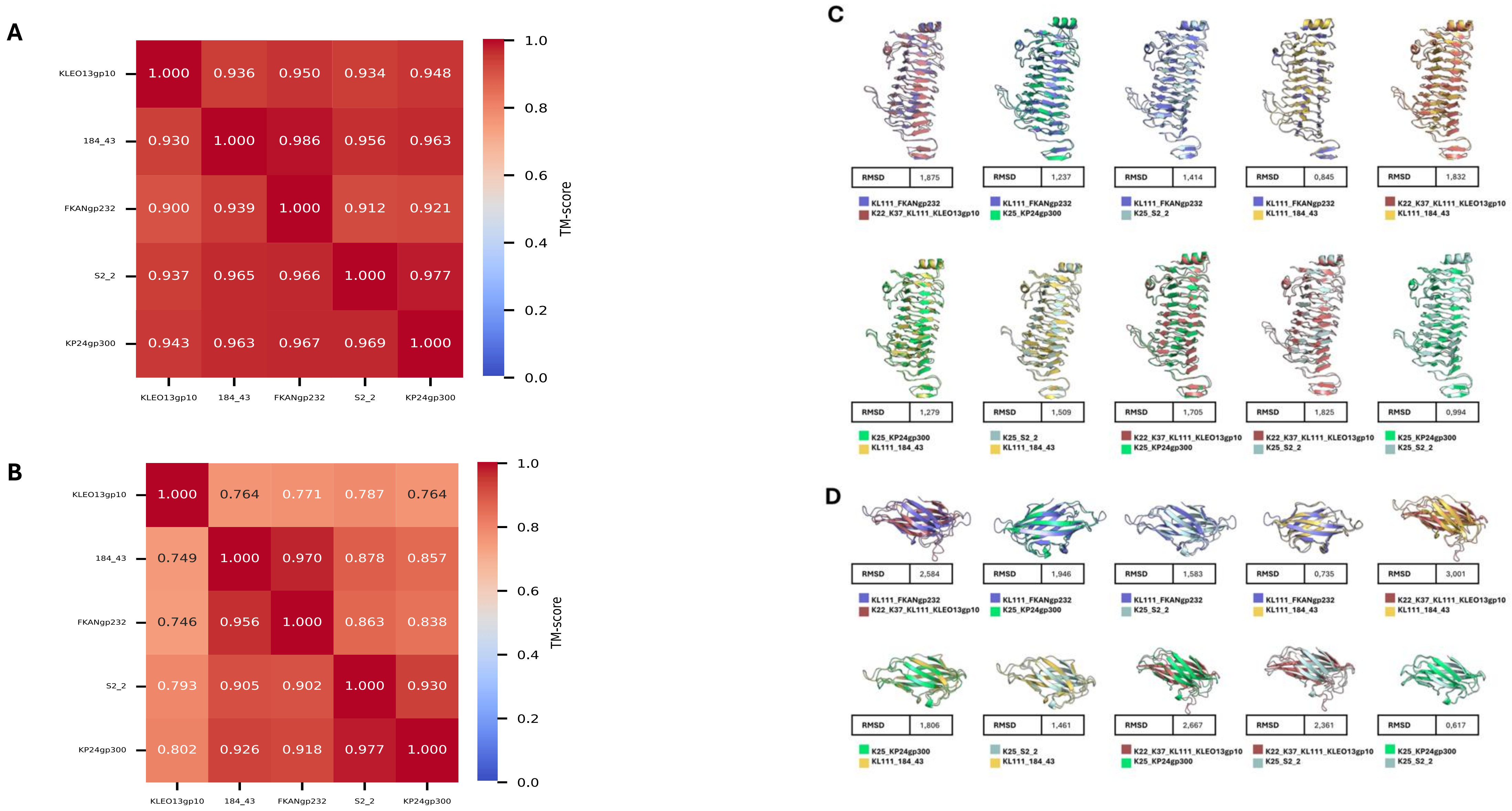

Supplementary Figure 8. Domain-specific structural comparison of *K. pneumoniae* CPS- degrading depolymerases. (A) Heatmap of pairwise structural similarity between the central domains of depolymerases, computed using USalign. TM-scores are color-coded, with higher values indicating greater structural similarity. (B) Heatmap of C-terminal domains comparisons, also based on TM-scores calculated using USalign. (C) Structural alignments of the central domains visualized in PyMOL, with associated root-mean-square deviation (RMSD) and TM-score values provided for each pairwise alignment. (D) Structural alignments of the C-terminal domains, also shown with root-mean-square deviations (RMSD) and TM-scores.

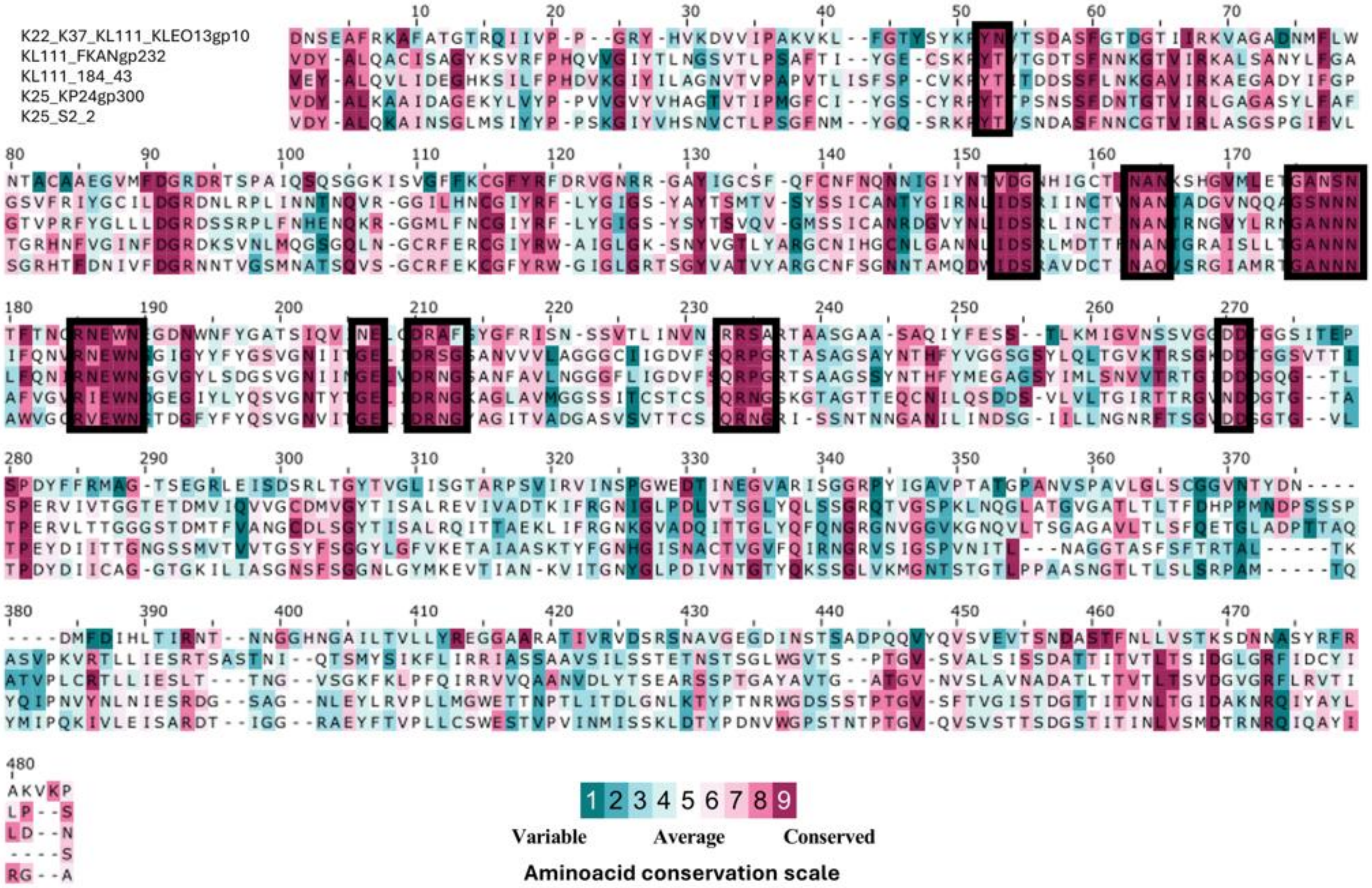

Supplementary Figure 9. Conservation-based analysis of *K. pneumoniae* CPS- degrading depolymerases. Multiple sequence alignments (MSA) of five depolymerases, with amino acid conservation colouring according to ConSurf scores. Conservation level range from variable (low scores) to highly conserved (high scores), visually emphasizing conserved motifs. Residues corresponding to the predicted active site regions marked with black rectangles, highlighting their conservation across sequences and spatial co- localization in the structures. This representation provides a sequence-level view of conserved, functionally relevant regions across the protein set.

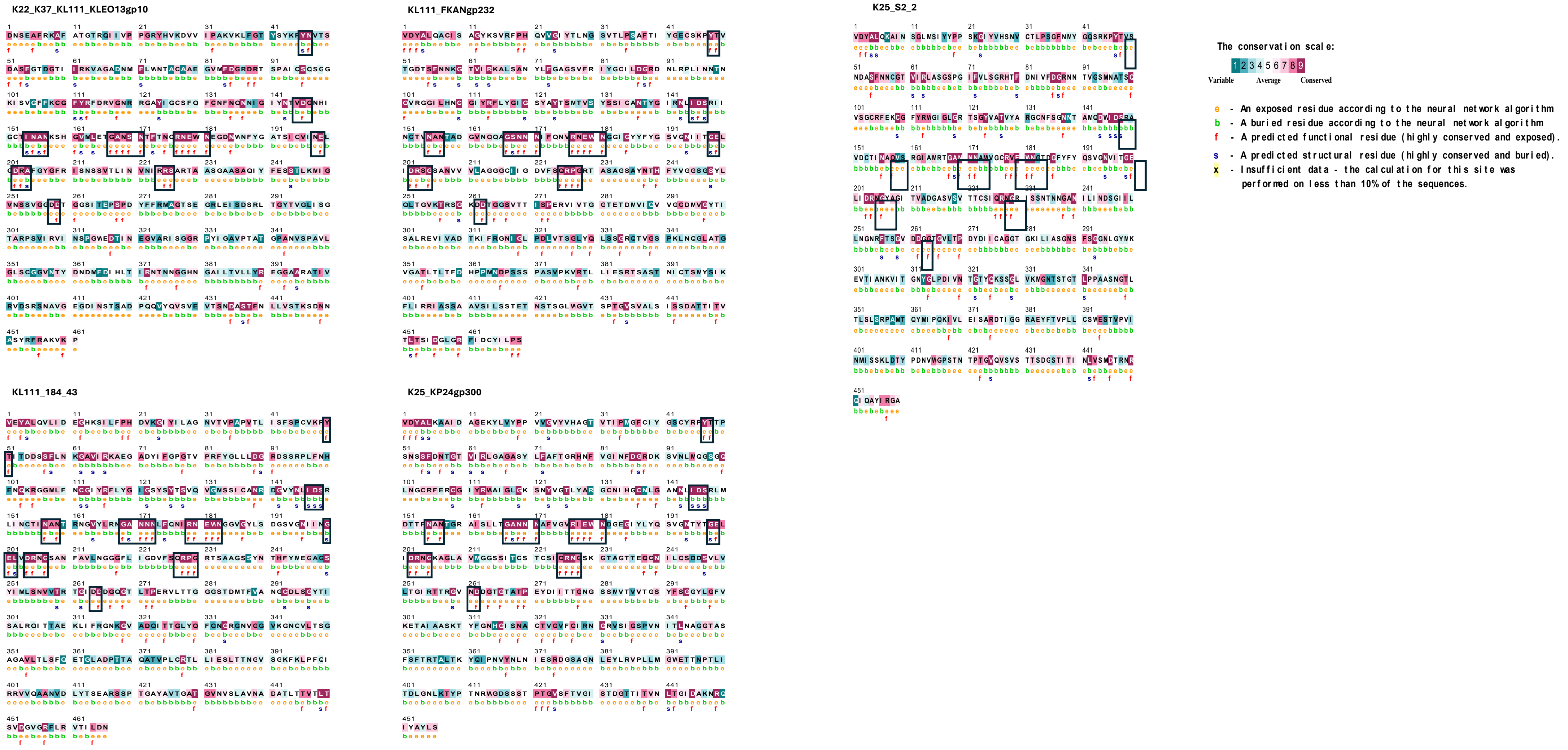

Supplementary Figure 10. Conservation-based analysis of *K. pneumoniae* CPS-degrading depolymerases. Each panel displays amino acid sequence of depolymerase, colored according to residue conservation scores from ConSurf analysis. Below each amino acid, additional annotations indicate whether the residue is exposed or buried, or is classified as functionally, or structurally important. Residues corresponding to regions predicted as possible active site in Figure 4 are highlighted by black rectangles.

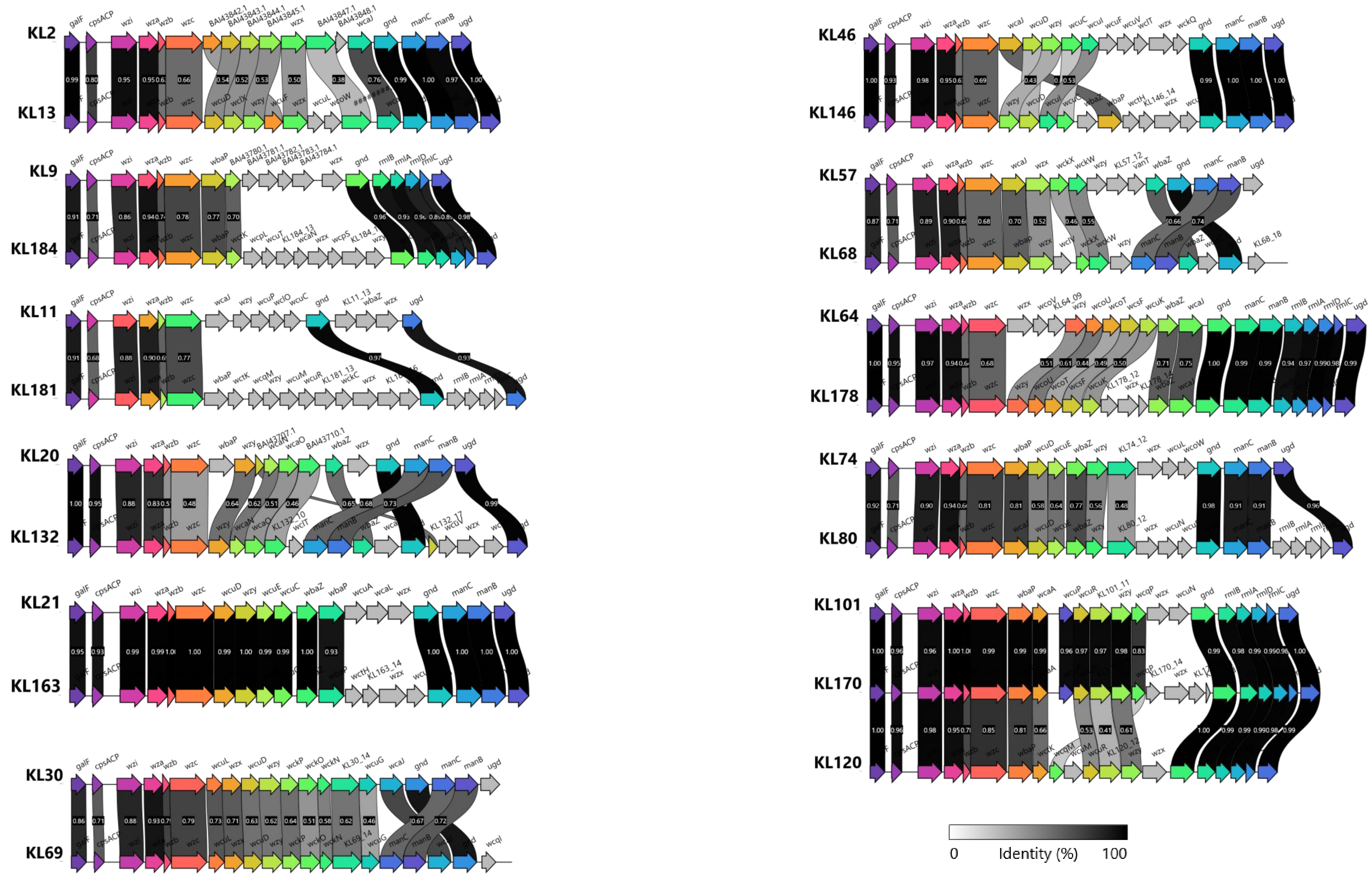

Supplementary Figure 11. Comparative genomic organization of selected CPS loci. Gene clusters are displayed with annotated sequence and cluster organisation similarities, enabling visualization of conserved and variable regions across the loci (Clinker)

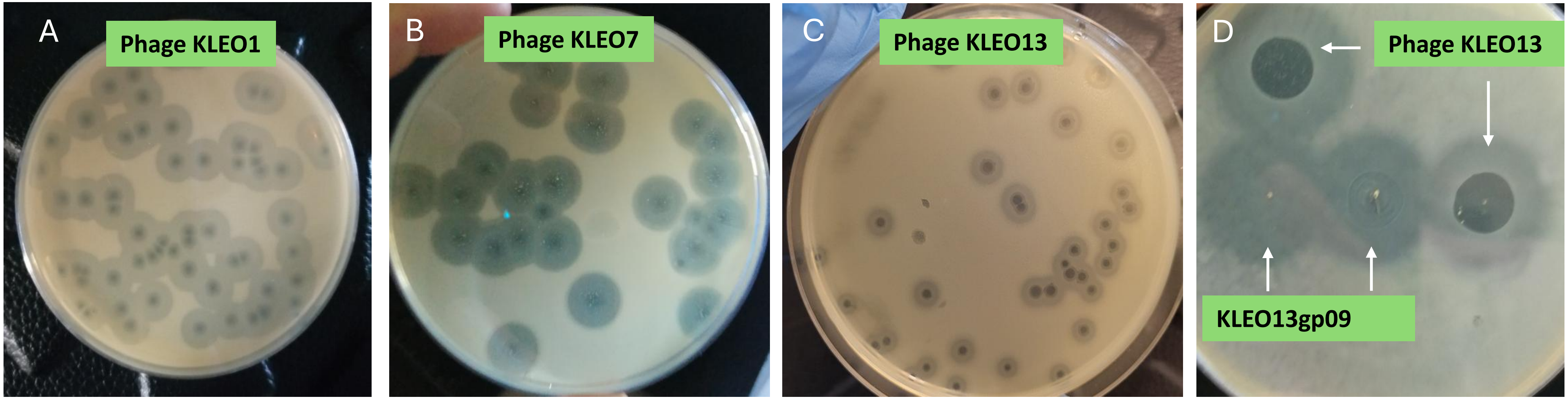

Supplementary Figure 12. Representative images illustrating phage activity with halo formation, as well as recombinant enzyme halo visualization. Phage titration with single plaques with halo zones (A-C); Spot test - phage with halo zone and halo zone of recombinant depolymerase (D).

Supplementary References:

1. Patro, L. P. P., Sudhakar, K. U. & Rathinavelan, T. K-PAM: a unified platform to distinguish Klebsiella species K- and O-antigen types, model antigen structures and identify hypervirulent strains. *Sci Rep* **10**, 16732 (2020).
2. Huang, T. *et al*. Structural and functional basis of bacteriophage K64-ORF41 depolymerase for capsular polysaccharide degradation of *Klebsiella pneumoniae* K64. *International Journal of Biological Macromolecules* **265**, 130917 (2024).
3. Tu, I.-F. *et al*. Structural and biological insights into Klebsiella pneumoniae surface polysaccharide degradation by a bacteriophage K1 lyase: implications for clinical use. *Journal of Biomedical Science* **29**, 9 (2022).
4. Dunstan, R. A. *et al*. Mechanistic Insights into the Capsule-Targeting Depolymerase from a Klebsiella pneumoniae Bacteriophage. *Microbiol Spectr* **9**, e0102321 (2021).
5. Lin, T.-L. *et al*. Development of Klebsiella pneumoniae Capsule Polysaccharide-Conjugated Vaccine Candidates Using Phage Depolymerases. *Front. Immunol.* **13**, (2022).
6. Ye, T.-J. *et al*. Klebsiella pneumoniae K2 capsular polysaccharide degradation by a bacteriophage depolymerase does not require trimer formation. *mBio* **15**, e03519 (2024).
7. Cai, R. *et al*. Structural biology and functional features of phage-derived depolymerase Depo32 on Klebsiella pneumoniae with K2 serotype capsular polysaccharides. *Microbiol Spectr* **11**, e0530422 (2023).
8. Lukianova, A. A. *et al*. Depolymerisation of the Klebsiella pneumoniae Capsular Polysaccharide K21 by Klebsiella Phage K5. *International Journal of Molecular Sciences* **24**, 17288 (2023).
9. Squeglia, F. *et al*. Structural and Functional Studies of a Klebsiella Phage Capsule Depolymerase Tailspike: Mechanistic Insights into Capsular Degradation. *Structure* **28**, 613-624.e4 (2020).
10. Noreika, A., Stankevičiūtė, J., Rutkienė, R., Meškys, R. & Kalinienė, L. Exploring the enzymatic activity of depolymerase gp531 from *Klebsiella pneumoniae* jumbo phage RaK2. *Virus Research* **336**, 199225 (2023).
11. Volozhantsev, N. *et al*. Characterization and Therapeutic Potential of Bacteriophage-Encoded Polysaccharide Depolymerases with β Galactosidase Activity against Klebsiella pneumoniae K57 Capsular Type. *Antibiotics* **9**, 732 (2020).
12. Maciejewska, B. *et al*. Klebsiella phage KP34gp57 capsular depolymerase structure and function: from a serendipitous finding to the design of active mini-enzymes against K. pneumoniae. *mBio* **14**, e0132923 (2023).
13. Kasimova, A. A. *et al*. The structure of Klebsiella pneumoniae K108 capsular polysaccharide is similar to Escherichia coli colanic acid. *Int J Biol Macromol* **244**, 125403 (2023).
