# Supplementary material for: DepoCatalog: mapping diversity of 129 recombinantly produced Klebsiella phage depolymerases
Source: Nat Commun. 2026 May 22;17:6724. doi: 10.1038/s41467-026-73570-7 (PMC13385381; doi:10.1038/s41467-026-73570-7)
Supplement: Supplementary file 2 — Description of Additional Supplementary Files [file 41467_2026_73570_MOESM2_ESM.pdf]

Description of Additional Supplementary Files:

Supplementary Data 1: Klebsiella spp. strain collection representing capsular K-types used in this study

Supplementary Data 2: Phage and depolymerase activity profiling across the Klebsiella capsular K-type strain collection evaluated in a spot test

Supplementary Data 3: Comprehensive characteristics of Klebsiella phage depolymerases analyzed in this study

Supplementary Data 4: AlphaFold3.0 confidence scores for predicted depolymerase structures

Supplementary Data 5: Expression conditions for recombinant depolymerases used in this study

Supplementary Data 6: AlphaFold3.0 models with pLDDT score overlaid from two viewing angles, and the Predicted aligned error (PAE) of analyzed depolymerases
