# Supplementary material for: DepoCatalog: mapping diversity of 129 recombinantly produced Klebsiella phage depolymerases
Source: Nat Commun. 2026 May 22;17:6724. doi: 10.1038/s41467-026-73570-7 (PMC13385381; doi:10.1038/s41467-026-73570-7)
Supplement: Supplementary file 8 — Supplementary Dataset 6 [file 41467_2026_73570_MOESM8_ESM.pdf]

Confidence score (pLDDT)

>90 | 90 - 70 | 69-50 | <50

Supplementary Data 6. AlphaFold3.0 models with pLDDT score overlaid from two viewing angles and the Predicted aligned error (PAE) of analyzed depolymerases.

S2-4	GBH001_056	K1-ORF34	gp47	KLEO1gp03	Kpv71_52	gp09
**Mean PAE:** 17,92	**Mean PAE:** 7,98	**Mean PAE:** 8,00	**Mean PAE:** 8,54	**Mean PAE:** 10,84	**Mean PAE:** 12,17	**Mean PAE:** 8,29
Front view / Side view	Front view / Side view	Front view / Side view	Front view / Side view	Front view / Side view	Front view / Side view	Front view / Side view

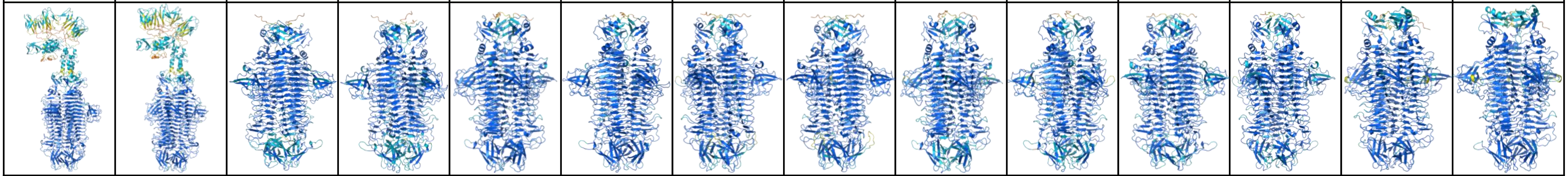

B1dep	DpK2	Depo32	BMacgp22	gp81	NPatgp22	K2-ORF16
**Mean PAE:** 15,25	**Mean PAE:** 19,85	**Mean PAE:** 15,02	**Mean PAE:** 15,14	**Mean PAE:** 15,18	**Mean PAE:** 16,39	**Mean PAE:** 12,89
Front view / Side view	Front view / Side view	Front view / Side view	Front view / Side view	Front view / Side view	Front view / Side view	Front view / Side view

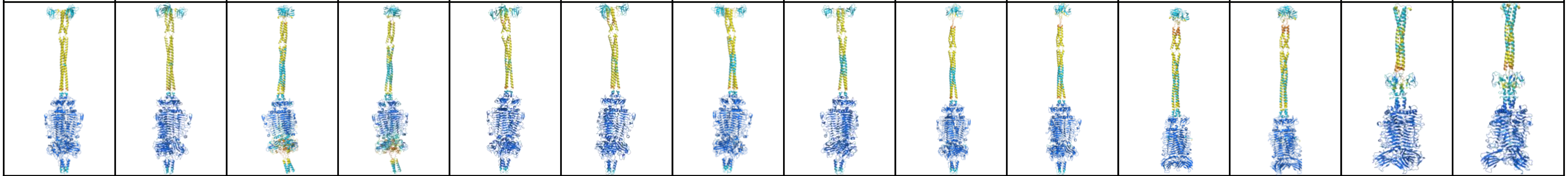

KP24gp196	Kpv74_56	Dep1979	GBH038_054	K2-2	KLEO13gp09	KP32gp37
**Mean PAE:** 7,61	**Mean PAE:** 8,04	**Mean PAE:** 7,27	**Mean PAE:** 8,04	**Mean PAE:** 7,60	**Mean PAE:** 7,00	**Mean PAE:** 20,65
Front view / Side view	Front view / Side view	Front view / Side view	Front view / Side view	Front view / Side view	Front view / Side view	Front view / Side view

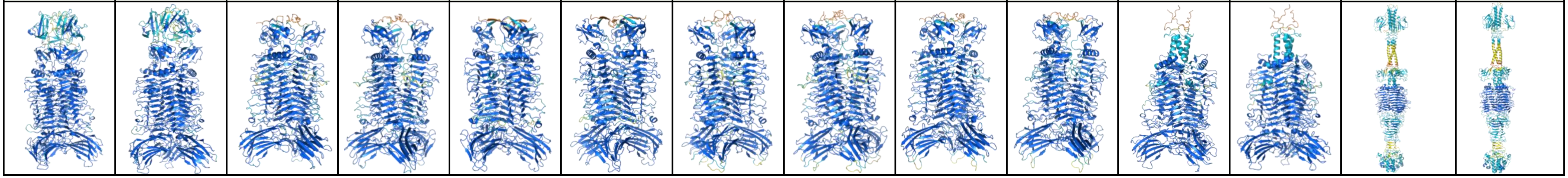

Supplementary Data 6. AlphaFold3.0 models with pLDDT score overlaid from two viewing angles and the Predicted aligned error (PAE) of analyzed depolymerases (continued).

Confidence score (pLDDT): >90 | 90 - 70 | 69-50 | <50

CDS_0187	0391_03	CDS_0182	dep1011	K5-4 ORF38	CDS_0189	K7dep
**Mean PAE:** 10,82	**Mean PAE:** 14,11	**Mean PAE:** 9,91	**Mean PAE:** 14,26	**Mean PAE:** 7,20	**Mean PAE:** 8,93	**Mean PAE:** 24,93
Front view / Side view	Front view / Side view	Front view / Side view	Front view / Side view	Front view / Side view	Front view / Side view	Front view / Side view
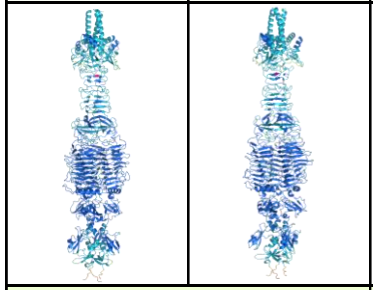	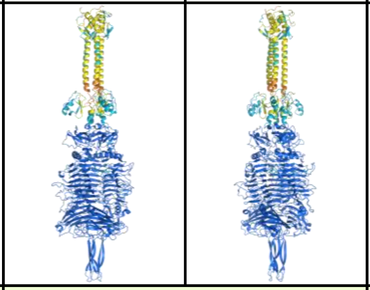	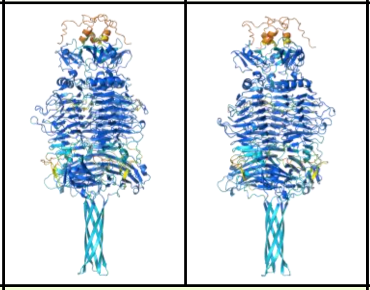	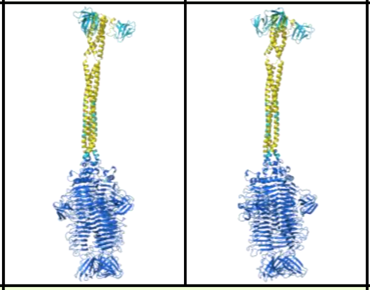	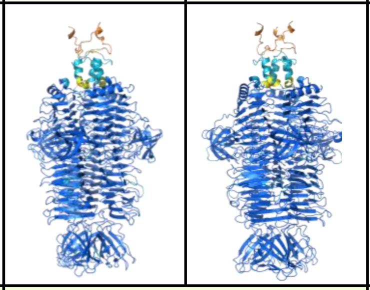	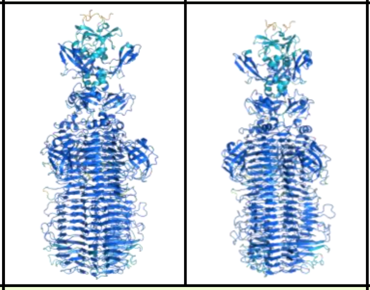	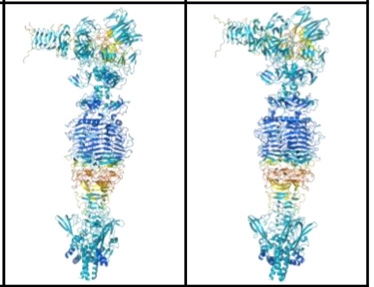

K5-4 ORF37	FKANgp229	KLEO25gp59	K11gp17	S1-1	0574_17	KLEO26gp185
**Mean PAE:** 20,09	**Mean PAE:** 11,53	**Mean PAE:** 16,14	**Mean PAE:** 18,14	**Mean PAE:** 8,80	**Mean PAE:** 10,54	**Mean PAE:** 8,46
Front view / Side view	Front view / Side view	Front view / Side view	Front view / Side view	Front view / Side view	Front view / Side view	Front view / Side view
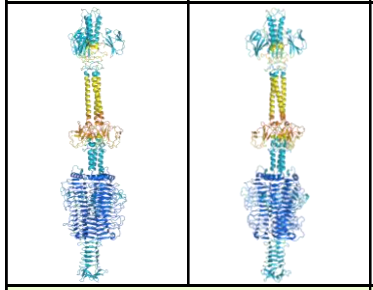	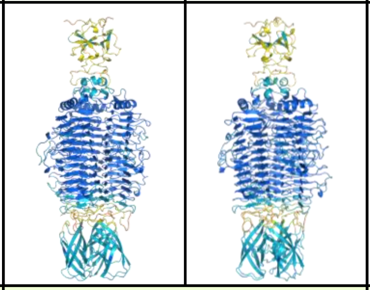	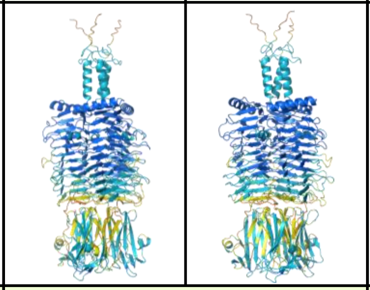	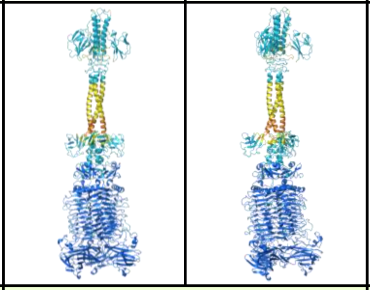	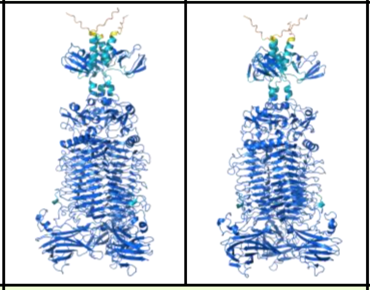	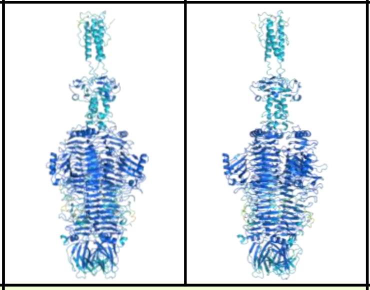	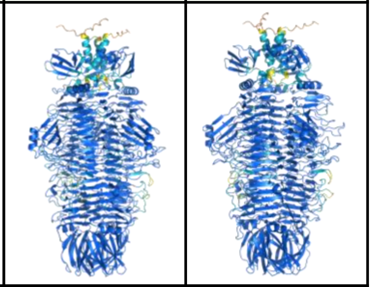

KP24gp304	Kl-dep	K20dep	S1-3	KP32gp38	RBP2	KLEO13gp10
**Mean PAE:** 13,06	**Mean PAE:** 17,43	**Mean PAE:** 11,83	**Mean PAE:** 10,08	**Mean PAE:** 9,21	**Mean PAE:** 5,64	**Mean PAE:** 19,10
Front view / Side view	Front view / Side view	Front view / Side view	Front view / Side view	Front view / Side view	Front view / Side view	Front view / Side view
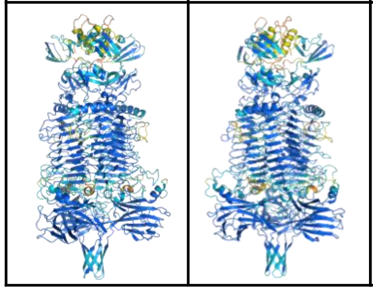	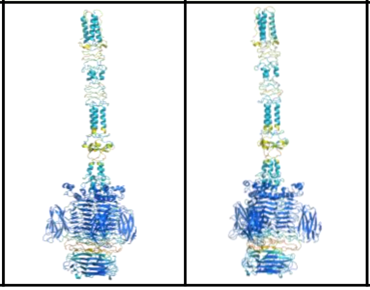	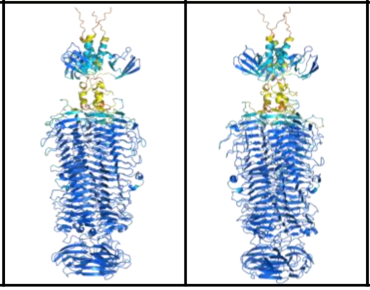	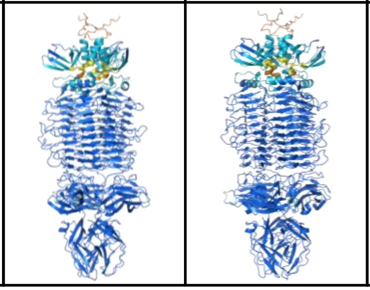	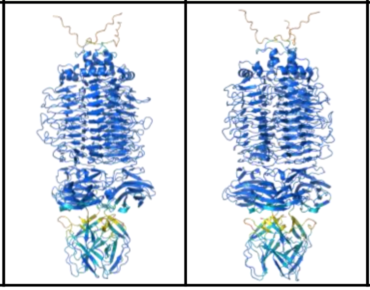	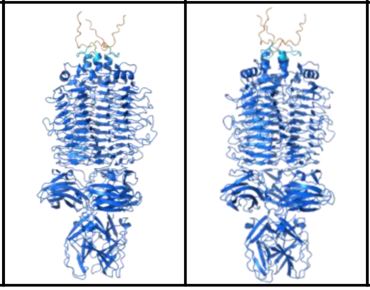	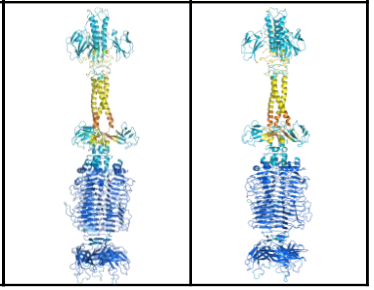

Confidence score (pLDDT): >90 | 90 - 70 | 69-50 | <50

Supplementary Data 6. AlphaFold3.0 models with pLDDT score overlaid from two viewing angles and the Predicted aligned error (PAE) of analyzed depolymerases (continued).

**1409_59**	**1441_47**	**1248_57**	**DepS8**	**Dep622**	**KP24gp300**	**S2-2**
**Mean PAE:** 18,92	**Mean PAE:** 13,09	**Mean PAE:** 13,44	**Mean PAE:** 7,25	**Mean PAE:** 5,91	**Mean PAE:** 8,07	**Mean PAE:** 7,81
Front view / Side view	Front view / Side view	Front view / Side view	Front view / Side view	Front view / Side view	Front view / Side view	Front view / Side view
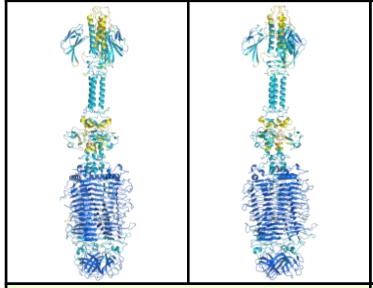	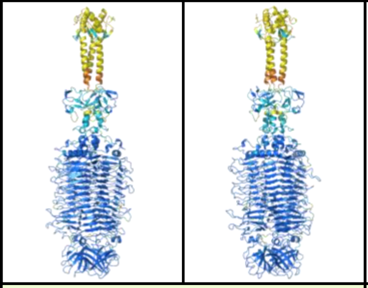	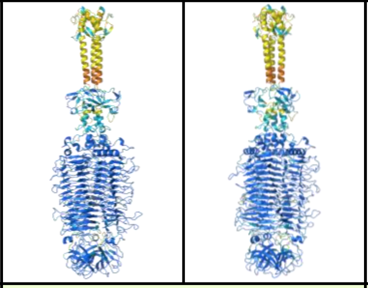	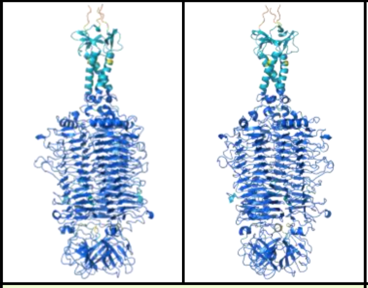	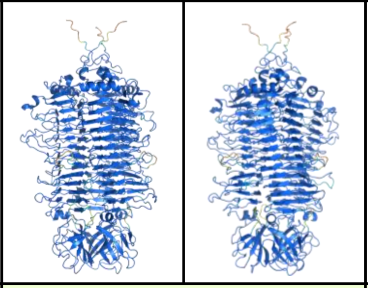	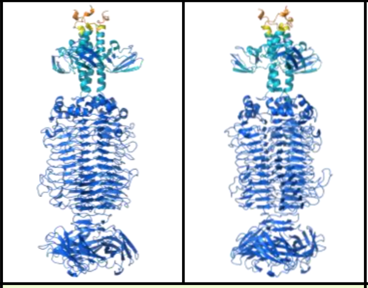	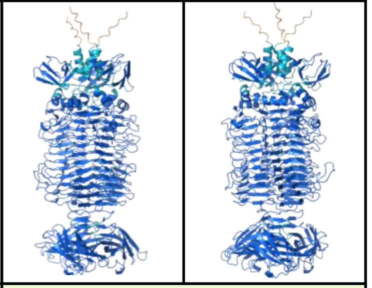
**K27dep**	**gp12**	**1251_37**	**FKANgp225**	**Dop5**	**K5-2 ORF37**	**S2-6**
**Mean PAE:** 18,84	**Mean PAE:** 17,18	**Mean PAE:** 10,47	**Mean PAE:** 8,73	**Mean PAE:** 22,36	**Mean PAE:** 20,48	**Mean PAE:** 17,53
Front view / Side view	Front view / Side view	Front view / Side view	Front view / Side view	Front view / Side view	Front view / Side view	Front view / Side view
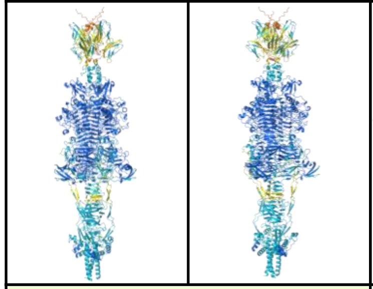	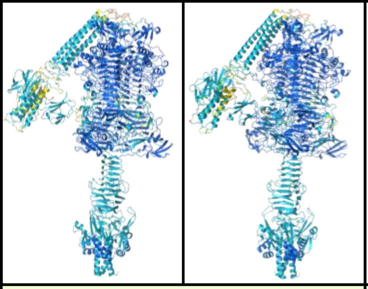	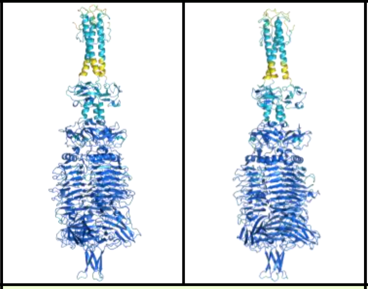	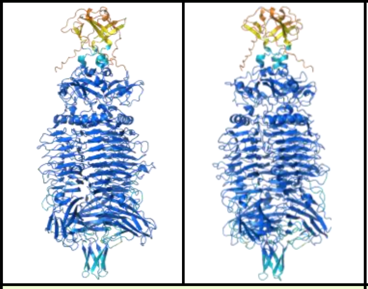	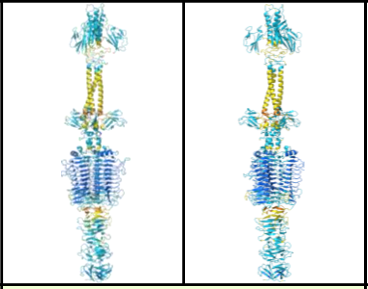	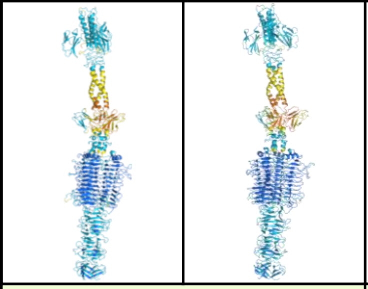	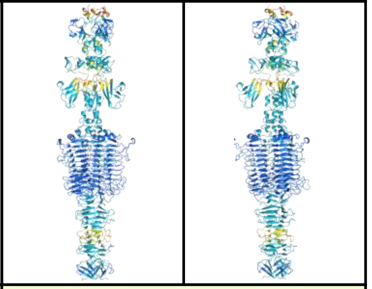
**K11gp0043**	**KLEO26gp187**	**914_77**	**KP24gp301**	**CDS_0180**	**S2-3**	**KLEO26gp181**
**Mean PAE:** 7,03	**Mean PAE:** 8,72	**Mean PAE:** 6,99	**Mean PAE:** 19,57	**Mean PAE:** 20,19	**Mean PAE:** 13,09	**Mean PAE:** 21,75
Front view / Side view	Front view / Side view	Front view / Side view	Front view / Side view	Front view / Side view	Front view / Side view	Front view / Side view
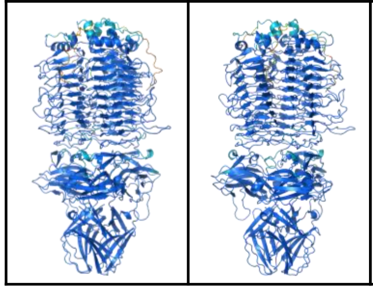	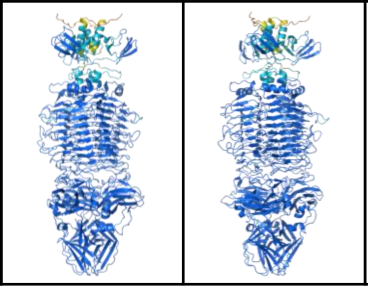	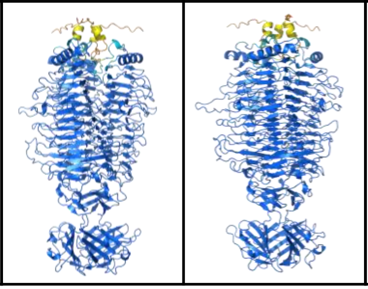	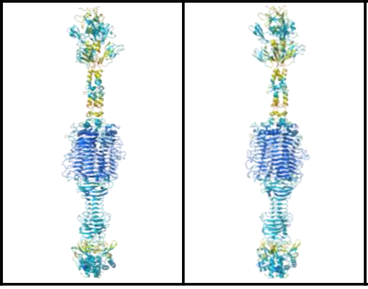	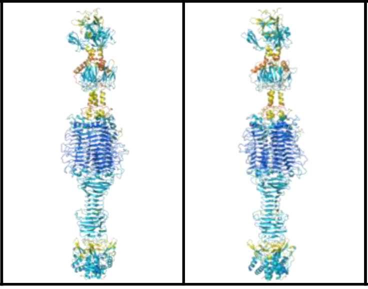	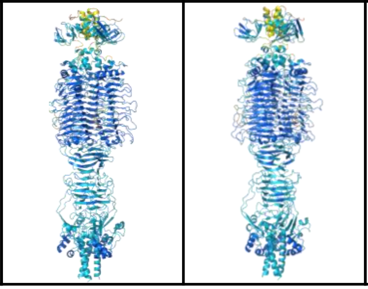	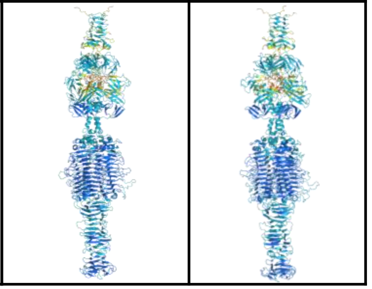

Supplementary Data 6. AlphaFold3.0 models with pLDDT score overlaid from two viewing angles and the Predicted aligned error (PAE) of analyzed depolymerases (continued).

Confidence score (pLDDT): >90 | 90 - 70 | 69-50 | <50

617_77	FKANgp227	KP24gp308	248_38	Dep42	Dpo42	KLEO27gp5
**Mean PAE:** 17,47	**Mean PAE:** 16,59	**Mean PAE:** 13,43	**Mean PAE:** 10,55	**Mean PAE:** 19,55	**Mean PAE:** 19,74	**Mean PAE:** 19,54
Front view / Side view	Front view / Side view	Front view / Side view	Front view / Side view	Front view / Side view	Front view / Side view	Front view / Side view
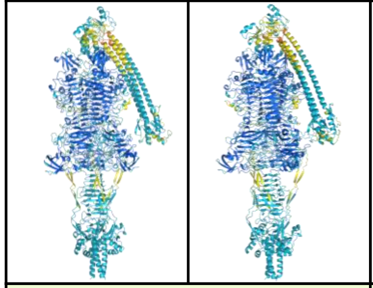	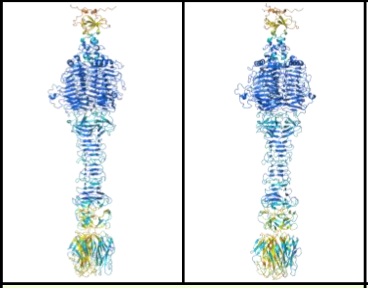	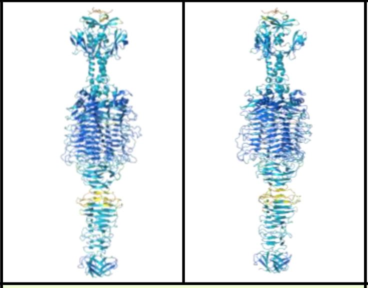	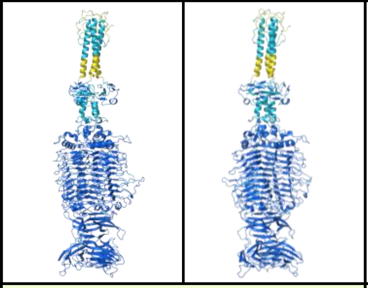	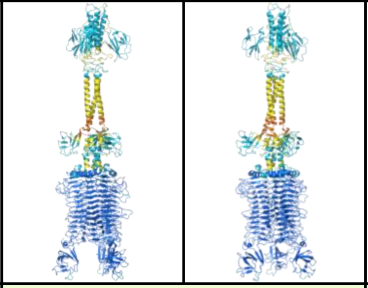	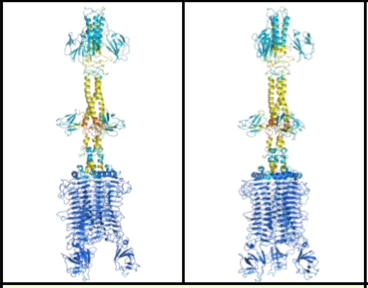	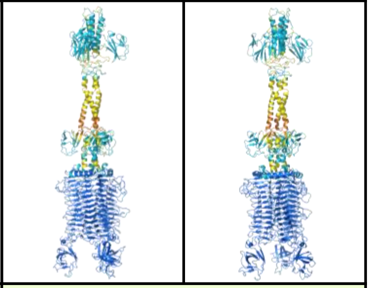

Dpo43	KLEO27gp6	GBH019_279	434_33	gp531	Dep_Z	Dep_C
**Mean PAE:** 6,89	**Mean PAE:** 6,85	**Mean PAE:** 8,95	**Mean PAE:** 21,62	**Mean PAE:** 20,90	**Mean PAE:** 18,71	**Mean PAE:** 15,51
Front view / Side view	Front view / Side view	Front view / Side view	Front view / Side view	Front view / Side view	Front view / Side view	Front view / Side view
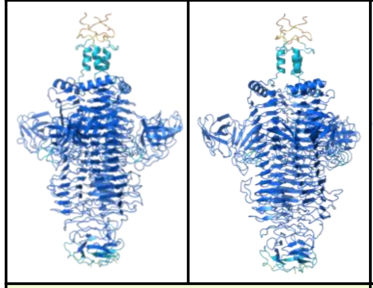	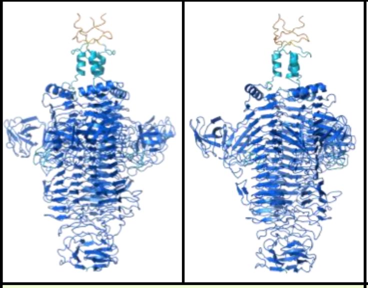	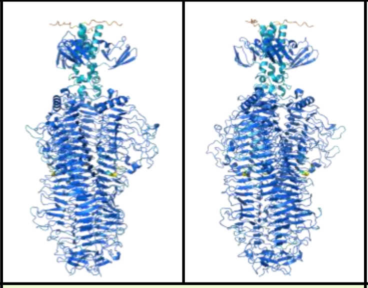	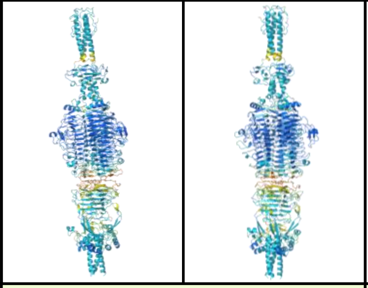	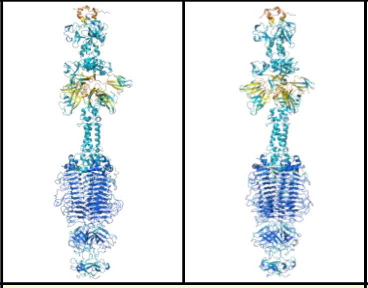	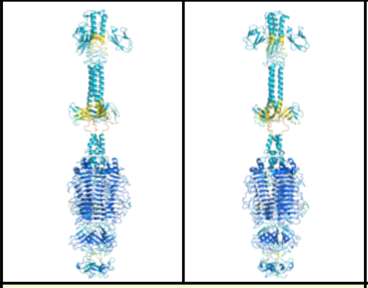	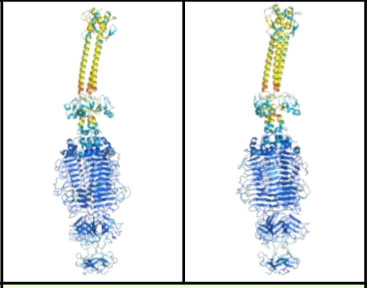

Dep_Y	CDS_0179	K56dep	Dep_kpv767	CDS_0060	Dep_kpv79	gp157
**Mean PAE:** 14,28	**Mean PAE:** 13,04	**Mean PAE:** 7,99	**Mean PAE:** 19,46	**Mean PAE:** 7,26	**Mean PAE:** 13,41	**Mean PAE:** 7,59
Front view / Side view	Front view / Side view	Front view / Side view	Front view / Side view	Front view / Side view	Front view / Side view	Front view / Side view
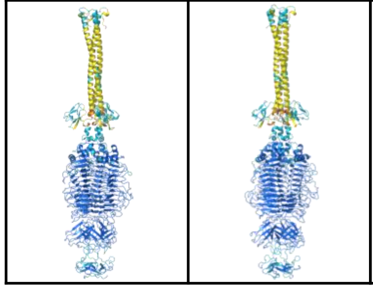	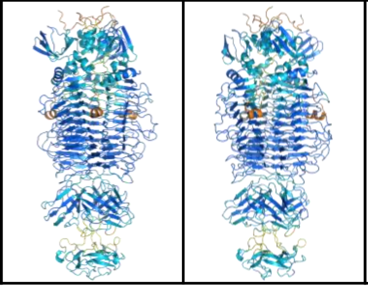	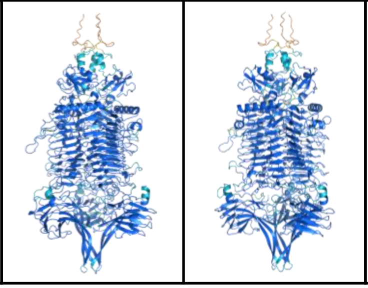	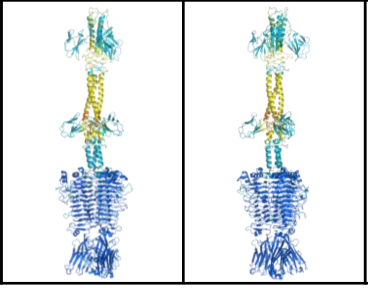	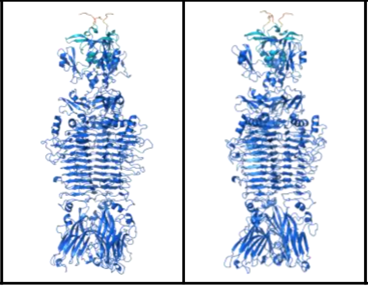	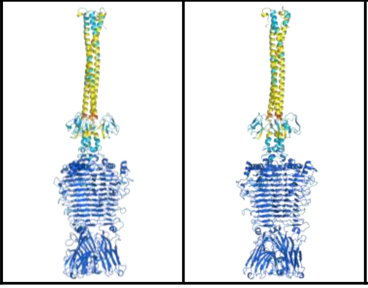	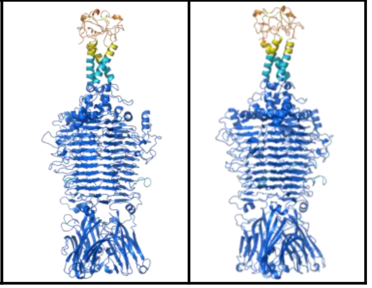

Confidence score (pLDDT): >90 | 90 - 70 | 69-50 | <50

Supplementary Data 6. AlphaFold3.0 models with pLDDT score overlaid from two viewing angles and the Predicted aligned error (PAE) of analyzed depolymerases (continued).

Dep_ZX1		KLEO7gp25		1723_59		1724_71		KP24gp310		0367_12		0391_11	
**Mean PAE:** 7,24		**Mean PAE:** 8,45		**Mean PAE:** 18,29		**Mean PAE:** 14,77		**Mean PAE:** 9,12		**Mean PAE:** 18,65		**Mean PAE:** 18,28	
Front view	Side view	Front view	Side view	Front view	Side view	Front view	Side view	Front view	Side view	Front view	Side view	Front view	Side view
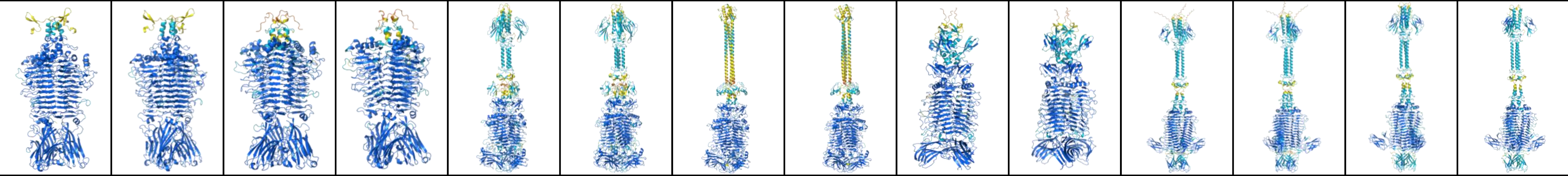													
**K62-Dpo30**		**914_74**		**KP36gp50**		**KP34gp57**		**P510dep**		**K64-ORF41**		**KLEO28gp32**	
**Mean PAE:** 15,62		**Mean PAE:** 13,26		**Mean PAE:** 16,57		**Mean PAE:** 6,15		**Mean PAE:** 13,94		**Mean PAE:** 13,49		**Mean PAE:** 13,79	
Front view	Side view	Front view	Side view	Front view	Side view	Front view	Side view	Front view	Side view	Front view	Side view	Front view	Side view
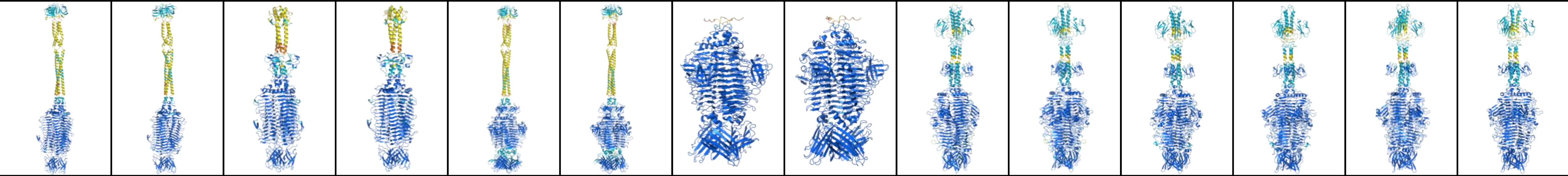													
**S2-5**		**1091_44**		**CDS_0184**		**KP24gp303**		**CDS_0190_1**		**KN1dep**		**Dp42**	
**Mean PAE:** 14,72		**Mean PAE:** 16,86		**Mean PAE:** 9,85		**Mean PAE:** 7,07		**Mean PAE:** 9,03		**Mean PAE:** 19,71		**Mean PAE:** 20,08	
Front view	Side view	Front view	Side view	Front view	Side view	Front view	Side view	Front view	Side view	Front view	Side view	Front view	Side view
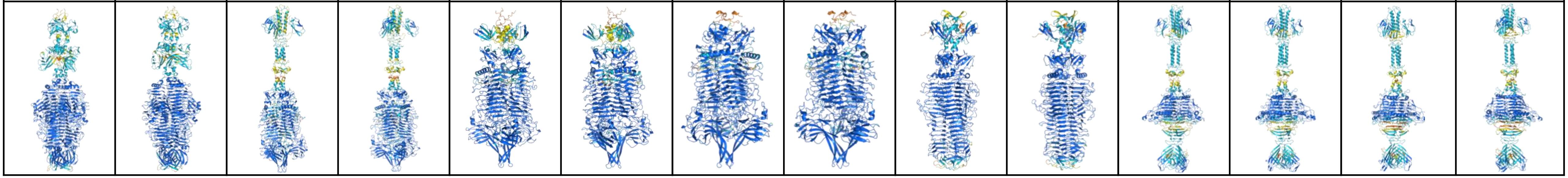													

Confidence score (pLDDT): >90 | 90 - 70 | 69-50 | <50

Supplementary Data 6. AlphaFold3.0 models with pLDDT score overlaid from two viewing angles and the Predicted aligned error (PAE) of analyzed depolymerases (continued).

**ORF96**	**Dep108.2**	**Dep108.1**	**FKANgp223**	**184_43**	**FKANgp232**	**KP24gp307**
**Mean PAE:** 20,67	**Mean PAE:** 7,20	**Mean PAE:** 6,53	**Mean PAE:** 8,96	**Mean PAE:** 8,25	**Mean PAE:** 8,47	**Mean PAE:** 10,46
Front view / Side view	Front view / Side view	Front view / Side view	Front view / Side view	Front view / Side view	Front view / Side view	Front view / Side view
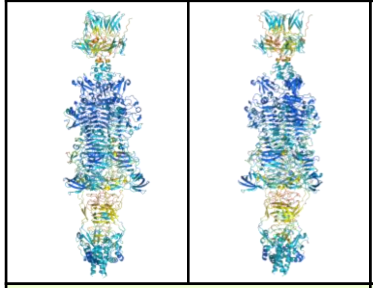	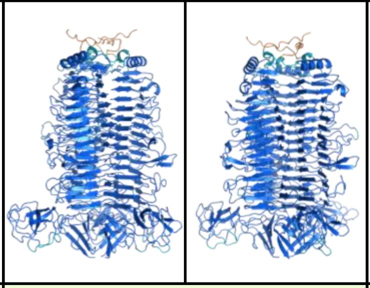	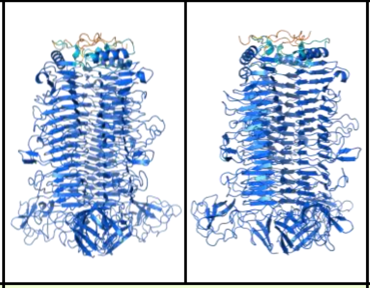	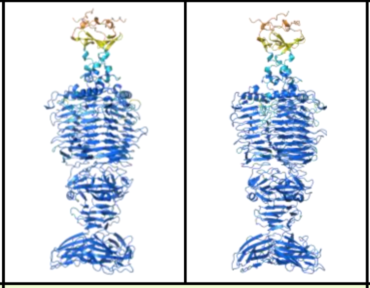	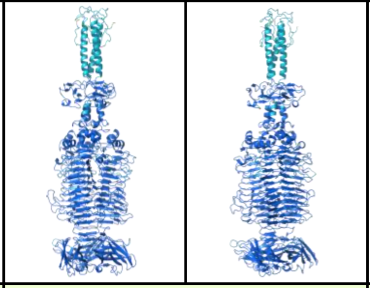	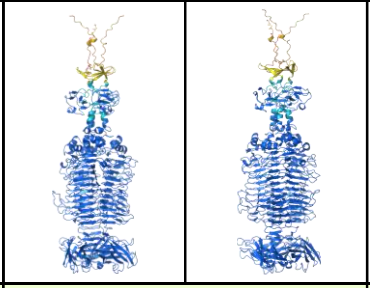	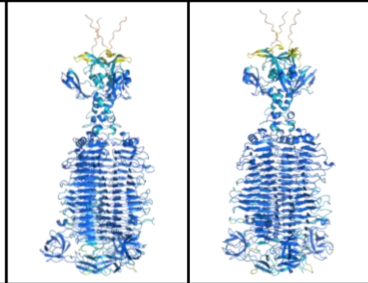
**S2-1**	**CDS_0186**	**0496_72**	**KN4dep**	**CDS_0178**	**S1-2**	**319_37**
**Mean PAE:** 25,61	**Mean PAE:** 9,89	**Mean PAE:** 19,31	**Mean PAE:** 16,50	**Mean PAE:** 17,63	**Mean PAE:** 8,92	**Mean PAE:** 8,11
Front view / Side view	Front view / Side view	Front view / Side view	Front view / Side view	Front view / Side view	Front view / Side view	Front view / Side view
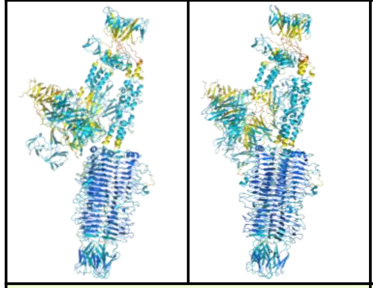	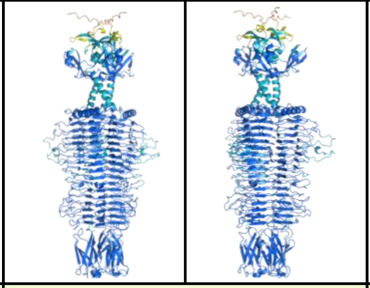	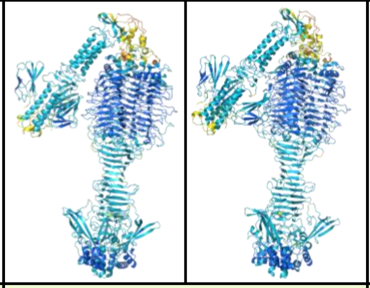	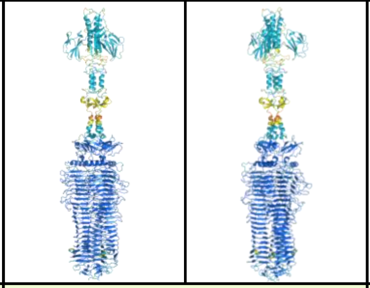	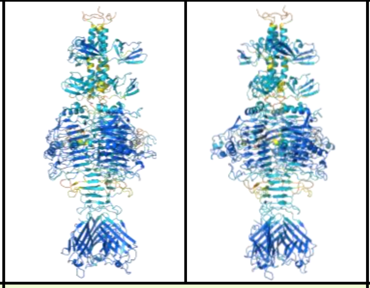	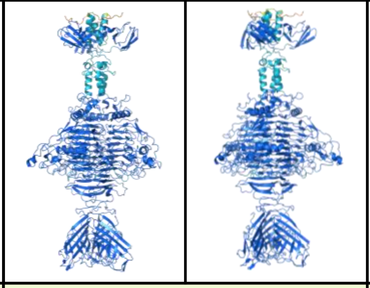	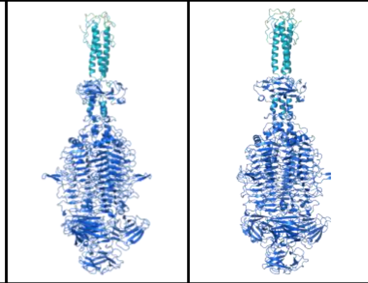
**KP24gp168**	**KP24gp306**	**738_68**	**FKANgp220**	**CDS_0191**	**CDS_0185**	**FKANgp217**
**Mean PAE:** 8,42	**Mean PAE:** 19,48	**Mean PAE:** 13,33	**Mean PAE:** 9,82	**Mean PAE:** 8,96	**Mean PAE:** 23,18	**Mean PAE:** 8,78
Front view / Side view	Front view / Side view	Front view / Side view	Front view / Side view	Front view / Side view	Front view / Side view	Front view / Side view
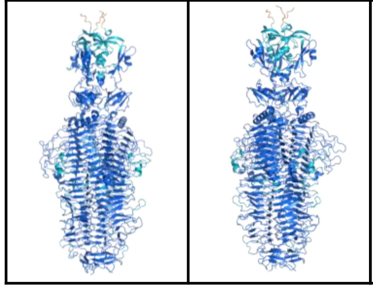	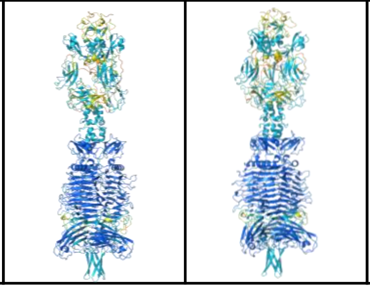	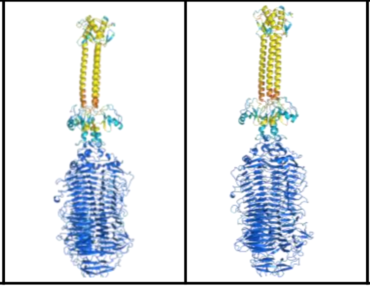	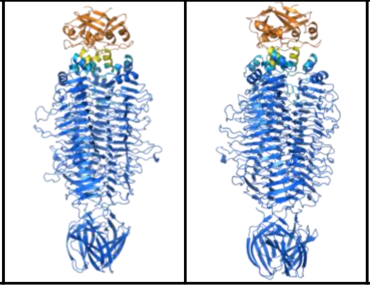	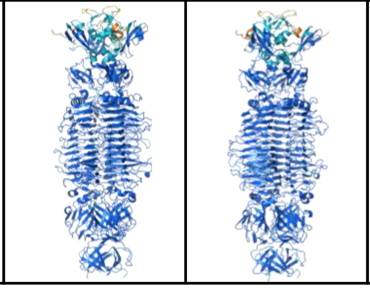	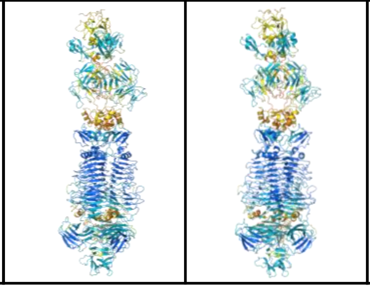	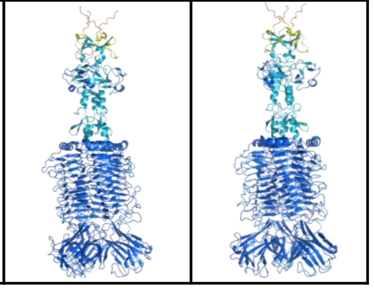

Confidence score (pLDDT)

>90 | 90 - 70 | 69-50 | <50

Supplementary Data 6. AlphaFold3.0 models with pLDDT score overlaid from two viewing angles and the Predicted aligned error (PAE) of analyzed depolymerases (continued).

**KP24gp309**		**P560dep**		**KN3dep**	
**Mean PAE:** 8,09		**Mean PAE:** 19,56		**Mean PAE:** 18,85	
Front view	Side view	Front view	Side view	Front view	Side view
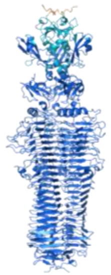	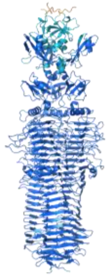	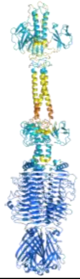	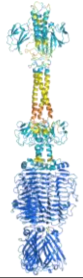	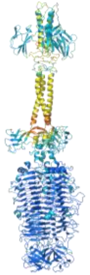	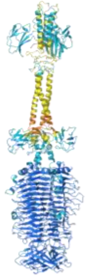
