# Supplementary material for: DepoCatalog: mapping diversity of 129 recombinantly produced Klebsiella phage depolymerases
Source: Nat Commun. 2026 May 22;17:6724. doi: 10.1038/s41467-026-73570-7 (PMC13385381; doi:10.1038/s41467-026-73570-7)
Supplement: Supplementary file 9 — Reporting Summary [file 41467_2026_73570_MOESM9_ESM.pdf]

nature portfolio

Corresponding author(s): Zuzanna Drulis-Kawa
Last updated by author(s): May 1, 2026

Reporting Summary

Nature Portfolio wishes to improve the reproducibility of the work that we publish. This form provides structure for consistency and transparency in reporting. For further information on Nature Portfolio policies, see our Editorial Policies and the Editorial Policy Checklist.

Statistics

For all statistical analyses, confirm that the following items are present in the figure legend, table legend, main text, or Methods section.

n/a	Confirmed	
☐	☒	The exact sample size (*n*) for each experimental group/condition, given as a discrete number and unit of measurement
☒	☐	A statement on whether measurements were taken from distinct samples or whether the same sample was measured repeatedly
☒	☐	The statistical test(s) used AND whether they are one- or two-sided<br>*Only common tests should be described solely by name; describe more complex techniques in the Methods section.*
☒	☐	A description of all covariates tested
☒	☐	A description of any assumptions or corrections, such as tests of normality and adjustment for multiple comparisons
☐	☒	A full description of the statistical parameters including central tendency (e.g. means) or other basic estimates (e.g. regression coefficient) AND variation (e.g. standard deviation) or associated estimates of uncertainty (e.g. confidence intervals)
☒	☐	For null hypothesis testing, the test statistic (e.g. *F*, *t*, *r*) with confidence intervals, effect sizes, degrees of freedom and *P* value noted<br>*Give P values as exact values whenever suitable.*
☒	☐	For Bayesian analysis, information on the choice of priors and Markov chain Monte Carlo settings
☒	☐	For hierarchical and complex designs, identification of the appropriate level for tests and full reporting of outcomes
☒	☐	Estimates of effect sizes (e.g. Cohen's *d*, Pearson's *r*), indicating how they were calculated

Our web collection on statistics for biologists contains articles on many of the points above.

Software and code

Policy information about availability of computer code

Data collection	Sequence homologs and conserved domains were identified using BLAST (v2.16.1+), HMMER v3.4 and HHpred. Prophage regions were detected using VirSorter2, PhySpy and BlastN with COG_KOG, PDB, PFAM, PHROGS, and UNICLUST databases. Prophage morphotypes were assigned using VIRFAM.Sequence homologs and conserved domains were identified using BLAST (v2.16.1+), HMMER v3.4 and HHpred Version: 57c8707149031cc9f8edceba362c71a3762bdbf8. Prophage regions were detected using VirSorter2 v.1.0.1, PhySpy v5.0.10, and BlastN (v2.16.1+), with COG_KOG (2025), PDB (2025), PFAM v37, PHROGs v4, and UNICLUST (2025) databases. Prophage morphotypes were assigned using VIRFAM (2025).
Data analysis	Protein structures were modelled using AlphaFold 3.0. Structural comparisons were performed using USalign (v2024.07.30) and visualised in PyMOL (v3.0). Batch processing of multiple structural alignments was automated using custom Python script. Additional structural analyses were conducted using APBS plugin (v3.4.1), ConSurf (v2025), DALI v5 (2025) and Phyre2.2 servers. Gene cluster comparisons were carried out using Clinker (v0.0.31). Structural confidence scores were extracted from AlphaFold3.0 output files (JSON and PDB formats) using custom Python v3.14 scripts.

For manuscripts utilizing custom algorithms or software that are central to the research but not yet described in published literature, software must be made available to editors and reviewers. We strongly encourage code deposition in a community repository (e.g. GitHub). See the Nature Portfolio guidelines for submitting code & software for further information.

Data

Policy information about availability of data

All manuscripts must include a data availability statement. This statement should provide the following information, where applicable:

- Accession codes, unique identifiers, or web links for publicly available datasets
- A description of any restrictions on data availability
- For clinical datasets or third party data, please ensure that the statement adheres to our policy

DATA AVAILABILITY STATEMENT
The data generated in this study are presented in the Supplementary Information and Supplementary Data 1-6. The GenBank accession numbers are listed in Supplementary Data 3 and in the Methods section. The depolymerase structure models in PDB formats generated using AlphaFold3.0 and depolymerase characteristic metadata are available at DepoCat (https://depocat.uwr.edu.pl/). Source data associated with this paper are available at https://doi.org/10.5281/zenodo.19927711

CODE AVAILABILITY STATEMENT
All custom codes have been deposited at Zenodo and are publicly available at https://doi.org/10.5281/zenodo.19927711. Any additional information required to analyze the data reported in this publication is available from the lead contact upon request.

Research involving human participants, their data, or biological material

Policy information about studies with human participants or human data. See also policy information about sex, gender (identity/presentation), and sexual orientation and race, ethnicity and racism.

Reporting on sex and gender	n/a
Reporting on race, ethnicity, or other socially relevant groupings	n/a
Population characteristics	n/a
Recruitment	n/a
Ethics oversight	n/a

Note that full information on the approval of the study protocol must also be provided in the manuscript.

Field-specific reporting

Please select the one below that is the best fit for your research. If you are not sure, read the appropriate sections before making your selection.

☒ Life sciences ☐ Behavioural & social sciences ☐ Ecological, evolutionary & environmental sciences

For a reference copy of the document with all sections, see nature.com/documents/nr-reporting-summary-flat.pdf

Life sciences study design

All studies must disclose on these points even when the disclosure is negative.

Sample size	129 phage proteins, 173 Klebsiella spp. strains belonging to K1-K82 defined serotypes or genetically classified as KL101-KL186 with some exceptions (KL106, KL129, KL147, KL150, KL152, KL154, KL156, KL157, KL159, KL160, KL162, KL171, KL172, KL175, KL176, KL179, KL180, KL182, KL185) represent most of currently known KL-types. Sample sizes were not determined by formal statistical power calculations. Instead, they were defined by the scope and availability of a curated depolymerase and host strain collection assembled to capture broad biological diversity. The study included 129 phage-borne depolymerases, comprising 72 enzymes derived from virulent phages and prophages in our collection and 57 recombinantly prepared enzymes reported in the literature, spanning podoviruses (46 proteins), siphoviruses (10 proteins), myoviruses (13 proteins), jumbo myoviruses (40 proteins), and 20 prophage-encoded proteins. This curated set was not intended to represent an exhaustive survey of all publicly available Klebsiella phage genomes, but rather a representative sampling of depolymerase diversity sufficient to establish and evaluate a classification framework. Phage and depolymerase activities were tested against a broad panel of Klebsiella spp. strains covering most currently known capsular types (K1–K82 and KL101–KL186, with specified exceptions), ensuring adequate coverage of host diversity. Consistent activity patterns observed across biological replicates indicate that the chosen sample sizes were sufficient to support the study's conclusions.
Data exclusions	Negative controls (elution buffer) and positive controls (depolymerase activity against a defined capsular serotype, when available) were included in all assays to validate experimental performance. Potential cross-contamination between depolymerase samples when screened simultaneously on a single agar plate was monitored; in cases where cross - contamination was suspected, the affected assays were excluded and independently repeated.

Replication	The depolymerase and phage specificity testing for our collection was conducted at a minimum of biological triplicate. 18 unique literature-reported depolymerases were screened at least twice on the whole KL-type collection, and the positive results were repeated in triplicate. Note: our assays qualitatively assessed depolymerase activity (presence vs. absence), rather than enabling strict quantitative comparisons. All attempts for enzyme activity were successful. There were some differences in phage-host range versus enzyme specificity/activity. In some cases, the enzyme range was broader than the phage, which suggests no propagation of phage on a particular host (no plaque ± halo), but the degrading activity of the enzyme on CPS of this host (halo formation). Sample sizes for phage activity and depolymerase specificity assays were not determined by formal statistical power calculations. Instead, experiments were performed using a minimum of three independent biological replicates, which is standard practice in microbiological and enzymatic activity assays. This level of replication is sufficient to account for biological variability, ensure reproducibility, and allow reliable identification of consistent activity patterns. Across experiments, biological triplicates produced highly consistent qualitative results, confirming that the chosen sample size was adequate for the purposes of this study.
Randomization	Randomization was not necessary for this type of study, and potential sources of bias were controlled through alternative measures, including standardized experimental conditions, the use of positive and negative controls, biological replicates, and testing against the full KL-type collection.<br>Phage and depolymerases were tested independently against the full KL-type collection. Enzymes and phages were assayed in variably composed experimental groups, which differed between experiments and biological replicates, reflecting the large-scale and iterative nature of the screening. Allocation into assays was performed per enzyme or phage without fixed grouping, and results were recorded prior to any cross-comparison. Only after data acquisition were phage and depolymerase activity profiles compared. In selected experiments, phages and their corresponding depolymerases were tested simultaneously on the same agar plate to directly compare phage host range with enzyme activity.
Blinding	Blinding was partially implemented. Phage and depolymerase activity assays were performed by different investigators on distinct subsets of enzymes and phages. Enzyme samples were labeled using sequential numeric identifiers, without indication of capsular type (KL specificity), ensuring blinding at the stage of data collection. Full blinding during data analysis was not feasible, as interpretation required matching experimental outcomes with known phage or depolymerase specificity. Blinding was therefore not fully applicable to all stages of the study. The results were marked on Petri dishes during the reading.

Reporting for specific materials, systems and methods

We require information from authors about some types of materials, experimental systems and methods used in many studies. Here, indicate whether each material, system or method listed is relevant to your study. If you are not sure if a list item applies to your research, read the appropriate section before selecting a response.

Materials & experimental systems

n/a	Involved in the study
☒	☐ Antibodies
☒	☐ Eukaryotic cell lines
☒	☐ Palaeontology and archaeology
☐	☒ Animals and other organisms
☒	☐ Clinical data
☒	☐ Dual use research of concern
☒	☐ Plants

Methods

n/a	Involved in the study
☒	☐ ChIP-seq
☒	☐ Flow cytometry
☒	☐ MRI-based neuroimaging

Animals and other research organisms

Policy information about studies involving animals; ARRIVE guidelines recommended for reporting animal research, and Sex and Gender in Research

Laboratory animals	n/a
Wild animals	n/a
Reporting on sex	n/a
Field-collected samples	n/a
Ethics oversight	n/a

Note that full information on the approval of the study protocol must also be provided in the manuscript.

Plants

Seed stocks	n/a
Novel plant genotypes	n/a
Authentication	n/a
