# Supplementary material for: DepoCatalog: mapping diversity of 129 recombinantly produced Klebsiella phage depolymerases
Source: Nat Commun. 2026 May 22;17:6724. doi: 10.1038/s41467-026-73570-7 (PMC13385381; doi:10.1038/s41467-026-73570-7)
Supplement: Supplementary file 10 — Transparent Peer Review file [file 41467_2026_73570_MOESM10_ESM.pdf]

nature portfolio

Peer Review File

DepoCatalog: mapping diversity of 129 recombinantly produced *Klebsiella* phage depolymerases

Corresponding Author: Professor Zuzanna Drulis-Kawa

This file contains all reviewer reports in order by version, followed by all author rebuttals in order by version.

Version 0:

Reviewer comments:

Reviewer #1

(Remarks to the Author)
In this proposed manuscript, Otwinowska et al. present a comprehensive list of 105 Klebsiella phage-derived depolymerases. Forty-six of them are novel, and their capsule specificity was verified. This catalogue expands our understanding of the diversity of phage depolymerases and provides a foundation for future studies in phage biology, enzyme evolution, and potential biotechnological applications.
However, several limitations reduce the immediate impact of the study. Most depolymerases were only tested using spot assays, without quantitative enzymology, kinetic measurements, or evaluation in more physiologically relevant systems. The host range of the originating phages is not explored, limiting insight into ecological relevance and potential therapeutic applicability. Medical relevance is also not demonstrated, and the experiments do not provide proof-of-concept for therapy or diagnostics. Structural interpretations based on AlphaFold predictions are informative but remain hypothetical without direct experimental confirmation of active sites or domain functions. The study does not provide mechanistic insight into how depolymerases evolve new specificities, such as through convergent evolution or modular domain swapping, nor the functional consequences of such changes.
Overall, while the catalogue represents a valuable biological resource that advances our understanding of phage depolymerase diversity, its main contribution is rather descriptive, with mechanistic interpretations remaining hypothetical until further experimentally validated. Furthermore, its predictive insights are limited, as the study lacks a robust predictive framework or validation using untested enzymes. The translational potential for therapeutic or diagnostic applications also remains to be demonstrated.

Specific comments are provided below:
- The abstract claims that the DepoCatalog includes 105 experimentally validated depolymerases. However, only 46 enzymes were directly tested and analyzed in this study. Presenting the full 105 as “experimentally validated” is misleading and overstates the scope of experimental work. It would be more accurate to clarify that 46 are newly characterized, while the remaining entries are included as part of the curated catalog.
- The introduction provides a comprehensive overview of Klebsiella capsules and phage depolymerases, focusing on structural and specificity diversity. However, the discussion of potential medical or therapeutic applications is minimal and should be extended.
- It is unclear how data from previously published depolymerases were integrated and compared with those generated in this study. The authors should clarify whether the same Klebsiella strains were used to assess specificity, whether the experimental conditions (e.g., spot assays, expression systems, enzyme concentrations) were standardized, and what criteria were applied to define enzymatic activity or substrate specificity. Line 479: The phrase “most using a large panel of K-types” is vague. The authors should clarify how this panel was designed and how it ensures a robust assessment of depolymerase specificity, including coverage of diverse capsule types and relevance for functional comparisons.
- Statements at lines from line 103 to 106 abd 106 to 108 would need a reference.
- Line 163–164: The terms “podovirus,” “siphovirus,” and “myovirus” are outdated, as these families were abolished in the revised ICTV taxonomy of Caudoviricetes. The manuscript should either adopt the current taxonomic framework or clearly state that these terms refer only to morphological types rather than formal viral families.
- The manuscript frequently refers to the “structure” of depolymerases in the catalog; however, these are all computational predictions generated by AlphaFold rather than experimentally determined structures. The authors should clarify this throughout the text, as repeatedly presenting predicted models as definitive structures may overstate the level of structural validation.

- Sentences 139–140 are unclear and should be reformulated for clarity.
- Lines 123–138: The numbered research questions in the introduction are somewhat unusual for a research article. These points should be rather intergrated into a coherent narrative that conveys the study's objectives. Furthermore, research questions 6 and 7 listed in the introduction are only partially or not addressed in the manuscript.
- Lines 148–178: This section would benefit from a more synthesized presentation. While it provides a detailed, case-by-case description of depolymerases and their target serotypes, the text is lengthy and highly descriptive, making it difficult to extract the main biological insights. A general summary figure illustrating the tested depolymerases, their activity profiles across different capsule types, and their phylogenetic relationships would greatly enhance clarity and help readers grasp the overall patterns.
- Figures 1 and 2: These figures appear redundant, as they convey very similar information.
- Line 279: The description "resembling a sort of 'pouf'" is colloquial and not appropriate for a scientific article. It should be replaced with a more precise structural description
- Several sections of the manuscript adopt a conversational or speculative tone, using rhetorical questions or informal phrases (e.g., "there is a big question mark if…?"). Rewriting these passages with more precise phrasing would improve readability

Reviewer #2

(Remarks to the Author)
The DepoCatalog manuscript is a large undertaking of generally high technical quality, aimed at cataloging, annotating, and analyzing the growing structural and functional diversity of Klebsiella bacteriophage depolymerases. In doing so, it combines results from large-scale, functionally validated experimental work with those from computational modeling. The authors aim to present an integrated framework of structure-function relationships that include sequence, structure, and biochemistry (i.e., capsule specificity). Overall, the project is very ambitious but well thought out. These types of manuscripts are often difficult to write (and to review!) due to the density of data involved. Providing summary tables in the main text could make the manuscript more accessible to general readers. Nonetheless, the findings presented here will greatly benefit research communities in phage biology, structural enzymology, and even antimicrobial development. The design of the study and the individual analyses are all generally well-conceived and the methodology is more or less sound. I really like that the authors clearly state seven questions in the introduction to be addressed and by the discussion they were either directly addressed or partially addressed. This resource should become foundational to future work on capsule-targeting enzymes. However, some aspects of presentation and methodological transparency, all detailed further below, could be improved to make the manuscript clearer and more accessible to most readers. Despite these relatively minor shortcomings, this is a high-impact contribution that will be of great interest and use to many in the field.

Areas that could be addressed/clarified:

- I anticipate that this manuscript would resonate with scientists from many disciplines, including many who may not be intimately familiar with Klebsiella biology. In particular, the distinction between K-type and KL-type strains is not clearly defined. It is stated that K1–K82 strains are experimentally verified serotypes, but then it just says that loci beyond KL100 remain uncharacterized, citing the K-PAM database. The authors should more clearly define what is meant by "K" and "KL" strains in a reader-oriented sentence.

- The authors do not make it clear exactly how many depolymerases they expressed and independently characterized against all the capsule types. The overall tone of the manuscript leads one to believe they did this for all 105 depolymerases. However, in lines 168-170 they indicate that some of enzymes were characterized in the literature and some were evaluated in this study. Digging into supplemental Table 2, specifically the tested K-type column, it would appear that less than half of the depolymerases were experimentally characterized in this study, being evaluated on K1-K82 and KL101-KL170. In fact, of those where characterization relied on the published literature, some were only characterized against a few, or even a single, capsule type. As a result, there is risk that the full host range may be underestimated for some of these depolymerases. The authors need to be more transparent in how many enzymes they experimentally characterized versus results extrapolated from the literature. They also need to more clearly state these as potential limitations of their conclusions.

- Figures 1 and 2 not only lack figure legends, they do not even have a title. Merged together, it was not even apparent where figure 1 ended and figure 2 began. Although many details for these figures are in the text of the manuscript, a figure legend providing relevant details would make it easier for the reader to interpret the figures independently.

- Likewise, the 52-page supplement is difficult to absorb. It lacks conventional figure numbering, legends, and table titles. Pages contain panel headers, but these are without explanatory captions. Moreover, not all panel headers contain the same data/information. I'm not suggesting that all 52 pages need figure legends, etc., but some of the data needs more information. Perhaps some sort of "how to read this supplement" section or contents page could be included.

- The manuscript relies on AlphaFold3 structural predictions for the different classifications of the depolymerases. However, the methods are a bit sparse and information about model construction is missing. As an example, the figures switch back and forth between monomeric and trimeric models, yet, the methods do not indicate whether models were built as monomers or multimers. This can drastically affect both the model, and the quality of the model (see the next bullet point). Some of the monomers shown are later discussed for their intersubunit features. Please clarify whether all enzymes were modeled as monomers or homotrimers, and where relevant, report interface-confidence metrics.

- As an extension of the above, the manuscript relies heavily on TM-score comparisons of different depolymerases. However, these comparisons are only as good as the quality of the structural models used to develop the TM scores. As the saying goes, garbage in = garbage out. The reader needs to know that quality models were used for the TM comparisons. At present, the TM-scores are qualitative rather than quantitatively validated. This could be enhanced if the authors reported the AlphaFold3 confidence metrics such as pLDDT and PAE values or provide some baseline cutoff required before performing the TM-score analysis. Providing mean pLDDT values per domain, representative PAE plots, and clarification on whether TM-scores were calculated from high-confidence regions or entire models would greatly improve the transparency and robustness of the manuscript.

- The manuscript presents a curated “catalog” of Klebsiella depolymerase enzymes. However, this catalog only exists in the supplemental files of the manuscript itself. There is no external repository, DOI, or searchable web interface. I leave it up to the editor whether this is a requirement, but it does seem odd to me to present a new curated catalog that isn’t really searchable or very useful to the outside community. At the least, I would suggest the authors provide links to resources such as Zenodo or Figshare with machine-readable figures and tables to provide a minimal searchable interface. The authors should also consider a Data Availability statement with such links to the full catalog.

Version 1:

Reviewer comments:

Reviewer #1

(Remarks to the Author)
We thank the authors for their thorough and detailed revisions in response to our earlier comments. The clarifications regarding experimentally characterized depolymerases, AlphaFold3 structural models, and expanded discussion of therapeutic relevance partially address our previous concerns. Nonetheless, several aspects require further elaboration to fully assess the representativeness, methodological transparency, and interpretative scope of the study.

Major comments:
- Dataset representativeness: The manuscript now presents 129 depolymerases, but it remains unclear how representative this collection is of the broader diversity of Klebsiella phage depolymerases. The selection relies largely on the authors’ isolates and literature-reported proteins, potentially introducing bias. The dataset is not benchmarked against public phage genomic datasets, making it difficult to assess whether the structural classification (5 classes, 14 subclasses) is generally applicable or primarily descriptive of the current collection. The scope and limitations should be clarified.
- Depolymerase vs. phage activity: The comparison of depolymerase activity with phage lysis is insufficiently described. The term “halo formation” is not defined, and no images or quantitative data are provided. Clearer methodology, representative images, and ideally some quantification are needed.
- DepoCat database: The database description reads more like software promotion than scientific reporting, with phrases such as “intuitive exploration” and “enhanced exploration experience.” Its utility is not demonstrated beyond hosting the compiled dataset. Importantly, the lack of a sequence-based search (e.g., BLAST or HMM) prevents users from comparing their own sequences, limiting practical use.
- Evolutionary claims and Figure 1: Statements suggesting that the C-terminus “drives” depolymerase evolution are not directly supported by the presented data. The manuscript infers the effects of domain shuffling versus point mutations without formal experimental or computational testing, so such claims should be tempered. Similarly, Figure 1 is titled as a “Framework of the diversity and evolution of depolymerases,” but the manuscript does not provide a generalizable conceptual or mechanistic framework. No rigorous evolutionary analyses—such as phylogenetic reconstruction, ancestral sequence inference, or selective pressure assessment—were performed. The study only presents sequence- and structure-based grouping and classification, making any claims about evolutionary relationships or dynamics tentative rather than systematically validated.
- In silico identification and diversity: The pipeline describes heuristic filters (size, annotation, domain homology, predicted β-helical structure) rather than a systematic assessment of depolymerase diversity. The dataset likely excludes divergent or novel enzymes. No analyses are provided to quantify coverage or sampling completeness.
- Sequence sources: It is unclear whether all publicly available Klebsiella phages were surveyed. Candidate proteins appear drawn from the authors’ collection, mined prophages, and literature-reported sequences. The resulting 129 depolymerases represent a pre-selected subset rather than the complete or unbiased diversity.

Minor comments – Methods / protein expression & activity:
- Cloning details: It is unclear whether full-length proteins or only domains/subdomains were cloned and expressed. Clarification would help interpret activity assays and domain-specific effects.
- Representative SDS-PAGE images are not shown. Including them would allow verification of protein size, purity, and expression levels.
- Exact enzyme concentrations and normalization across proteins of different expression/purification yields are not reported. Including this information would improve reproducibility and allow valid comparisons.

Reviewer #2

(Remarks to the Author)
I have read the manuscript thoroughly and the authors have addressed all of my comments and concerns, and as best as I can tell, they have also addressed the concerns of the other reviewer. While I am normally not a fan of adding additional authors and/or data to the manuscript that were not requested by the reviewers, in this case, the additional data added to the manuscript since the initial review only serve to increase the robustness of the document and database. I am particularly impressed with the web interface created in this latest revision, which makes the data accessible to all scientists in the field. I am convinced by the quality of the work and have no reservations with the manuscript. It is a significant contribution to the field. Bravo.

Open Access This Peer Review File is licensed under a Creative Commons Attribution 4.0 International License, which permits use, sharing, adaptation, distribution and reproduction in any medium or format, as long as you give appropriate credit to the original author(s) and the source, provide a link to the Creative Commons license, and indicate if changes were made.
In cases where reviewers are anonymous, credit should be given to 'Anonymous Referee' and the source.
The images or other third party material in this Peer Review File are included in the article's Creative Commons license, unless indicated otherwise in a credit line to the material. If material is not included in the article's Creative Commons license and your intended use is not permitted by statutory regulation or exceeds the permitted use, you will need to obtain permission directly from the copyright holder.
To view a copy of this license, visit https://creativecommons.org/licenses/by/4.0/

TO BOTH REVIEWERS

We thank the Reviewers for their important and constructive comments, allowing us to improve our work. In the revised manuscript, we have substantially expanded our experimental dataset. During the revision period, we identified additional **19** depolymerase candidates in our newly isolated phage collection (two additional co-authors, Maha Niazi and Sabrina Green). We prepared corresponding constructs and successfully expressed and purified recombinant proteins. Furthermore, following the Reviewers' recommendations, we also prepared constructs, recombinant proteins, and performed specificity assays for **18** selected literature-reported depolymerases for which no structural or enzymatic homologs were present among the depolymerases produced by our team. The remaining 39 overlapping depolymerases from the published references were assessed based on the details provided in the papers, and the results turned out to be consistent with those for enzymatic homologs tested in this study.

Among **129** identified phage-borne depolymerases (**72** from our collection and **57** enzymes tested in the literature), we have produced and tested **90** recombinant depolymerases (**72** from our collection and **18** from the literature). Based on this approach, we were able to map the enzyme specificity toward > **70** KL-types. The revised Supplementary Data 3 clearly differentiates (i) enzymes experimentally characterized in this study, and (ii) those for which data are based solely on published reports. This information is now also explicitly stated in the Methods and the Results sections for full transparency.

During the revision period, we also significantly enlarged the KL-type *Klebsiella spp*. collection for enzyme specificity testing. The ability to degrade the particular capsule of the KL-type *Klebsiella spp*. strains was screened now on K1-K82 defined serotypes and KL101-KL186 genetically identified isolates, with some exceptions KL106, KL129, KL147,KL150, KL152, KL154, KL156, KL157, KL159, KL160, KL162, KL171, KL172, KL175, KL176, KL179, KL180, KL182, KL185) (one additional co-author, Jens A. Hammerl). For some KL-locus and K-types, we include extra strains to determine the phage/protein activity range (Supplementary Data 1).

As part of a quality-control step prior to resubmission, we re-verified the KL>100 reference strain collection to ensure consistency between strain identity, phenotypic behaviour, and genomic data. Most of the KASPAH isolate genomes have been re-sequenced, and a *wzi* PCR assay was performed to double-check the isolates sensitive to depolymerase activity. During this process, we identified morphological heterogeneity in some isolates, such as those originally labelled as KL146 (INF117.1 and INF117.2). Resequencing confirmed that one colony corresponded to KL146 (INF117.1), whereas the second morphotype (INF117.2) was in fact KL3. This explains the previously observed unexpected activity of a K3-specific enzyme on the strain presumed to be KL146. Other examples: KL119 (INF226) turned out to be a K25; we replaced this strain with KSB1_8A, and the result for K25/KL119 has been changed to K25 only.

This approach allowed us to fill key gaps in the dataset and ensure that representative enzymes from all major capsule locus (KL) clusters were experimentally validated in a unified assay.

We also clearly highlight in the results that relying on literature-derived data for a subset of enzymes may underestimate (or occasionally overestimate) their true host range. We have also added a new Figure 1 representing the pipeline of our study.

Due to the above modifications, additional experiments and data extension, the manuscript has been substantially rewritten.

The heatmap Figure 2, combining enzyme activity toward specific capsule type and phage morphotype of all analysed enzymes, has been prepared.

Finally, we develop a publicly accessible interactive DepoCat dataset (https://depocat.uwr.edu.pl) for structural classification and comparison of newly identified depolymerases. The link and short info about the database have been added to the manuscript body.

The data generated in this study are presented in the supplementary files. The GenBank accession numbers are listed in Supplementary Data 3 and the Methods section. The depolymerases structure models in pdb files generated using AlphaFold3.0 and depolymerase characteristic metadata are available at DepoCat (depocat.uwr.edu.pl). Source data associated with this paper are available at 10.5281/zenodo.19052650.

All custom codes have been deposited at Zenodo and are publicly available at 10.5281/zenodo.19052650. Any additional information required to analyze the data reported in this publication is available from the lead contact upon request.

REVIEWER COMMENTS

We sincerely thank all Reviewers for their thorough evaluation of our manuscript and for the valuable comments provided. Their constructive suggestions have greatly helped us improve the clarity, depth, and overall quality of the revised work.

Reviewer #1 (Remarks to the Author):

Comment 1

In this proposed manuscript, Otwinowska et al. present a comprehensive list of 105 Klebsiella phage-derived depolymerases. Forty-six of them are novel, and their capsule specificity was verified. This catalogue expands our understanding of the diversity of phage depolymerases and provides a foundation for future studies in phage biology, enzyme evolution, and potential biotechnological applications. However, several limitations reduce the immediate impact of the study. Most depolymerases were only tested using spot assays, without quantitative enzymology, kinetic measurements, or evaluation in more physiologically relevant systems.

Response 1

We thank the Reviewer for this valuable comment. We fully agree that our study does not include quantitative enzymology, kinetic measurements, or evaluation of depolymerases in physiologically complex environments. Importantly, this was not the aim of the present work. Our objective was to provide a comprehensive catalogue of substrate specificity and structural diversity of currently known Klebsiella phage depolymerases, rather than to characterize their enzymatic kinetics or therapeutic performance.

Systematic kinetic analyses under varying environmental or physiological conditions are beyond the scope of this manuscript. Such experiments are typically performed for individual enzymes, and indeed, we have conducted these studies elsewhere, focusing on serum sensitization, enhancement of phagocytosis and intracellular killing, and activity in *Galleria mellonella* infection models. These aspects, together with therapeutic proof-of-concept studies, are better suited for dedicated follow-up papers, rather than a broad comparative catalogue such as the one presented here.

The current work aims specifically to highlight the range of substrate specificities, structural diversification, and evolutionary relationships within the expanding landscape of phage depolymerases. Nonetheless, we have expanded the Discussion to emphasize that practical applications in therapy and diagnostics—as well as protein engineering approaches to broaden substrate range—represent important next steps for future research.

Comment 2

The host range of the originating phages is not explored, limiting insight into ecological relevance and potential therapeutic applicability. Medical relevance is also not demonstrated, and the experiments do not provide proof-of-concept for therapy or diagnostics.

Response 2

We thank the Reviewer for raising this important point. In our study, the activity range of depolymerases originating from our lytic phage collection was systematically compared with the lytic host range of the corresponding phage (if available) to ensure biological consistency and to better understand ecological relevance. Moreover, being aware of intra-serotype variability, particularly in serotypes such as K47, we performed additional specificity tests using multiple independent bacterial representatives available in our collection. In the case of the prophage origin of the produced enzymes, we were not able to compare phage-depolymerase activity.

These results have been incorporated into the revised version of the manuscript, providing a more comprehensive characterization of phage–enzyme specificity patterns and strengthening conclusions regarding potential therapeutic and diagnostic applications (Supplementary Data 2).

Comment 3

Structural interpretations based on AlphaFold predictions are informative but remain hypothetical without direct experimental confirmation of active sites or domain functions. The study does not provide mechanistic insight into how depolymerases evolve new specificities, such as through convergent evolution or modular domain swapping, nor the functional consequences of such changes. Overall, while the catalogue represents a valuable biological resource that advances our understanding of phage depolymerase diversity, its main contribution is rather descriptive, with mechanistic interpretations remaining hypothetical until further experimentally validated. Furthermore, its predictive insights are limited, as the study lacks a robust predictive framework or validation using untested enzymes. The translational potential for therapeutic or diagnostic applications also remains to be demonstrated.

Response 3

We thank the referee for finding our work a tool to expand our understanding of the diversity of phage depolymerases and provides a foundation for future studies in phage biology, enzyme evolution, and potential biotechnological applications. This was exactly our goal.

We agree that experimental structures would provide more precise information. However, we have determined more depolymerase structures over the years, before and after the discovery of AlphaFold, and others are in progress (Squeglia et al 2020, Maciejewska et al 2023, Napolitano et al 2025). While comparing our experimental structures with Alphafold3.0 structural models, we found they completely overlap, with root-mean-square deviations (RMSD) computed on backbone atoms below 1 Å. Based on this finding, we realised that Alphafold3.0 predictions are highly meaningful for this class of enzymes.

In the revised manuscript, we have substantially expanded our experimental dataset. All proteins were modelled as homotrimers, and interface-confidence metrics have been added to Supplementary Data 4 and 6. The AlphaFold3 confidence metrics, such as pLDDT and PAE values. For structural comparison between proteins, we considered only the modelled central and C-terminal domains with pLDDT > 70, eliminating the N-terminus (virion-anchoring part of the RBP) from the full homotrimer in PyMOL. We provided mean pLDDT values per domain. The clarification on whether TM-scores were calculated from high-confidence regions or entire models was also included in the data set and results to improve the transparency and robustness of the manuscript.

We fully agree that identifying catalytic residues and experimentally validating domain functions—particularly through targeted mutagenesis—is essential for establishing mechanistic insight. Indeed, such studies have been a long-standing part of our research program, and for several individual depolymerases we have already investigated systematically in our laboratory (Squeglia et al 2020, Maciejewska et al 2023, Napolitano et al 2025, Latka et al, 2021 & 2025, Latka et al., 2026, two manuscripts in revision).

In addition, based on our long-standing experience working with phage depolymerases, we would like to note that generating catalytic-site mutants does not always yield interpretable or expected results. This is because substrate-binding determinants may reside in regions distinct from the catalytic residues, and both contribute to activity. In our hands, even single amino-acid substitutions—such as standard alanine-scanning mutations—frequently destabilize the fold of the monomer, which in turn disrupts trimerization, a prerequisite for proper enzyme function. We successfully applied catalytic residue probing to identify the catalytic center.

In complementary work, we have investigated the functions of individual domains by performing domain-shuffling experiments at the protein and phage levels. However, even for one or two representative enzymes, such experiments are highly time-consuming, and we were able to prove that functional enzymes exist only when the Central + C- term domains pair is horizontally exchanged (Latka et al, 2021 & 2025, Latka et al., 2026, two manuscripts in revision). Similarly, together with Rita Berisio (co-author), we have attempted co-crystallization of depolymerases with their substrates, to prove sugar-binding sites, but—as is well established—such structural studies require substantial effort even for a single protein and often present significant technical challenges.

We want to emphasize that the aim of the present manuscript is different. Here, we sought to reveal the broader landscape of depolymerase diversity, which only becomes apparent when analysing a large and heterogeneous collection of enzymes with diverse specificities and evolutionary origins. This large-scale comparative approach enabled us to uncover two main strategies for capsular switch: (1) domain exchange, but only in pairs (the Central + C- term domains), (2) the conserved central domain existence combined with the slight modifications within the C-terminal domains.

The latter pattern was observed for depolymerases specific to KL111 and K25, as well as those targeting K3.

The spatial modification and surface charge distribution—as revealed by structural modelling—are likely to be key contributors to either substrate-range expansion or shifts in substrate recognition. While we agree that these interpretations remain hypothetical without direct mutational validation, they provide testable hypotheses and conceptual frameworks that guide and prioritize future experimental work.

We are grateful for the reviewer's constructive comments, which motivated us to extend the dataset substantially. In the revised manuscript, we now include a much broader set of depolymerases that we have recently tested in our laboratory, alongside enzymes previously reported in the literature. This expanded dataset strengthens the biological and comparative value of the catalogue.

We have tried to address referees' concerns to improve the quality of our manuscript.

Specific comments are provided below:

Comment 4 & 6

4. The abstract claims that the DepoCatalog includes 105 experimentally validated depolymerases. However, only 46 enzymes were directly tested and analyzed in this study. Presenting the full 105 as "experimentally validated" is misleading and overstates the scope of experimental work. It would be more accurate to clarify that 46 are newly characterized, while the remaining entries are included as part of the curated catalog.

6. It is unclear how data from previously published depolymerases were integrated and compared with those generated in this study. The authors should clarify whether the same Klebsiella strains were used to assess specificity, whether the experimental conditions (e.g., spot assays, expression systems, enzyme concentrations) were standardized, and what criteria were applied to define enzymatic activity or substrate specificity. Line 479: The phrase "most using a large panel of K-types" is vague. The authors should clarify how this panel was designed and how it ensures a robust assessment of depolymerase specificity, including coverage of diverse capsule types and relevance for functional comparisons.

Response to 4 & 6

We thank the Reviewers for these important and constructive comments, allowing us to improve our work. In the revised manuscript, we have substantially expanded our experimental dataset. During the revision period, we identified additional **19** depolymerase candidates in our newly isolated phage collection (two additional co-authors). We prepared corresponding constructs and successfully expressed and purified recombinant proteins. Furthermore, following the Reviewer's recommendations, we also prepared constructs, recombinant proteins, and performed specificity assays for **18** selected literature-reported depolymerases for which no structural or enzymatic homologs were present among the depolymerases produced by our team (Supplementary Data 3, Supplementary Data 5). The remaining (overlapping) 39 depolymerases from the published references were assessed based on the details provided in these manuscripts, and the results proved to be consistent with those for enzymatic analogs tested in this study.

Among **129** identified phage-borne depolymerases (**72** from our collection and **57** enzymes tested in the literature), we have produced and tested **90** recombinant depolymerases (**72** from our collection and **18** from the literature). Based on this approach, we were able to map the enzyme specificity toward > **70** KL-types. The revised Supplementary Data 3 clearly differentiates (i) enzymes experimentally characterized in this study, and (ii) those for which data are based solely on published reports. This information is now also explicitly stated in the Methods and the Results sections for full transparency.

We concur that the original phrasing "most using a large panel of K-types" was insufficiently precise. We have now clarified in the manuscript how the KL panel used for assessing depolymerase specificity was designed.

We have also added a new Figure 1 representing the pipeline of our study.

We hope that these additions fully address the Reviewer's concerns and significantly strengthen the manuscript.

Comment 5

The introduction provides a comprehensive overview of Klebsiella capsules and phage depolymerases, focusing on structural and specificity diversity. However, the discussion of potential medical or therapeutic applications is minimal and should be extended.

Response 5

The referee is right. We have extended the aspect of potential medical or therapeutic applications, as requested.

Comment 7

Statements at lines from line 103 to 106 and 106 to 108 would need a reference.

Response 7

We have included references. We apologise for this oversight.

Comment 8

Line 163–164: The terms “podovirus,” “siphovirus,” and “myovirus” are outdated, as these families were abolished in the revised ICTV taxonomy of Caudoviricetes. The manuscript should either adopt the current taxonomic framework or clearly state that these terms refer only to morphological types rather than formal viral families.

Response 8

Thank you for this comment. We fully agree that the terms *Podoviridae*, *Siphoviridae*, and *Myoviridae* no longer correspond to valid families in the current ICTV taxonomy of class *Caudoviricetes*. However, the non-italicized designations podovirus, myovirus and siphovirus are morphological identifiers that have no taxonomic meaning but do have a clear morphological meaning. We systematically use the correct ICTV classification throughout the manuscript (italicized *Webervirus*, *Drulisvirus* etc...). Morphotype naming was consulted with our co-author, Rob Lavigne, a former member of the ICTV Executive Committee, responsible for bacteriophage taxonomy.

Comment 9

The manuscript frequently refers to the “structure” of depolymerases in the catalog; however, these are all computational predictions generated by AlphaFold rather than experimentally determined structures. The authors should clarify this throughout the text, as repeatedly presenting predicted models as definitive structures may overstate the level of structural validation.

Response 9

The referee is right. We have now replaced the term “structure” with “structural model” or “predicted structure”.

Comment 10

Sentences 139–140 are unclear and should be reformulated for clarity.

Response 10

We have rewritten the text for better clarity, as requested.

Comment 11

Lines 123–138: The numbered research questions in the introduction are somewhat unusual for a research article. These points should be rather integrated into a coherent narrative that conveys the study's objectives. Furthermore, research questions 6 and 7 listed in the introduction are only partially or not addressed in the manuscript.

Response 11

According to this remark, we have reworked this section into a shortened sentence.

Comment 12

Lines 148–178: This section would benefit from a more synthesized presentation. While it provides a detailed, case-by-case description of depolymerases and their target serotypes, the text is lengthy and highly descriptive, making it difficult to extract the main biological insights. A general summary figure illustrating the tested depolymerases, their activity profiles across different capsule types, and their phylogenetic relationships would greatly enhance clarity and help readers grasp the overall patterns.

Response 12

We thank the referee for this comment. We agree that a conceptual figure would help readers to extract the main biological insights of our work. The heatmap combining enzyme activity toward specific capsule type and phage morphotype of all analysed enzymes has been prepared (Figure 2). The results section has been shortened accordingly.

Comment 13

Figures 1 and 2: These figures appear redundant, as they convey very similar information.

Response 13

We understand this concern. However, the two figures describe different characteristics of depolymerases, which are important to understand their structure-function relationship. We first catalogued based on depolymerase substrate specificity to assess the structural divergence and sequence variability within particular capsule tropisms (old Figure 1). Differently, in the old Figure 2, we classify based on their structural features. This classification showed that depolymerases can be clustered into five principal structural classes, based on their overall architectural features. This two-fold classification is fundamental to understanding correlations between depolymerase structural architecture and substrate specificity. Following the Reviewer's remark, we have modified old Figure 1 into new Figure 2, as a heatmap showing the activity of all depolymerases, morphotype of the phage producing the enzyme versus targeted K-type. The structure atlas has been prepared in a simple version Figure 3 and a detailed version Supplementary Figure 5.

Comment 14

Line 279: The description “resembling a sort of ‘pouf’” is colloquial and not appropriate for a scientific article. It should be replaced with a more precise structural description

Response 14

The referee is correct. We have replaced this description with a “pouf-like” shape.

Comment 15

Several sections of the manuscript adopt a conversational or speculative tone, using rhetorical questions or informal phrases (e.g., “there is a big question mark if…?”). Rewriting these passages with more precise phrasing would improve readability

Response 15

We have rewritten the text for better clarity, as requested.

We hope this addresses your concern and improves the overall clarity and readability of the manuscript.

Reviewer #2 (Remarks to the Author):

The DepoCatalog manuscript is a large undertaking of generally high technical quality, aimed at cataloging, annotating, and analyzing the growing structural and functional diversity of Klebsiella bacteriophage depolymerases. In doing so, it combines results from large-scale, functionally validated experimental work with those from computational modeling. The authors aim to present an integrated framework of structure-function relationships that include sequence, structure, and biochemistry (i.e., capsule specificity). Overall, the project is very ambitious but well thought out. These types of manuscripts are often difficult to write (and to review!) due to the density of data involved. Providing summary tables in the main text could make the manuscript more accessible to general readers. Nonetheless, the findings presented here will greatly benefit research communities in phage biology, structural enzymology, and even antimicrobial development. The design of the study and the individual analyses are all generally well-conceived and the methodology is more or less sound. I really like that the authors clearly state seven questions in the introduction to be addressed and by the discussion they were either directly addressed or partially addressed. This resource should become foundational to future work on capsule-targeting enzymes. However, some aspects of presentation and methodological transparency, all detailed further below, could be improved to make the manuscript clearer and more accessible to most readers. Despite these relatively minor shortcomings, this is a high-impact contribution that will be of great interest and use to many in the field. Areas that could be addressed/clarified:

Comment 1

I anticipate that this manuscript would resonate with scientists from many disciplines, including many who may not be intimately familiar with Klebsiella biology. In particular, the distinction between K-type and KL-type strains is not clearly defined. It is stated that K1–K82 strains are experimentally verified serotypes, but then it just says that loci beyond KL100 remain uncharacterized, citing the K-PAM database. The authors should more clearly define what is meant by “K” and “KL” strains in a reader-oriented sentence.

Response 1

Thank you very much for this insightful comment. We agree that the distinction between “K” and “KL” types may not be sufficiently clear for readers who are not specialists in Klebsiella biology. In the revised manuscript, we have added a concise, reader-oriented explanation.

Specifically, we now clarify that “K types” refer to the historically defined serologically determined capsule serotypes (K1–K82), established through experimental serological assays, whereas “KL types” refer to capsule locus types identified through genomic analysis, as introduced with the Klebsiella K-locus (KL) nomenclature system. We also explain that KL loci beyond KL100 are predicted from genomic assemblies but have not yet been characterized experimentally (with some exceptions), consistent with the CPS structure provided in the K-PAM database.

We have added these definitions to the Introduction to ensure that readers from diverse disciplines can easily understand the terminology.

Comment 2

The authors do not make it clear exactly how many depolymerases they expressed and independently characterized against all the capsule types. The overall tone of the manuscript leads one to believe they did this for all 105 depolymerases. However, in lines 168-170 they indicate that some of enzymes were characterized in the literature and some were evaluated in this study. Digging into supplemental Table 2, specifically the tested K-type column, it would appear that less than half of the depolymerases were experimentally characterized in this study, being evaluated on K1-K82 and KL101-KL170. In fact, of those where characterization relied on the published literature, some were only characterized against a few, or even a single, capsule type. As a result, there is risk that the full host range may be underestimated for some of these depolymerases. The authors need to be more transparent in how many enzymes they experimentally characterized versus results extrapolated from the literature. They also need to more clearly state these as potential limitations of their conclusions.

Response 2

We thank the Reviewer for this important and constructive comment. In the revised manuscript, we have substantially expanded our experimental dataset. During the revision period, we identified additional depolymerase candidates in our newly isolated phage collection, prepared corresponding constructs, and successfully expressed and purified these recombinant proteins. We experimentally characterized their capsule specificity and included these new data in the updated Results and Supplementary materials.

Furthermore, following the Reviewers' recommendations, we also prepared constructs, recombinant proteins, and performed specificity assays for **18** of the selected literature-reported depolymerases for which no structural or enzymatic homologs were present among the depolymerases produced by our team. This allowed us to fill key gaps in the dataset and ensure that representative enzymes from all major capsule locus (KL) clusters were experimentally validated in a unified assay.

In total, we have now produced and tested 90 recombinant depolymerases specific toward > 70 KL types. The revised Supplementary Data 3 clearly differentiates (i) enzymes experimentally characterized in this study, and (ii) those for which data are based solely on published reports. This information is now also explicitly stated in the Methods and the Results sections for full transparency.

We also now clearly highlight in the results that relying on literature-derived data for a subset of enzymes may underestimate (or occasionally overestimate) their true host range, and we explicitly present this as a limitation of our conclusions. We have also added a graphical abstract representing the pipeline of our study (new Figure 1).

We hope that these additions fully address the Reviewer's concerns and significantly strengthen the manuscript.

Comment 3

Figures 1 and 2 not only lack figure legends, they do not even have a title. Merged together, it was not even apparent where figure 1 ended and figure 2 began. Although many details for these figures are in the text of the manuscript, a figure legend providing relevant details would make it easier for the reader to interpret the figures independently.

Response 3

Indeed, we thank the Reviewer for this comment. In the revised manuscript, Figures have been fully updated: we added clear titles, complete figure legends, and all missing elements, and we separated the figures to ensure they are easily distinguishable. Following the Reviewer's remark, we have modified old Figure 1 into new Figure 2, as a heatmap showing the activity of all depolymerases, morphotype of the phage producing the enzyme versus targeted K-type. The structure atlas has been prepared in a simple version Figure 3 and a detailed version Supplementary Figure 5.

Comment 4

Likewise, the 52-page supplement is difficult to absorb. It lacks conventional figure numbering, legends, and table titles. Pages contain panel headers, but these are without explanatory captions. Moreover, not all panel headers contain the same data/information. I'm not suggesting that all 52 pages need figure legends, etc., but some of the data needs more information. Perhaps some sort of "how to read this supplement" section or contents page could be included.

Response 4

Indeed, we thank the Reviewer for this comment. In the revised manuscript, Supplementary Information has been fully updated: we added a clear title, "how to read this supplement" slide, and all missing elements.

Comment 5

The manuscript relies on AlphaFold3 structural predictions for the different classifications of the depolymerases. However, the methods are a bit sparse and information about model construction is missing. As an example, the figures switch back and forth between monomeric and trimeric models, yet, the methods do not indicate whether models were built as monomers or multimers. This can drastically affect both the model, and the quality of the model (see the next bullet point). Some of the monomers shown are later discussed for their intersubunit features. Please clarify whether all enzymes were modeled as monomers or homotrimers, and where relevant, report interface-confidence metrics.

Comment 6

As an extension of the above, the manuscript relies heavily on TM-score comparisons of different depolymerases. However, these comparisons are only as good as the quality of the structural models used to develop the TM scores. As the saying goes, garbage in = garbage out. The reader

needs to know that quality models were used for the TM comparisons. At present, the TM-scores are qualitative rather than quantitatively validated. This could be enhanced if the authors reported the AlphaFold3 confidence metrics such as pLDDT and PAE values or provide some baseline cutoff required before performing the TM-score analysis. Providing mean pLDDT values per domain, representative PAE plots, and clarification on whether TM-scores were calculated from high-confidence regions or entire models would greatly improve the transparency and robustness of the manuscript.

Response to 5 and 6

We thank the Reviewer for this important and constructive comment. In the revised manuscript, we have substantially expanded our experimental dataset. All proteins were modelled as homotrimers, and interface-confidence metrics have been added to Supplementary Data 4. The AlphaFold3.0 confidence metrics, such as pLDDT and PAE values, have been provided. For structural comparison between proteins, we considered only the modelled central and C-terminal domains with pLDDT > 70, eliminating the N-terminus (virion-anchoring part of the RBP) from the full homotrimer in PyMOL. We provided mean pLDDT values per domain. The clarification on whether TM-scores were calculated from high-confidence regions or entire models was also included in the data set and results to improve the transparency and robustness of the manuscript.

Comment 7

The manuscript presents a curated "catalog" of Klebsiella depolymerase enzymes. However, this catalog only exists in the supplemental files of the manuscript itself. There is no external repository, DOI, or searchable web interface. I leave it up to the editor whether this is a requirement, but it does seem odd to me to present a new curated catalog that isn't really searchable or very useful to the outside community. At the least, I would suggest the authors provide links to resources such as Zenodo or Figshare with machine-readable figures and tables to provide a minimal searchable interface. The authors should also consider a Data Availability statement with such links to the full catalog.

Response 7

We thank the Reviewer for this important and constructive comment. We have deposited the dataset of all depolymerases into the open-access server DepoCat (https://depocat.uwr.edu.pl), for structural classification and comparison of newly identified depolymerases. The link and short info about the database have been added to the manuscript body.

The data generated in this study are presented in the supplementary files. The GenBank accession numbers are listed in Supplementary Data 3. We also prepared Supplementary Data 6. providing AlphaFold3.0 models with pLDDT score overlaid from two viewing angles, and the Predicted aligned error (PAE) of analyzed depolymerases.

The depolymerases structure models in pdb files generated using AlphaFold3.0 and depolymerase characteristic metadata are available at DepoCat (depocat.uwr.edu.pl). Source data associated with this paper are available at 10.5281/zenodo.19052650.

All custom codes have been deposited at Zenodo and are publicly available at 10.5281/zenodo.19052650. Any additional information required to analyze the data reported in this publication is available from the lead contact upon request.

REVIEWERS' COMMENTS

We want to thank the Reviewers for constructive and insightful comments, which have significantly improved the quality of our work.

In this revised submission, we have carefully addressed Reviewer 1's suggestions and incorporated additional data as recommended. We believe that the expanded dataset, together with the clearer presentation of our findings, has strengthened the manuscript and improved its clarity and impact. We also thank Reviewer 2 for the very positive assessment of our revised manuscript and for recognizing the added value of the additional data and the web interface. We appreciate the Reviewer's confidence in the quality and significance of our work.

We also note that the revised manuscript includes updated phage protein and gene numbering following recent GenBank submissions of KLEO7, KLEO25, KLEO26, and KluzKp13 phages.

Reviewer #1 (Remarks to the Author):

We thank the authors for their thorough and detailed revisions in response to our earlier comments. The clarifications regarding experimentally characterized depolymerases, AlphaFold3 structural models, and expanded discussion of therapeutic relevance partially address our previous concerns. Nonetheless, several aspects require further elaboration to fully assess the representativeness, methodological transparency, and interpretative scope of the study.

Major comments:

1. Dataset representativeness: The manuscript now presents 129 depolymerases, but it remains unclear how representative this collection is of the broader diversity of Klebsiella phage depolymerases. The selection relies largely on the authors' isolates and literature-reported proteins, potentially introducing bias. The dataset is not benchmarked against public phage genomic datasets, making it difficult to assess whether the structural classification (5 classes, 14 subclasses) is generally applicable or primarily descriptive of the current collection. The scope and limitations should be clarified.

Response to 1:

We fully agree with the Reviewer that the current collection does not capture the full diversity of Klebsiella phage depolymerases, and we expect the possibility that additional structural variants will be identified in the future. The dataset is based only on experimentally characterized enzymes derived from our isolates and those available in the literature, which, of course, introduces some degree of sampling bias.

Nevertheless, based on shared domain organization and general structural features, we consider the proposed five major classes to represent a reasonable and biologically meaningful initial framework for lyase-topology depolymerases. As with any developing database, an initial classification is necessary to organize existing knowledge and facilitate comparison across studies. We anticipate that future discoveries of new enzymes and experimentally validated activities will allow expansion of the proposed classification scheme. We have clarified the scope and limitations of the dataset accordingly in the revised manuscript.

According to reviewer comments, we have made three targeted changes. First, we have extended the opening paragraph of the Results. Second, we have added a dedicated paragraph to the Discussion, that contextualises the scope of the collection explicitly: acknowledging that it derives from curated rather than exhaustively surveyed sources, and that the observation of multiple structurally distinct depolymerases targeting the same KL-type suggests many depolymerases remain to be discovered. Third, the exact KL-type count has been restated in the abstract and Figure 1 for consistency.

2. Depolymerase vs. phage activity: The comparison of depolymerase activity with phage lysis is insufficiently described. The term "halo formation" is not defined, and no images or quantitative data are provided. Clearer methodology, representative images, and ideally some quantification are needed.

Response to 2: We thank the Reviewer for this comment. The methodological details clarifying the comparison between depolymerase activity and phage lysis, including the definition of "halo formation," have now been added to the Methods section. In addition, representative images illustrating phage activity with halo formation, as well as recombinant enzyme halo visualization, have been included in the Supplementary Figure 12 to support qualitative interpretation of the results.

3. DepoCat database: The database description reads more like software promotion than scientific reporting, with phrases such as "intuitive exploration" and "enhanced exploration experience." Its utility is not demonstrated beyond hosting the compiled dataset. Importantly, the lack of a sequence-based search (e.g., BLAST or HMM) prevents users from comparing their own sequences, limiting practical use.

Response to 3:

We thank the Reviewer for this comment. DepoCat currently functions as a curated database and visualization platform for experimentally validated depolymerases. Based on this resource, we are still developing a complementary tool that enables enzyme comparison; however, because this tool relies on structural similarity, prior structure prediction (e.g. using AlphaFold) is required to generate the necessary input files. At this

stage, we therefore limit the platform to hosting and visualizing proteins that have been experimentally tested. Importantly, in the current version of the database, all associated data—including amino acid and nucleotide sequences as well as protein structures—are fully available and can be used by users for independent analyses and comparisons. The database will be continuously updated as new experimentally verified enzymes become available. We have modified that description in the results section accordingly.

4. Evolutionary claims and Figure 1: Statements suggesting that the C-terminus "drives" depolymerase evolution are not directly supported by the presented data. The manuscript infers the effects of domain shuffling versus point mutations without formal experimental or computational testing, so such claims should be tempered. Similarly, Figure 1 is titled as a "Framework of the diversity and evolution of depolymerases," but the manuscript does not provide a generalizable conceptual or mechanistic framework. No rigorous evolutionary analyses—such as phylogenetic reconstruction, ancestral sequence inference, or selective pressure assessment—were performed. The study only presents sequence- and structure-based grouping and classification, making any claims about evolutionary relationships or dynamics tentative rather than systematically validated.

Response to 4:

Indeed, the Reviewer is right, and we agree that the evolutionary claims in the original manuscript were too strong given the presented data. We have therefore modified these statements, now limiting our conclusions to descriptive sequence- and structure-based observations and avoiding mechanistic or directional evolutionary interpretations, but only suggesting possible pathways.

Consistent with this revision, Figure 1 has been modified and retitled "A structure-based framework for depolymerase diversity" to emphasize structural diversity and classification rather than evolutionary framework or dynamics. We have also acknowledged the absence of formal evolutionary analyses in the revised manuscript, mainly in the Discussion part.

5. In silico identification and diversity: The pipeline describes heuristic filters (size, annotation, domain homology, predicted β-helical structure) rather than a systematic assessment of depolymerase diversity. The dataset likely excludes divergent or novel enzymes. No analyses are provided to quantify coverage or sampling completeness.

6. Sequence sources: It is unclear whether all publicly available Klebsiella phages were surveyed. Candidate proteins appear drawn from the authors' collection, mined

prophages, and literature-reported sequences. The resulting 129 depolymerases represent a pre-selected subset rather than the complete or unbiased diversity.

Response to 5 and 6:

Indeed, our search was based on the previous assumption and discoveries of RBPs with depolymerase activity showing specific features (size, annotation, domain homology, predicted β-helical structure, lyase topology) (Latka et al., Frontiers in Microbiology 2019).

We fully agree with the Reviewer that the current collection does not cover the full diversity of *Klebsiella* phage depolymerases, and we indeed expect that additional structural variants will be identified in the future. This study was not designed to quantify coverage or sampling completeness of all potential depolymerase structure versions.

We thank the reviewer for highlighting this limitation. We have added a sentence in the In silico identification subsection of the Methods explicitly acknowledging that the pipeline relies on heuristic criteria — including size thresholds, annotation as tail fiber or tail spike proteins, and homology to known enzymatic domains — and that enzymes lacking these annotations or possessing atypical domain architectures not recognised by the homology-based tools employed may not be captured by this approach.

In a parallel study recently published by our group (Otwinowska et al., *PLoS Biol 24(4): e3003716 (2026)*, we performed a blind GWAS-based search for potential capsule-targeting enzymes encoded in prophages (around 4K Klebsiella genomes and 8K prophages analysed). This analysis revealed significant associations not only for lyase-type depolymerases but also for SGNH hydrolases, demonstrating that the diversity of phage-borne enzymes that potentially degrade the capsular polysaccharide is broader than previously assumed.

We have clarified this scope and its limitations in the revised manuscript, particularly at the end of the discussion.

Minor comments – Methods / protein expression & activity:
7. Cloning details: It is unclear whether full-length proteins or only domains/subdomains were cloned and expressed. Clarification would help interpret activity assays and domain-specific effects.

Response to 7: We thank the Reviewer for this comment. All proteins used in this study were produced and tested as full-length versions. The domain architectures shown in the manuscript were assigned from in silico analyses. We have clarified this in the revised manuscript to avoid confusion when interpreting the activity assays.

8. Representative SDS-PAGE images are not shown. Including them would allow verification of protein size, purity, and expression levels.

Response to 8: According to the Reviewer's remark and the Author Checklist requirements (we strongly encourage the use of repositories for large datasets or source data due to size considerations), representative SDS–PAGE images and bacterial lawn halo formation photos for each produced protein are now available in the Zenodo repository (https://doi.org/10.5281/zenodo.19927711).

9. Exact enzyme concentrations and normalization across proteins of different expression/purification yields are not reported. Including this information would improve reproducibility and allow valid comparisons.

Response to 9: We thank the Reviewer for this comment. In practice, expression and purification yields vary between proteins and production batches and depend on multiple experimental factors (e.g. culture conditions, media, vectors, and reagents), making exact normalization of enzyme concentrations across different proteins impractical. Importantly, our assays qualitatively assessed depolymerase activity (presence vs. absence), rather than enabling strict quantitative comparisons. Notably, clear halo formation was consistently observed even when purified proteins were present at very low levels and not prominently visible on SDS–PAGE, reflecting the high catalytic efficiency of these enzymes. We have clarified this point in the revised manuscript (Methods section) and added the representative SDS–PAGE images and visualized halo formation for each produced protein in the Zenodo repository (https://doi.org/10.5281/zenodo.19927711).

Reviewer #2 (Remarks to the Author):

I have read the manuscript thoroughly and the authors have addressed all of my comments and concerns, and as best as I can tell, they have also addressed the concerns of the other reviewer. While I am normally not a fan of adding additional authors and/or data to the manuscript that were not requested by the reviewers, in this case, the additional data added to the manuscript since the initial review only serve to increase the robustness of the document and database. I am particularly impressed with the web interface created in this latest revision, which makes the data accessible to all scientists in the field. I am convinced by the quality of the work and have no reservations with the manuscript. It is a significant contribution to the field. Bravo.

Response: We thank the Reviewer for the very positive assessment of our revised manuscript and for recognizing the added value of the additional data and the web interface. We appreciate the Reviewer's confidence in the quality and significance of our work.
